# Rapid expansion and specialization of the TAS2R bitter taste receptor family in amphibians

**DOI:** 10.1371/journal.pgen.1011533

**Published:** 2025-01-31

**Authors:** Kathleen W. Higgins, Akihiro Itoigawa, Yasuka Toda, Daniel Winston Bellott, Rachel Anderson, Roberto Márquez, Jing-Ke Weng

**Affiliations:** 1 Whitehead Institute of Biomedical Research, Cambridge, Massachusetts, United States of America; 2 Department of Biology, Massachusetts Institute of Technology, Cambridge, Massachusetts, United States of America; 3 Department of Chemistry and Chemical Biology, Northeastern University, Boston, Massachusetts, United States of America; 4 Department of Bioengineering, Northeastern University, Boston, Massachusetts, United States of America; 5 Department of Chemical Engineering, Northeastern University, Boston, Massachusetts, United States of America; 6 Institute for Plant-Human Interface, Northeastern University, Boston, Massachusetts, United States of America; 7 Japan Society for the Promotion of Sciences, Chiyoda-ku, Tokyo, Japan; 8 Department of Agricultural Chemistry, School of Agriculture, Meiji University, Kawasaki, Kanagawa, Japan; 9 Department of Biological Sciences, Virginia Tech, Blacksburg, Virginia, United States of America; Universidad de los Andes Facultad de Ciencias, COLOMBIA

## Abstract

TAS2Rs are a family of G protein-coupled receptors that function as bitter taste receptors in vertebrates. Mammalian TAS2Rs have historically garnered the most attention, leading to our understanding of their roles in taste perception relevant to human physiology and behaviors. However, the evolution and functional implications of TAS2Rs in other vertebrate lineages remain less explored. Here, we identify 9,291 TAS2Rs from 661 vertebrate genomes. Large-scale phylogenomic analyses reveal that frogs and salamanders contain unusually high TAS2R gene content, in stark contrast to other vertebrate lineages. In most species, TAS2R genes are found in clusters; compared to other vertebrates, amphibians have additional clusters and more genes per cluster. We find that vertebrate TAS2Rs have few one-to-one orthologs between closely related species, although total TAS2R count is stable in most lineages. Interestingly, TAS2R count is proportional to the receptors expressed solely in extra-oral tissues. In vitro receptor activity assays uncover that many amphibian TAS2Rs function as tissue-specific chemosensors to detect ecologically important xenobiotics.

## Author summary

Most vertebrates are able to taste five cardinal tastes: sweet, sour, salty, umami, and bitter, using a specialized suite of receptor proteins. Amphibians have a much larger repertoire of bitter taste receptors, called TAS2Rs, than other vertebrates. We investigated the evolutionary history of TAS2Rs across vertebrates, and found that frogs and salamanders experienced a ~10-fold increase in the number of receptors, possibly associated with shifts in the selective pressures for the perception of bitter chemicals. Furthermore, this increase may have been facilitated by the TAS2Rs’ location in the genome. We explored TAS2R expression in five amphibian species, and found expression in multiple tissues beyond the tongue. Interestingly, species with larger TAS2R repertoires exhibited more expression outside the mouth, which could mean that amphibian TAS2R expansion may have been related to the acquisition of new sensing roles for some genes. Finally, we evaluated whether some TAS2Rs were able to perceive known or suspected bitterants. Most receptors reacted to at least one of the tested substances, and some showed interesting patterns. For instance, a receptor expressed in the skin of toads was able to sense toad toxins, suggesting TAS2R receptors may play roles beyond tasting food in the chemical ecology of amphibians.

## Introduction

The ability of vertebrates to detect the five cardinal tastes—sweet, umami, salty, sour, and bitter—plays a pivotal role in regulating myriad aspects of animal physiology and behavior. Whereas sweet, umami, and mildly salty are generally considered to be attractive stimuli, guiding animals towards nutrient-rich foods, bitter and sour are considered aversive, and are believed to guide animals away from noxious substances, such as toxins or metabolites found in spoiled food [1]. Each taste is generally associated with a suite of related receptors [2]. Bitterness, as perceived by humans, is detected by a family of G protein-coupled receptors (GPCRs) called TAS2Rs or T2Rs, encoded by compact, intronless genes. Previous studies have shown that vertebrates have a wide repertoire of TAS2Rs, which varies considerably across lineages, from zero in cetaceans to over two hundred in frogs [3–5].

Detailed evolutionary, expression, and functional studies have been performed in a variety of species, with a bias towards rodents and primates. TAS2R genes appear to be under relatively rapid birth and death evolution in many vertebrate lineages, with significant differences even between close relatives like rats and mice [6] or humans and other primates [6,7]. In humans and mice, the majority of TAS2Rs are in two or three gene clusters (0.7–30 MB each, containing 10–29 receptors) in the genome with one or two additional singleton genes per genome [8]. There is considerable variation in the presence or absence of distinct TAS2R family members within species, with evidence of rapid pseudogenization and whole gene deletion in chimpanzees [9]. Over the last two decades, ligands have been identified for a wide range of receptors, including humans and mice [10–12], primates [13–17], domestic cats [18], domestic dogs [19], bats [20–23], marsupials and monotremes [24], birds [25,26], fish [27,28], and frogs [3,25]. These studies have shown that some receptors are highly specific, with only one or two known ligands, whereas others respond to a broader suite of chemicals and are considered promiscuous. Many toxic plant secondary metabolites activate one or more human receptors, consistent with bitterness as an oral “early warning” system for foods that may be harmful [29]. However, several recent studies have reported expression of TAS2Rs and other taste pathway proteins in extra-oral tissues like the intestines, brain, airways, and testes. Interruption of these pathways in mice results in reduced parasitic worm clearance [30], altered secretion of the hunger hormone ghrelin [31], and impaired spermatogenesis [32], suggesting extra-oral functions. TAS2Rs are also expressed in mouse adipose tissue, skeletal muscle, and liver [33], although less is known about their function in these tissues. There have only been a handful of studies of extra-oral TAS2Rs in non-mammalian species including trout, cave fish, and chickens. These studies each characterized the expression patterns of a subset of receptors encoded by the genome in a few tissues of interest, and assayed the response of these receptors to classic human bitterants or common feed ingredients [34–36].

Despite evidence that amphibians have an incredible diversity of TAS2Rs, with current literature values ranging from 3 or 4 in Gaboon caecilians (*Geotrypetes seraphini*) to almost 250 in Japanese wrinkled frogs (*Glandirana rugosa*) [4,5,27], the full scope and underlying mechanisms of the massive expansion of TAS2R genes in amphibians, and the sensory and ecological functions of these receptors remain largely unknown. It has been proposed that their diversity may be partly due to the vastly different trophic niches inhabited by tadpoles and adults [4]. Tadpoles display a variety of dietary habits, with most species feeding on plant matter and detritus, while virtually all species of frogs are strictly carnivorous post-metamorphosis [37,38]. Concordantly, Hao et al. found distinct TAS2R expression profiles in the mouth tissue of tadpole and adult American bullfrogs (*Rana catesbeiana*, also known as *Lithobates catesbeianus* or *Aquarana catesbeiana*), and showed that many of these receptors are capable of responding to classic human bitterants [3]. However, most non-anuran amphibians (salamanders and caecilians) are generalist predators throughout their life [39], and amphibians interact with bitter compounds in a variety of ecological contexts beyond aversive taste, suggesting that trophic differences across life stages are not the only factor influencing the evolution and diversification of the TAS2R family. For instance, multiple species across the amphibian phylogeny rely on potentially bitter chemicals, such as alkaloids [40] and cardiac glycosides [41,42], for defense against predators and parasites. In many cases, these chemicals are acquired from prey [43,44] or commensal microbes [45], suggesting that their detection is a key part of chemical defense. Furthermore, as aquatic/semiaquatic species, the ability to detect bitter chemicals in water may play an important role in both behavioral and physiological contexts, such as the selection of microhabitats or egg-laying substrates. Given this, there are many situations in which TAS2Rs expressed in extra-oral tissues can be involved, but the potential physiological function of extra-oral TAS2Rs is poorly understood in amphibians due to the lack of comprehensive expression maps for these extra-oral TAS2Rs.

In this study, we performed genome-wide comparative analyses of 9,291 TAS2Rs from 661 high-quality vertebrate genomes in order to gain insights into the unique evolutionary trajectories and dynamics of the TAS2R gene family in amphibians. We then evaluated functional aspects of TAS2R expansion in amphibians through gene expression analyses of seven tissues across five amphibian species spanning broad evolutionary and ecological diversity. Using *in vitro* receptor functional assays, we assessed ligand profiles of select TAS2Rs against a collection of biologically relevant natural products. The comprehensive findings from this research suggest a distinctive role for TAS2Rs in amphibian ecology and evolution.

## Results

### Distinct evolutionary patterns of TAS2Rs in amphibian genomes compared to other vertebrates

**Fig 1:**
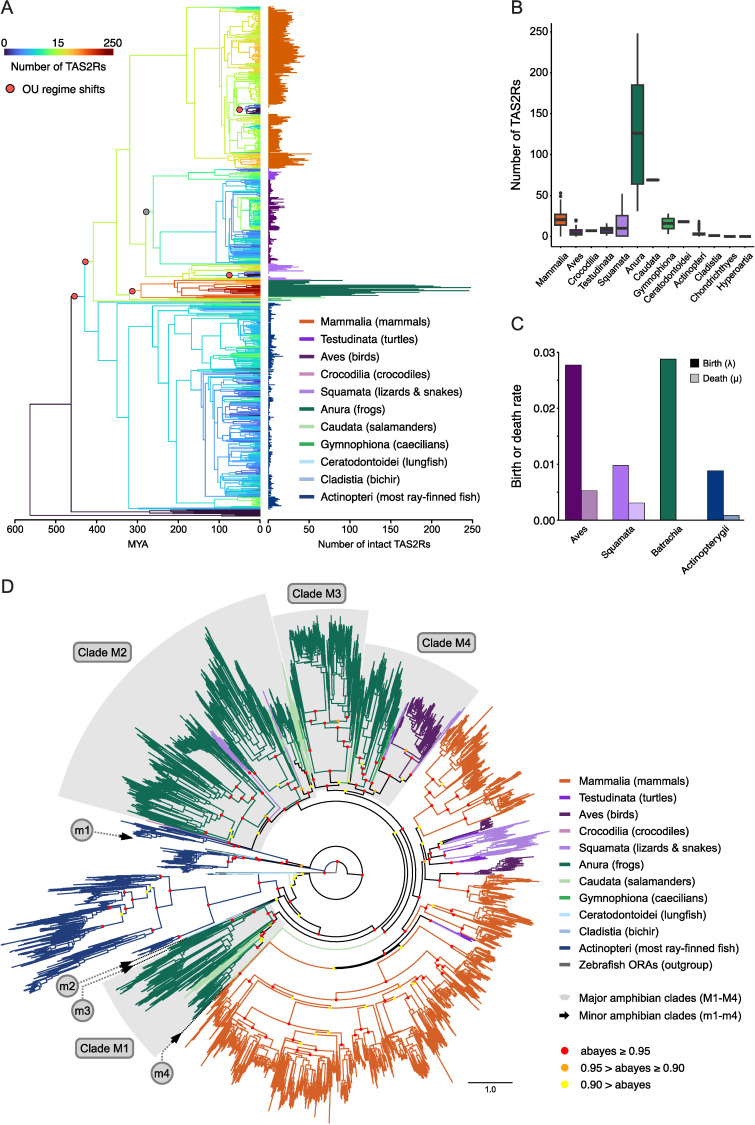
Distinct evolutionary patterns of TAS2Rs in amphibian genomes compared to other vertebrates. (A) Evolution of TAS2R gene content across the 645 vertebrate species examined. Bars adjacent to the phylogeny represent TAS2R counts observed in extant species, and are colored by taxonomic lineage. Tree branches are colored according to DupliPHY ancestral state reconstructions, and were plotted following Revell, 2013 [117]. Note that the color bar is on a logarithmic scale to facilitate visualization. The evolutionary regime shifts from the best-fitting models of continuous traits are labeled as dots on tree branches. The red dots represent the five well-supported shifts considered, and the gray dot represents the sixth shift, which had ambiguous support (see Results section and S3 Fig for details). (B) Boxplots of TAS2Rs content in vertebrate genomes grouped by taxonomic group. (C) CAFE4 birth (λ) and death (μ) rate estimates for the TAS2R family in four vertebrate lineages. (D) Radial phylogenetic tree showing 9,306 TAS2Rs from 681 unique species. The tree also includes 214 published TAS2R sequences which were used to scaffold the alignment, in addition to six zebrafish ORA sequences as outgroups. Branches were colored according to major taxonomic groups, as indicated on the right. Approximate Bayes (aBayes) probabilities are noted as circles on the deep nodes, with red showing confidence greater than 0.95, orange showing confidence 0.90 to 0.95, and yellow showing confidence below 0.90. The tree data with NCBI accessions is shown in S3 Data.

The advent of advanced sequencing technologies and the surge in high-quality genome sequences throughout the animal kingdom in recent years have opened new avenues for taxonomically broad genome mining for diverse gene families, such as TAS2Rs. We developed a computational pipeline to identify bitter taste receptors in any unannotated genome, and used it to analyze chromosome-level assemblies from 661 vertebrate species, identifying 9,291 intact TAS2Rs. The number of genes per species ranged from 0 in jawless fish, cartilaginous fish, and cetaceans, to 248 in the wood frog (*Lithobates sylvaticus*) (Fig 1A and 1B). This result is generally consistent with a recent comprehensive evolutionary study of vertebrate chemoreceptor genes [5] despite some differences, such as missing cartilaginous fish receptors in our analysis (S1 Fig).

We constructed a phylogenetic gene tree by maximum-likelihood containing the 9,291 TAS2Rs found in our chosen species (Fig 1D). We noticed that TAS2Rs diverged extensively after the major vertebrate lineages were established. Most amphibian receptors are found in four major groups (clade M1-M4). Several receptors form small groups in the gene tree (clade m1- m4). All the major clades have receptors from frogs, salamanders, and caecilians, whereas caecilian receptors are relatively accumulated in the clade M4. The minor “clade m1” containing a small number of salamander and caecilian receptors are clustered with lungfish receptors (approximate bayes > 0.95) and close to actinopteri receptors (0.95 > aBayes > 0.90). We do find that bird and reptile (Sauropsida) receptors are relatively distributed, with some grouping with amphibians and others with mammals.

To gain a general view of the evolutionary dynamics of TAS2R gene content in vertebrates, we reconstructed ancestral TAS2R family sizes across our focal species’ phylogeny using DupliPHY [46]. Our reconstruction revealed a rapid increase in the number of receptors along the branch ancestral to all batrachians (i.e., frogs and salamanders). This clade contained by far the largest TAS2R content among vertebrates (median of 104 across species), as opposed to caecilians, their sister group, which displayed numbers of TAS2Rs comparable with other vertebrates (3–16 genes per species). Furthermore, we found two marked declines in TAS2R family size in cetaceans and snakes, consistent with previous work suggesting a general reduction in taste receptors in these lineages’ genomes, possibly associated with aquatic and fossorial lifestyles, foraging patterns, and diets [5,47–50].

Even though amphibians have a wide variety of genome sizes and tend to have considerably larger genomes than other tetrapods [51,52], the increased number of TAS2Rs in amphibians does not seem to be associated with a bulk increase in genetic material, such as that caused by whole-genome duplications or chromosomal duplications. A set of 236 species in our database have published genome size estimates [51]. We found no effect of genome size on TAS2R count neither across vertebrates in our dataset (phylogenetic generalized least squares regression [pgls]; β = 0.231, t = 1.60, p = 0.11, adj. r^2^ = 0.01; S2 Fig), nor when considering amphibians alone (pgls; β = 0.131, t = 0.429, p = 0.67, adj. r^2^ = −0.07). An example of rapid genome expansion within our data further supports this conclusion. The *Xenopus laevis* and *X. borealis* lineage has undergone an allotetraploidy event, in which the genomes of two diploid (2n) species fused to form a tetraploid (4n) species [53]. However, *X. laevis* and *X. borealis* and their relative, *X. tropicalis*, which retains the ancestral diploid karyotype, all have exactly 50 unique TAS2R sequences. The number of TAS2R genes remained stable despite a whole-genome duplication, possibly due to post-duplication gene loss. This suggests that the expansion of TAS2R genes in amphibians is not merely a byproduct of large-scale genomic duplications, but rather influenced by other mechanisms.

To further investigate the processes through which TAS2R gene content differences among vertebrate lineages have evolved, we estimated gene duplication and loss rates for batrachians, birds, squamates, and ray-finned fishes by parameterizing a birth-death model of gene family evolution using our TAS2R family size data [54,55]. Batrachians had the highest birth rate (λ = 0.0288), followed by birds (λ = 0.0277), while squamates and ray-finned fishes displayed much lower values for this parameter (λ = 0.0097 and 0.0088, respectively). The death rate for batrachians was considerably lower than those of other lineages (µ = 0.00002), which exhibited rates 1–2 orders of magnitude higher (birds: µ = 0.0052, squamates: µ = 0.0031, ray-finned fishes: µ = 0.00081; Fig 1C). Taken together, these findings suggest that the rapid accumulation of TAS2Rs genes in batrachian genomes was mediated by both an increase in gene duplication rate and, especially, a decrease in the rate of gene loss (i.e., a higher gene retention rate).

Next, we evaluated the degree to which positive and stabilizing selection may have influenced the evolution of TAS2R gene content, using a toolkit of continuous-trait evolution models. Briefly our approach consisted of evaluating the fit of Brownian Motion (BM) [56,57] and Ornstein-Uhlenbeck (OU) [58,59] models to describe the evolution of TAS2R family size across vertebrates. These models describe the change of a quantitative trait over time on a phylogeny. Under BM, a lineage’s character state “walks” randomly over phenotype space, and is equally likely to move in any direction at any given time as it evolves. Under OU dynamics, trait evolution is also modeled as a random walk over time, but with the added presence of one or more “optimal” trait values, meant to represent selective optima, towards which traits are more likely to evolve. If a lineage’s trait value is far from the optimum, the lineage is likely to evolve towards it via positive selection, while lineages close to the optimum will remain in its vicinity due to stabilizing selection. Both model types (BM and OU) can be parametrized so different subtrees of a phylogeny experience different evolutionary regimes by assigning them different optima (OU) or mean traits and variances (BM).

We first assessed whether our data showed evidence for multiple evolutionary regimes by fitting and ranking a wide range single and multi-regime OU models to our data using the R package *l1ou* [60]. The two best models, which accounted for 81% of the pBIC weight identified six highly-supported regime shifts across the vertebrate phylogeny (marked as red dots on Fig 1A): Three increases in the “optimal” gene family size were inferred at the base of bony fishes, the branch ancestral to lobe-finned fishes, and the branch ancestral to batrachians, and three decreases were inferred in cetaceans, snakes, and the warbler *Setophaga coronata*, most of which exhibited zero or one receptor (Figs 1A and S3). An additional decrease along the branch ancestral to birds, crocodilians, and testudines (gray dot on Fig 1A) was present in the best model, but an identical model without this shift had only slightly lower support (ΔpBIC = 0.43, models 1 and 2 in S3 Fig). S3 Fig displays the four best shift configurations, accounting for ~97% of the pBIC weight.

Based on the evolutionary regime shifts identified above, we compared the fit of their corresponding OU models with comparable multi-regime BM models to gain insight on the extent to which selection has influenced the evolution of TAS2R family size. Being composed of a single species, we excluded the *S. coronata* shift for model simplicity. Qualitatively identical results were obtained including this shift, but the fitting of some models was less robust. The OU models with five and six regime shifts (see dots in Fig 1A) fit the data unambiguously better than either of their homologous BM models, as well as single-peak BM and OU models (combined AIC weight > 99%; Table 1), supporting a role for selection in the evolution of TAS2R gene content in vertebrates.

**Table 1:** Model-fitting results for six different models of continuous trait evolution. Evolutionary regime shifts are labeled on Fig 1A. Five-shift models correspond to the red dots, while six-shift models also include a shift in the gray dot.

Model	AIC	ΔAIC	AIC weight
OU 6-shift	742.91	0.00	0.99997
OU 5-shift	764.10	21.19	0.00003
BM 6-shift	805.93	63.02	0.00000
BM 5-shift	837.39	94.48	0.00000
OU single	855.20	112.29	0.00000
BM single	865.20	122.29	0.00000

Overall, these results suggest that the TAS2R repertoire may be evolving under different regimes in different groups of vertebrates, with amphibians exhibiting markedly different dynamics from other groups. Further, they are consistent with the idea that these evolutionary regimes are, at least in part, caused by different adaptive optima, towards which each lineage has evolved, with batrachians inhabiting an “adaptive zone” where a higher number of genes is advantageous.

### Tandem organization of TAS2R gene families promotes rapid copy number evolution

In order to shed light on the proximal causes of TAS2R accumulation in batrachians, we considered several possible mechanisms that could cause increased non-allelic homologous recombination in the region surrounding TAS2Rs, and thus alter gene duplication, loss, and conversion rates. We considered whether each gene was alone or clustered near additional TAS2Rs, the location of the gene along the chromosome, and the proximity of each gene to repetitive elements.

**Fig 2:**
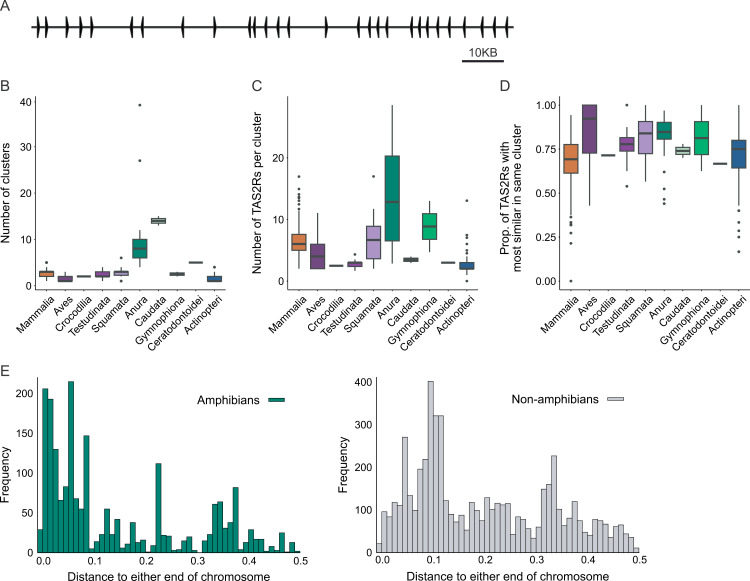
Tandem organization of TAS2R gene families promotes rapid copy number evolution. (A) Diagram of a western clawed frog gene cluster spanning 160 KB and containing 28 TAS2Rs found on chromosome 9 (aka CM004451.2 or NC_030685.2). (B) Boxplot showing clusters per genome. Only species that have TAS2Rs are included. (C) Boxplot showing TAS2Rs per cluster. Only species that have clusters are included. (D) Boxplot showing proportion of TAS2Rs with the most similar gene in the same cluster. Only species that have clusters are included. (E) Chromosomal location of TAS2Rs for amphibian (left) and non-amphibian (right) genes. Along the x-axis, 0.0 represents either end and 0.5 is the numerical center of the chromosome. Alternative representations colored by genome assembly qualities (i.e., BUSCO gene completeness and contig N50) or sorted by the several parameters of genome assembly qualities are shown in S6 Fig.

Prior studies have reported that TAS2Rs are located in clusters in humans, mice, and a few frogs [4,8]. Since nonallelic homologous recombination is elevated among clustered genes [61,62], this seemed to be a plausible candidate mechanism. We classified two genes as clustered when their start sites are within a 1 megabase window, but our analyses are robust to the choice of window size, as well as alternative clustering strategies (S4 Fig). By this definition, TAS2R clusters exist in 62% of species, or 76% of species with two or more genes, and around 82% of genes found across species are clustered. An example cluster found in chromosome 9 of *X. tropicalis* (aka CM004451.2 or NC_030685.2) is shown in Fig 2A. Salamanders and frogs (i.e., batrachians) tend to have more clusters than other species, with up to 39 in the Puerto Rican coqui (*Eleutherodactylus coqui*, Fig 2B), and the average number of genes within each cluster is also elevated in this group (Fig 2C). Concordantly, among species where we found TAS2R gene clusters, both the number of clusters and average genes per cluster showed strong positive associations with TAS2R count across vertebrates (pgls; number of clusters: β = 1.1, t = 50.7 p <2e-16; avg. genes per cluster: β = 0.88, t = 51.04 p <2e-16; full model adj. r^2^ = 0.92), as well as within frogs and salamanders (pgls; number of clusters: β = 1.2, t = 40.8 p <2e-16; avg. genes per cluster: β = 0.97, t = 39.8 p <2e-16; full model adj. r^2^ = 0.98). Overall, these results indicate that both the addition of genes to existing clusters and the creation of new clusters have been important contributors to the expansion of the TAS2R gene family.

Some lineages, however, showed different patterns. In Squamata we find a much more modest elevation of cluster number than in batrachians, resulting in a very high genes-per cluster ratio, suggesting that in this clade expansion of existing clusters may be more significant than formation of new clusters. Conversely, in ray-finned fishes and birds, the number of genes per cluster is small, suggesting formation of new clusters or, at least in the case of ray-finned fishes, low rates of tandem duplication. Amphibians do not significantly differ from other vertebrates in terms of the fraction of genes that are in clusters or the intergenic spacing within a cluster (measured as kb of cluster per gene, S5 Fig). We also identified the most similar other gene for each receptor and calculated the proportion of closest gene pairs in the same cluster, similar to what was done with human and mouse genomes by Conte et al. [8]. These numbers are high, with a mean of 73% among species that have clusters (Fig 2D). This suggests either a new cluster or gene conversion maintaining similarity within each cluster. Amphibians again showed similar variation to other vertebrate species with at least one cluster.

Earlier studies have shown that for certain chromosomes, recombination is repressed near the centromere and enhanced near the telomeres [63], and that in at least 3 frog genomes, TAS2Rs preferentially localize to the ends of chromosomes [4]. In our study, TAS2R genes are generally closer to the ends of the chromosomes in the 24 studied species of amphibians, than in non-amphibian species (Figs 2E and S6). The distance from each gene to the nearest end of the chromosome (0.0 is either end, 0.5 is the numerical center), was smaller in amphibians (amphibians: mean = 0.148, s.d. = 0.14; others: mean = 0.202, s.d. = 0.13; Welch’s two-sample t-test t = −15.25, d.f. = 3587.5, p = 3.2e-51, two-sample Kolmogorov-Smirnoff test, two-sided: D = 0.32963, p = 0). Importantly, we find that clustered genes are located closer to the telomeres than singleton genes (mean 0.18 vs. 0.24, Welch’s two-sample t-test t = 14.997, df = 2368.2, p <1.2e-48). This supports our hypothesis that genes located closer to the telomeres have a higher chance of being duplicated.

Finally, neighboring repeat elements represent a possible mechanism for TAS2R duplication and deletion through either retrotransposition or promotion of non-allelic homologous recombination [64,65]. One study has found enrichment of repeat elements near to TAS2Rs in the coelacanth, which is thought to have undergone a TAS2R expansion [66]. To test whether the same mechanism applies to batrachians, we searched for repeat elements in a subset of species, including most of our amphibians (17) and a comparable number of randomly selected non-amphibians (18). Our analysis includes total repeat elements, DNA elements, LTR elements, short and long interspersed retrotransposable elements (SINEs and LINEs) (S7 Fig and S1 and S2 Tables). We found that amphibians have larger percentages of repeat elements than other species (mean of 54.9% vs 32.1%, one-sided t-test with unequal variance: p = 0.00065), but this does not seem to correlate with enrichment near TAS2Rs. For non-amphibian species, the region near TAS2Rs has significant enrichments of LINEs (p = 0.0073) and loss of SINES (p = 0.049). Since LINES are associated with low-recombination rates [67] while SINES enhance recombination [68], this may provide a mechanism slowing recombination for non-amphibian clusters. However, there was no support for our hypothesis that repeat elements are preferentially promoting TAS2R recombination or retrotransposition in amphibians. These findings suggest that the frequent clustering of TAS2R genes and the proximity of TAS2R gene clusters to the telomeres compared to non-amphibians might have promoted rapid expansion of TAS2R repertoire by tandem duplication in amphibians.

### Varying levels of turnover across the TAS2R family

Our TAS2R gene tree displayed an intriguing feature: The majority of receptors were clustered in groups composed of only receptors from the same vertebrate lineage (Fig 1D), suggesting a low level of orthology between TAS2R receptors in different vertebrate clades. This pattern could be explained by the high duplication rates observed in some lineages (e.g., batrachians and birds), but considering the relatively homogenous family sizes across most lineages, we would expect similarly high loss rates, which we did not find (see Fig 1C). To further inquire into the mechanisms leading to the observed phylogenetic relationships between TAS2R genes, we performed species trio comparisons, where we inspected the relationships between TAS2R pairs of species plus an outgroup to determine the orthology relationships between genes based their phylogenetic relationships. We compared 18 species pairs (6 amphibians, 6 mammals, 6 birds) with divergence times between 0.28–120 million years.

**Fig 3:**
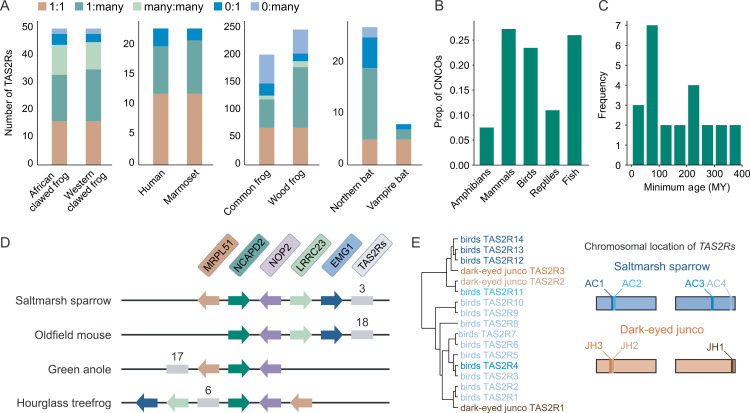
Despite rapid turnover of TAS2R genes, TAS2R clusters are deeply conserved. (A) Comparison between TAS2R repertoires of closely related species, based on the tree in Fig 1B. First panel shows the comparison between the African clawed frog and western clawed frog, which diverged 58 MYA. The outgroup is the Congo frog, which has 45 TAS2Rs. The second panel shows the wood frog and common frog (divergence 33 MYA) with the outgroup of the pixie frog (98 genes). The third shows the human and the white-tufted-ear marmoset (divergence 43 MYA) with the outgroup of the slow loris (22 genes). The final panel shows the vampire and northern bats (divergence 51 MYA) with the outgroup of the fruit bat (15 genes). Additional comparisons are available in S8 Fig. (B) Plot of fraction of all genes that are CNCOs, meaning that they have 2+ copies in fewer than 5% of species represented by this region of the species tree, and exactly one copy in over 50% of species. To control for varying gene family sizes, we display the data in terms of individual genes (with a gene family often containing multiple genes in the same species). Note that “reptiles” excludes birds, and “fish” only includes actinopterygii. (C) Minimum age estimates for a subset of clusters, with orthology of clusters defined by conserved neighboring BUSCO genes. (D) Schematic illustrating a specific conserved cluster found in members of all major tetrapod lineages, making it at least 352 MY old. Conserved BUSCO marker genes shown as colored arrows, with arrowhead conveying directionality. TAS2R cluster shown as gray line with number of included genes above it. (E) Subset of Fig 2 focusing on the saltmarsh sparrow and dark-eyed junco on the left. Coloring reflects locus identity, matching chromosomal diagram on right. In the chromosomal diagram, each TAS2R locus is shown as a bar in the approximate location of this TAS2R singleton or cluster. In the case of immediately adjacent clusters (i.e., AC1/AC2 and JH2/JH3), the distance between them has been exaggerated slightly to resolve the separate loci.

In the majority of comparisons (13/18), we found comparable numbers of TAS2R genes between species, yet in many cases less than 50% of genes displayed one-to-one orthology, while one-to-many, many-to-many, and many-to-zero relationships were more common (Figs 3A and S8). This pattern was more prevalent in amphibians, and became more pronounced in species pairs with older divergence times (S8 Fig). This is, again, consistent with a scenario of very rapid gene turnover, which contrasts with the low loss rate found across species, and especially in amphibians.

This discordance can be explained in two complementary ways: First, since the birth-death model used does not take orthology into account, inferences of duplication and loss rates can result in underestimation, since gene gains and losses that occur along the same branch may cancel each other out in the eyes of the model. This being said, maximum likelihood estimation of these parameters (as opposed to count/parsimony methods) can, to an extent, ameliorate this caveat, at least in some cases [69,70]. Alternatively, if gene conversion is frequent, then neighboring paralogous genes may become homogenized, which would “erase” the phylogenetic signature of orthology, leading to an overestimation of one-to-many and many-to-many orthologous relationships. In reality, both of the above explanations probably contribute to our observations to some extent. However, considering their high degree of clustering and the positions of regions with higher recombination probabilities, we suspect these results may be due to an important extent to high degrees of non-allelic gene conversion between closely related genes. Consequently, we propose this process as an important force shaping the evolution of TAS2R genes in amphibians, and possibly in other vertebrate groups.

Interestingly, we found certain genes whose copy number appeared much more constrained than the rest of TAS2Rs. Across vertebrates, 6–26% of genes were found to exist as single copies in at least 50% of species, while at most 5% of species had two or more copies. We dubbed these genes “copy-number-constrained orthologs (CNCOs).” CNCOs were uncommon in all lineages, but represent a significantly higher percentage in birds, mammals, and ray-finned fish than in amphibians and reptiles (Fig 3B. Welch’s two-sample t-test: t = −7.9195, df = 1.8431, p = 0.0196). Furthermore, singleton genes (i.e., those not in TAS2R gene clusters) were proportionally more likely to be CNCOs in all orders except in reptiles (S9 Fig).

Given that TAS2R clusters occur in regions with putatively higher rates of recombination, we are not surprised to find many singleton CNCOs, or that cluster-rich clades have fewer of these genes. This said, we found an interesting pattern of CNCOs occurring within clusters. In amphibians, clusters containing CNCOs, were more likely to have more than one CNCO than would be predicted by chance, given the distribution of amphibian cluster sizes (binomial distribution predicts P = 0.22, actual P = 0.63, two-proportion z-test Z score 4.98, p = 6.3e-7). The same trend appears for mammalian clusters, but is not significant (theoretical P = 0.47, actual P = 0.53, Z score 1.70, p = 0.089). This could be due to CNCO-rich clusters occurring in regions of low recombination, and perhaps having formed slowly, or during a prior period of higher recombination. It is also possible that duplication of CNCOs is somehow deleterious, which may promote reduced recombination rates in their vicinity.

Although many TAS2R genes appear to turnover quickly, we hypothesized that some clusters might be older than the genes within them. We identified orthologous TAS2R loci across species by examining flanking BUSCO genes (S3 Table), and used the age of the most recent common ancestor of all species containing a locus as a lower bound on its age (Fig 3C). We find evidence that at least two loci are very old, including members of multiple orders. Notably, we found gene clusters surrounded by the same set of five BUSCOs in all four major tetrapod lineages, suggesting that these genes represent a homologous cluster that has existed for at least 350 million years (Fig 3C and 3D). Interestingly, the three saltmarsh sparrow genes in cluster 6 are 78–93% identical at the amino acid level, suggesting that they either diverged recently, have experienced consistent purifying selection for a long time, or became homogenized by gene conversion [71].

Using this same approach, we were also able to quantify the degree of conservation between homologous loci that may have otherwise been overlooked, and again found a variety of patterns. For instance, singleton (unclustered) genes from the sablefish (*Anoplopoma fimbria*) and mangrove rivulus (*Kryptolebias marmoratus*) that are only 42% identical at the amino acid level are flanked by the same seven BUSCO genes (S10 Fig), indicating a much faster rate of evolution than, for instance, genes in the 350 MY-old cluster mentioned above. Conversely, we also find evidence for recent turnover in a small number of clusters. For instance, the dark-eyed junco (*Junco hyemalis)* lacks a cluster orthologous to the saltmarsh sparrow’s AC3 (*Ammodramus caudacutus)*, suggesting that this cluster was lost within the last 8.8 million years (Fig 3E). Likewise, we found a cluster, present in all members of the genus *Rana* (RT4 in S11 Fig) but not in the closely related genus *Lithobates* (or any other lineage), suggesting it arose in the past 20.1-33 MY.

Overall, the varying levels of turnover across different gene lineages of the TAS2R family suggest multiple different mechanisms are driving the evolution of the bitter taste receptor repertoire in vertebrates. The phylogenetic relationships and genomic location of these genes suggest that recombination may play an important role in creating variation in the TAS2R gene repertoire through changes in both copy number and gene conversion. A consistent input of such variation could then use other evolutionary forces [72], namely selection and drift, to drive the dynamic evolution of TAS2R repertoire observed across vertebrates.

### TAS2R amplification in batrachians is accompanied by extra-oral-specific utilization

**Fig 4:**
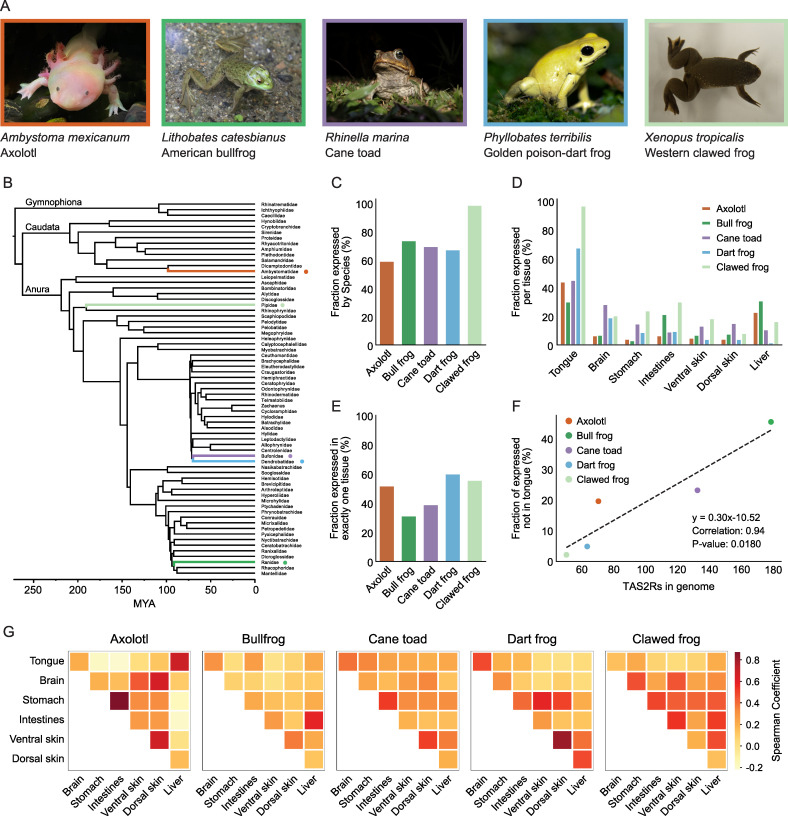
TAS2R amplification in batrachians is accompanied by extra-oral-specific utilization. (A) Photos and latin names for species included in transcriptomic analysis. Photos of the *Rhinella marina* and *Phyllobates terribilis* were obtained from Brian Gratwicke under CC BY 2.0. The photos of *Ambystoma mexicanum* and *Lithobates catesbianus* were taken by Jing-Ke Weng (author), and the photo of *Xenopus tropicalis* was taken by Akihiro Itoigawa (author). (B) Tree of major amphibian families, with amphibians included in this study shown in colors matching boxes in Fig 4A. Topology and divergence times follow Pyron [118]. (C) Percent of receptors in genome that are expressed in any sequenced tissue. (D) Percent of receptors in genome that are expressed in specific tissues. (E) Percent of expressed receptors that are expressed in exactly one tissue. (F) Correlation between number of receptors in genome and number of receptors that are expressed in at least one extra-oral tissue (but not the tongue). (G) Colocalization matrices showing Spearman coefficients for TAS2R overlap between pairs of amphibian tissues in five different amphibian species.

To characterize the function of a vast number of TAS2Rs in amphibians, we assessed the tissue-specific expression patterns of TAS2R genes in four frogs and one salamander species (Fig 4A): the cane toad (*Rhinella marina*), the golden poison-dart frog (*Phyllobates terribilis*), the American bullfrog (*Ra. catesbaiana*), the tropical clawed frog (*X. tropicalis*), and the axolotl (*Ambystoma mexicanum*). These species encompass a wide range of the phylogenetic and ecological diversity of amphibians, and have sufficiently developed genomic resources for the purposes of this study (Fig 4B). For instance, *Rh. marina* and *P. terribilis* secrete defensive toxins that are biosynthesized or sequestered from dietary items respectively. Their diets range from aquatic micro animals (*X. tropicalis*) and molluscs (*A. mexicanum*) to leaf-litter insects (e.g., ants; *P. terribilis*) and marsh animals (e.g., small mammals, crayfish, other frogs; *Ra. catesbaiana*). We also find a wide range of TAS2Rs in these species using our genome-mining method, from 50 in the clawed frog up to 178 in the bullfrog, with the golden poison-dart frog (63), axolotl (70), and the cane toad (132) in between. Based on prior results in humans, mice, chickens, and fish [30,31,33–35], we quantified TAS2R gene expression in the tongue, brain, stomach, intestines, and liver. Moreover, since the amphibian skin is known to have an important chemosensory function, which often varies between different regions of the skin [73], we also separately analyzed dorsal and ventral skin samples. We focused on post-metamorphic tissues since the only life stages study performed so far found similar ligand receptivity profiles between tadpoles and adult frogs [3].

For all amphibians, more than half of TAS2R repertoire were detected in any of seven tissue transcriptomes (Fig 4C). In most amphibians, the tongue has the greatest diversity of TAS2Rs, but only a fraction of the TAS2Rs in the genome were expressed (Fig 4D). In cane toads, for instance, we found expression of 45% of TAS2Rs (defined as FPKM > 0.01) in the tongue, compared to 11% in the liver. Similar patterns are seen in axolotl and dart frogs (Fig 4D). The bullfrog is unusual in that the proportion expressed in the liver (31%) is very similar to the proportion expressed in the tongue (30%). However, we find that many of these receptors are found only in bullfrog liver replicate 3. This one sample contains 73 TAS2Rs, as compared to 16 and 31 in the other two replicates (S12 Fig). The clawed frog is unique in that the vast majority of receptors are expressed in at least one tissue, almost always including the tongue. This result is particularly pronounced when we normalize expressed receptors by the number of TAS2Rs in the genome (which is smaller for clawed frogs than the other species, see Fig 4C). 98% of clawed frog receptors are expressed in at least one replicate of at least one tissue, similar to what is seen in mice and humans [11,74]. In contrast, only 59–73% of receptors are expressed in the tongue in other amphibian species. When examining the clawed frog data at a tissue level (Fig 4D), we find that the vast majority of TAS2Rs are expressed in the tongue and often at high levels. 95% of receptors were detected in the tongue, as compared to 8–29% in extra-oral tissues.

We were also interested in determining the extent to which receptors function across multiple tissues. To address this question, we calculated the percentage of expressed genes that are unique to exactly one tissue in each species (Fig 4E). Bullfrog and cane toad have low values (31% and 39%) compared to axolotl, dart frogs, and clawed frogs (51%, 60%, 55%). Importantly, the vast majority of these unique receptors are localized to the tongue (S13 Fig). However, many more receptors occur in multiple extra-oral tissues. When calculating the percent that are expressed in extra-oral tissues but not in the tongue, clawed and dart are low (2.0%, 4.8%), axolotl and cane toad are intermediate (20%, 23%), and bullfrog is high (45%). These numbers are directly proportional to the number of TAS2Rs in the genome (Pearson’s r = 0.94, p = 0.018, see Fig 4F), suggesting that the species with more TAS2Rs in their genome may have expanded extra-oral-specific utilization.

Finally, we wanted to use our data to explore the similarity of TAS2R profiles between tissues, with the hypothesis that developmentally related tissues would have similar profiles. When we apply a hierarchical clustering algorithm to our TAS2R expression table, we find some degree of clustering by germ layer (S14 Fig). Brain and skin are both ectodermal [75], and they are grouped together in the dendrogram. Stomach, intestines, and liver are all endodermal [75] and are near each other on the dendrogram, but not on a single branch. Although the tongue is derived from multiple lineages, amphibian taste buds come from the endoderm and the fungiform papillae come from the ectoderm [76]. Both of these tissue types have been shown to have TAS2R expression in humans [74,77]. We find that tongue tissues separate from all other lineages in our clustering algorithm.

To further test the correlation between tissues, we created a Spearman correlation matrix (Fig 4G) for each species. The most consistently high comparison is dorsal and ventral skin (ρ between 0.20 and 0.81). The stomach and intestines comparison is also generally high (ρ between 0.22 and 0.87). Otherwise, there is very little agreement between species. For instance, the axolotl tongue and liver have high correlations (ρ = 0.68), but this value is low in all other species (ρ between 0.004 and 0.23). We do not find any consistent relationship between skin and brain, the ectodermal tissues.

**Fig 5:**
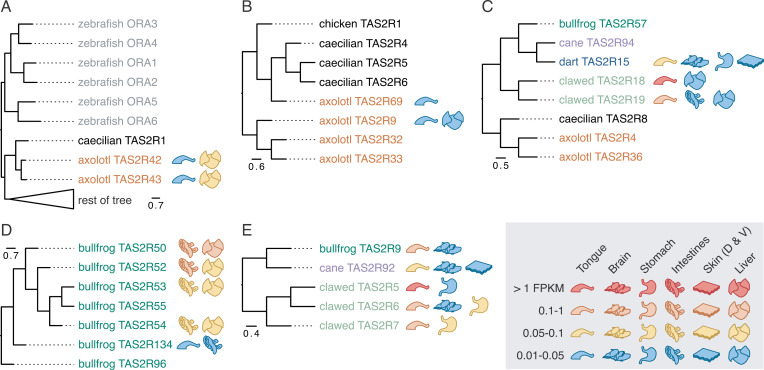
Phylogenetically related genes often have similar patterns of expression. All trees in this figure were created from S4 Data, which is shown in S15 Fig. Branch lengths represent phylogenetic distance, with scale shown below each subtree. Coloring represents different species. Icons represent tissue expression as shown in legend. (A) Root of tree highlighting relationship between axolotl TAS2R42 and TAS2R43. Triangle represents the collapse of the rest of the tree. (B) Subset of tree showing single chicken receptor clustering with amphibian receptors. (C) Subset of tree showing a rare example of orthology between all five batrachian species. (D) Subset of tree showing bullfrog-specific radiation with intestinal and liver expression. (E) Subset of tree showing clade containing bullfrog, cane, and dart receptors expressed in the tongue and brain (among others).

Next, we reviewed the relationship between sequence similarity and expression pattern. We used our expression data to annotate a phylogenetic tree containing sequences from all five amphibian species. The final tree is shown in the S15 Fig, with several key panels shown in Fig 5. Consistent with the large phylogenetic tree in Fig 1D, we note that amphibian sequences appear to diverge quickly, with large species-specific radiations in this tree. Amphibian species appear in three broad clades. As shown in Fig 5A, axolotl receptors TAS2R42 and 43 group with caecilian TAS2R1, and this group diverges from other receptors very early on, close to the root, similar to clade m1 from Fig 1D. Both of the axolotl genes are expressed in the tongue and liver. A high confidence node (aBayes > 0.95) separates these sequences from the vomeronasal receptor (ORA) outgroup sequences. The second clade and third clade include sequences from all five amphibian species, with all mammalian and bird sequences appearing in the third clade. Indeed, all of these non-amphibian receptors appear together, with the exception of one chicken receptor (chicken TAS2R1) which is part of an amphibian clade (Fig 5B), potentially reflecting the small avian clade that grouped with amphibians in Fig 1D. Even when comparing closely related species (ex: the golden poison dart frog and cane toad are closely related, as shown in Fig 4A), we find few orthologs between species. There is only one instance of perfectly discernible orthology across all five amphibian species, as shown in Fig 5C. Note that many of these receptors are expressed in the tongue. In other parts of the tree, we notice that similar sequences have similar patterns of expression. For instance, there is an intestines/liver-specific, bullfrog-specific radiation in Fig 5D and a group of tongue/brain-expressed receptors from the bullfrog, cane toad, and clawed frog in Fig 5E.

Our RNA-sequencing data illustrate several important points. First, we find that the clawed frog is similar to previously-sequenced vertebrates in that the majority of TAS2Rs are expressed in the tongue, with some receptors additionally expressed extra-orally. In contrast, many other amphibians seem to have developed extra-oral specific receptors. Indeed, the expanded TAS2R repertoire of these species correlates with non-orally expressed receptors. Finally, we find that phylogenetically similar receptors often have similar patterns of expression, both between paralogs within species-specific radiations and across orthologs between species. These suggest that the large expansion of the TAS2R repertoire may lead to diversification of expression patterns, contributing to the exclusive use of a large number of TAS2Rs in non-oral organs—a phenomenon uncommon in other vertebrates.

### Extra-oral receptors sensitive to ligands that act in extra-oral tissues

**Fig 6:**
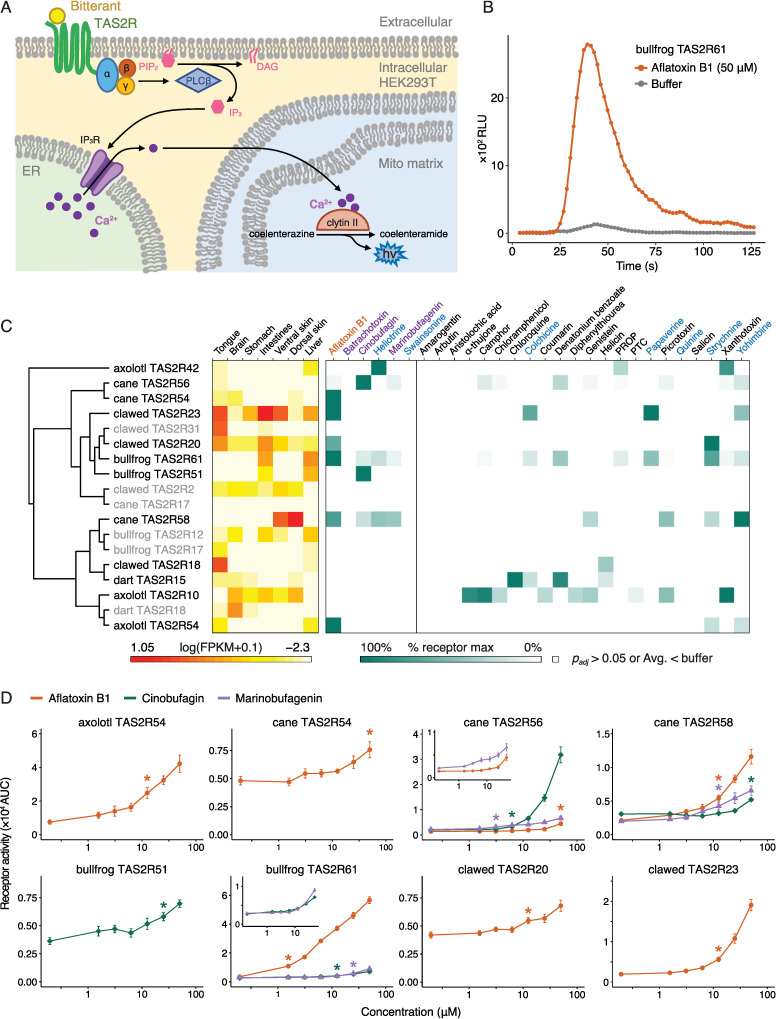
Extra-oral receptors sensitive to ligands that act in extra-oral tissues. (A) schematic illustrating our *in vitro* functional assay inside a human embryonic kidney (HEK293T) cell. Activation of the TAS2R by an extracellular bitterant causes calcium release from the endoplasmic reticulum, which reacts with coelenterazine and clytin-II in the mitochondrial matrix to produce blue light. (B) Raw readout of luminescence during a positive bitter assay, with bullfrog TAS2R61 as an example. The orange curve shows the data for aflatoxin B1 and the gray curve is for the buffer condition. For clarity, only one replicate is shown for each condition. (C) Diagram showing phylogenetic tree, heatmap for expression data, and heatmap for receptor activity. Phylogenetic tree is subset from the large tree in S15 Fig. Receptors not responding to any tested substances in our functional assay were represented in gray. Expression data are measured in fragments per kilobase of transcript per million mapped reads (FPKM) and are shown on a log scale, with 0.1 added to allow for FPKM = 0 values. Functional assay data are white if not significantly higher than buffer (two-sided Welch’s t-tests corrected by Benjamini-Hochberg adjustment with α = 0.05). Significant data are normalized to the largest chemical to buffer difference for each receptor, with 100% of receptor maximum shown in dark green with a linear scale down to a 0% difference in white. Chemicals are split into ecologically relevant substances (left) and classic bitterants (right), and then alphabetized within each group. Fungal toxins, frogs’ toxins, and natural alkaloids are represented in orange, purple, and light blue, respectively. (D) Dose response relationships for eight important receptor/ligand pairs (n = 6–9, mean ± SEM). Lines are represented in orange for aflatoxin B1, green for cinobufagin, and purple for marinobufagenin. Insets show the magnifying displays for a subset of ligands. Asterisks indicate minimum concentrations where T2R-transfected cells showed responses significantly higher than those in the lowest concentration (*p* < 0.05, Dunnett’s tests).

Based on the annotated tree, we chose 18 receptors for follow-up functional characterization in a luminescence-based assay (Fig 6A and 6B). We selected receptors to represent the broad range of amphibian TAS2Rs, exemplifying different patterns of copy number evolution and gene expression as shown in S1 Data. We tested 18 chosen amphibian receptors against a panel of 28 substances (S4 Table). The panel includes 6 natural products potentially relevant to amphibian ecology and 22 classic bitterants with diverse chemical structures. Marinobufagenin and cinobufagin are the cardiotoxic steroids that are produced by cane toads and Asian toads, respectively, along with many of their lesser-studied relatives. Batrachotoxin is found to be present in the skin of golden poison-dart frogs, and is sequestered from dietary sources [78]. Aflatoxin B1, heliotrine, and swainsonine are all plant- or fungus-derived toxins that might be ingested by insects, which are, in turn, eaten by amphibians.

We identified agonists for twelve of the eighteen amphibian TAS2Rs, including 3 axolotl, 2 bullfrog, 3 cane toad, 1 dart frog, and 3 clawed frog TAS2Rs. Our results are summarized alongside expression data in Fig 6C, with additional results in S16 Fig. For key receptors, dose response relationships are shown in Fig 6D. As has been observed in other vertebrates, some receptors responded to a variety of substances while others were specific to only one or two compounds. For instance, bullfrog TAS2R61 responded to 14 compounds including natural alkaloids and bufotoxins. In contrast, bullfrog TAS2R51 responded only to cinobufagin. A pair of the phylogenetically related receptors, dart TAS2R15 and clawed TAS2R18 (Fig 5C), had the same agonist, helicin, whereas the other major agonists were not shared.

Eight of the twelve deorphanized receptors, with representatives from all five sampled species, are expressed in the tongue and sensitive to classic bitterants and/or ecologically relevant chemicals. This suggests that many amphibians can detect both classic bitterants and chemicals that they potentially encounter in their environments as bitterness. On the other hand, certain chemicals were recognized by multiple extra-oral receptors in different species, sometimes at the site of the chemical’s known biological action. Four of the five species can detect the hepatotoxin aflatoxin B1 (axolotl TAS2R54, bullfrog TAS2R61, cane TAS2R54/56/58, and clawed TAS2R20/23) in a dose-response manner (Fig 6D). It is noted that axolotl TAS2R54, bullfrog TAS2R61, and clawed TAS2R20/23 are all expressed in the liver (Fig 6C).

Bufadienolide perception offers particularly interesting insights into how extra-oral TAS2R genes may mediate frogs’ interactions with endogenous and exogenous chemicals. We identified bufadienolide-sensitive receptors in cane toad and bullfrog (bullfrog TAS2R51/61, cane TAS2R56/58) (Fig 6C and 6D). Cane TAS2R56 is expressed in the oral cavity, suggesting the contribution to avoidance of ingesting prey containing bufadienolide or other toxic cardiac glycosides, such as toads (and their tadpoles) or specific insects. Bufadienolide-sensitive receptors are also expressed in liver and/or intestines, which might be related in postprandial responses to toxin consumption, such as activation of detoxification pathways. Furthermore, cane toads, which produce bufadienolides in their dorsal parotoid glands, have a skin TAS2R (cane TAS2R58) sensitive to both tested bufadienolides (marinobufagenin and cinobufagin; Fig 6C and 6D). This raises the possibility for feedback regulation of chemical defense pathways. None of the receptors we tested could detect the golden poison-dart frog toxin, batrachotoxin, within the tested concentration (5 μM), although we only evaluated a small fraction of dart frog receptors (Fig 6C). Hao and colleagues reported chloramphenicol, helicin, and D-salicin as agonists of bullfrog TAS2R17 called as T2R16 in Hao et al. 2023 [3], but we didn’t find any significant responses to these chemicals in our assay system. Since the sequences of receptors in both studies are identical and the reported responses are only moderate, this result may be due to differences in the assay systems.

These cellular assays indicate the functional potentials of selected TAS2Rs expressed either in oral or non-oral tissues to detect not only the bitter compounds known as other vertebrate species but also the substances relevant to amphibian ecology.

## Discussion

Our unified gene mining workflow across over 600 vertebrates indicates that in frogs and salamanders (batrachians) the TAS2Rs gene family experienced a rapid expansion, leading to a wider repertoire of these genes than any other lineage of vertebrates examined to date. Comparative phylogenetic analyses suggest that this expansion may have been allowed by a higher gene duplication, perhaps influenced by higher recombination rates in TAS2R clusters, as well as a much higher retention rate (i.e., lower loss rate) than in other lineages. Furthermore, our analyses suggest that this expansion may have been related to batrachians experiencing an adaptive shift towards a higher number of genes.

Our results generally agreed with the recently reported by Policarpo et al. 2024 (425 commonly analyzed assemblies; S1 Fig) [5]. The estimated number of intact TAS2Rs were identical in approximately 60% of species between the two studies. Conversely, about 20% were slightly larger in one study and the remaining 20% were slightly larger in the other study. This may be caused by the differences in the gene identification pipeline including the initial BLAST search parameters (e.g., threshold of evalue cutoff and query sequences) and the transmembrane prediction software. For instance, we did not find any TAS2Rs in cartilaginous fish unlike in recent literature [5,79]. Upon careful review of our intermediate results, we did find the same genes among our BLAST hits, but with scores that disqualified them at the early stages of filtering. Previous studies used a significantly more permissive evalue than we did (1e-5 as opposed to 1e-10) and different query sequence dataset, which may explain their greater sensitivity. This comparison suggests that the parameter settings of the gene mining method have no small impact on the results of gene repertoire search. Future genome mining studies could benefit from including validated cartilaginous fish TAS2R sequences in their training set. The additional assessment of our pipeline is described in the Material and Method section.

We explore possible genomic mechanisms that might have contributed to the massive expansion of TAS2Rs in batrachians. Zhong et al. demonstrated that TAS2Rs in three species of frogs are concentrated near the telomeres [4], where recombination is swiftest for many chromosomes [63]. We show that this is indeed the case for all vertebrates, and that this effect is considerably more pronounced in amphibians. We also show that batrachian TAS2Rs are typically located in clusters, and that these clusters are both larger and more numerous than those in other species. We also find that these cluster loci can be very old (up to 350 million years), even though the genes within them appear comparatively young, either due to rapid gene turnover or gene conversion. The high levels of gene turnover in some TAS2R lineages suggest that there may be some degree of interchangeability between genes, perhaps because they fulfill similar functions, and are therefore able to compensate for the loss of other genes. Overall, our detailed gene cluster analysis across vertebrates suggests that the frequent formation and expansion of TAS2R clusters due to their local genomic environments may be a major driver of the large and lineage-specific TAS2Rs repertoires in amphibians. Conversely, around 7–27% of genes experience lower rates of duplication (i.e., CNCOs). These genes were found to be more common in lineages with stable TAS2R counts, suggesting that there may be purifying selection against the duplication of these genes in some lineages. It is possible that a shift in the selective landscape of these genes in batrachians may have released these pressures, allowing for the copy number increase of previously constrained TAS2R loci, contributing to the expansion of the batrachian TAS2R repertoire.

To explore TAS2R functions in amphibians, we quantified their expression levels across a variety of ecologically and phylogenetically diverse amphibian species. Inspired by the extra-oral receptor studies that have been performed in humans, mice, fish, and chickens [31,33–36], we performed transcriptome analysis of seven different tissues. To our knowledge, this is the first search for extra-oral TAS2R expression in amphibians. The number of TAS2Rs expressed in the tongue was the highest among the seven tissues, despite differences in the proportions among species, suggesting that the amphibian tongue (or oral epithelium in *Xenopus*) is the primary tissue in which TAS2Rs function. The clawed frog had the vast majority of TAS2Rs expressed in the tongue at significant levels, similar to humans and mice [11,74]. By contrast, other amphibian species had proportionately fewer receptors expressed in the tongue. Indeed, the number of expressed receptors that are not found in the tongue is directly proportional to the number of TAS2Rs in the genome. This suggests that increases in TAS2R number may have allowed for the acquisition of tissue-specific functions in specific genes (i.e., gene subfunctionalization).

The receptors we chose for follow-up functional characterization illustrate the potential importance of extra-oral TAS2Rs in amphibians. As in previous literature, in each species some receptors responded to multiple stimuli, while others appeared specialized [10–12,25,27]. Interestingly, we find that three of the five species have liver-expressed receptors for the hepatotoxin aflatoxin B1. This suggests a potential contribution of TAS2Rs for the detection and/or detoxification of hepatotoxin (or other similar toxins) in the liver of amphibians. We also find that two large bodied frogs have receptors recognizing some frog toxins in the oral cavity and/or extra-oral tissues, which could be involved in the detection of dietary toxins. For instance, bullfrogs have intestinal/liver receptors for marinobufagenin, which is produced by cane toads and their relatives, and is similar to other cardiotonic steroids (i.e., cardenolides) present in a wide variety of toxic plants and animals. The oral receptors possibly contribute to the decision whether frogs ingest bufadienolide (or cardenolide)-laden prey such as toads (including their eggs and larva [80–82]), or cardiotoxic insects. We also find that cane toads have receptors for their own toxin, marinobufagenin, not only in the tongue but also in their skin. We speculate that the skin receptor may allow them to assess the levels of toxin secretion in the skin. While our study has focused on toxic xenobiotics, it might be fruitful to search for endogenously produced TAS2R ligands such as steroid hormones and bile acids [27,83].

Based on our expression and assay data, we propose that extra-oral TAS2R activity may be important for amphibian responses to toxins in their diet, their own chemical arsenal, and perhaps their environment. Future studies are needed to determine the behavioral and metabolic consequences of TAS2R activation. For instance, one might predict that activation of liver-based aflatoxin receptors could induce behaviors like vomiting, diarrhea, or avoidance, or increased expression of detoxification enzymes. Likewise, a cane toad that has received confirmation of the presence of toxins in its skin or glands via its TAS2Rs might take more risks, while inhibition of these receptors might induce toxin production or hiding behaviors. Furthermore, we interpret cross-frog toxin recognition as evidence of a chemical dialogue between species, where toxin recognition—and perhaps response—is achieved at least partially within the gut.

Ever since amphibians’ large TAS2R repertoire was first noted in the 2000s [84,85], scientists have searched for an explanation [3,25]. We propose that several TAS2Rs may fulfill tissue-specific functions in extra-oral tissues, with a different list of receptors in the tongue guiding feeding behavior; in the intestines and liver guiding metabolism; and in the brain controlling levels of stimulation. This specialization may have been made possible by a high recombination genomic environment, with genes preferentially near the telomeres and located in large clusters. The mutational input (i.e., gene duplication, loss, and conversion) promoted by recombination may have provided the raw material for novel expression patterns to arise, and perhaps allowed for rapid expansion and diversification of TAS2Rs within the batrachian lineage. We find that this is analogous to the way in which plant specialized metabolism genes, which are also organized in clusters, have multiplied, and diversified within lineages [86]. Considering the prominent and important extra-oral functions of TAS2Rs, we suggest that amphibian TAS2Rs be considered as “specialized chemosensory receptors,” to emphasize their broad range of proposed functions beyond conscious oral taste.

## Materials and methods

### Ethics statement

All animal care and use protocols were approved by the University of Michigan’s Institutional Animal Care and Use Committee (protocol # PRO00010325) and Massachusetts Institute of Technology’s Committee on Animal Care (protocol # 2205000363 and 2203000293).

### Genome data

We downloaded all vertebrate genome assemblies at the chromosome-level or greater from the NCBI Genome database on May 24, 2023. This amounted to 1,059 genomes, all of which were initially processed by our bitter receptor gene identification pipeline (described below). A small number of genomes failed in the pipeline, usually because of problems with the formatting of the genome sequence file. In the majority of our analyses, we only used one genome per species. In the cases where multiple genomes were present, we preferentially selected reference genomes. The only two exceptions were the human and zebrafish genomes, where the reference contained alternate assembly data and thus might include duplicate copies of genes. After a careful review of the alternatives, we selected the human genome from the Japanese Reference Genome Assembly project (GCA_014905855.1) and a zebrafish genome from a long-read sequencing experiment (GCA_020184715.1). We also removed genomes from hybrid organisms and subspecies retained a single subspecies for species with assemblies available for multiple subspecies. Nineteen genomes including special characters in common species names, such as apostrophe and ampersand, were removed because they consistently returned errors in the gene identification pipeline. Our final list contained 661 unique genomes, including 271 ray-finned fish (41%), 1 lungfish (0.15%), 26 amphibians (3.9%), 111 birds (17%), 9 cartilaginous fish (1.4%), 3 lampreys (0.44%), 35 reptiles (5.3%), and 205 mammals (31%). To assess the completeness of each genome assembly, we obtained a BUSCO score for each assembly. About 200 BUSCOs were run locally, and the remaining scores were pulled from Policarpo et al. 2024 [5] and NCBI. Five assemblies showed less than 70% completeness of BUSCO genes. Twenty-four assemblies showed 70–80%, while the other 632 assemblies showed over 80%. Some lamprey genomes had less than 50% BUSCO genes but this is possibly due to the uniqueness of jawless fish genomes [5]. Thus, except for lampreys, most genomes had nearly 70% or more BUSCO genes. We included all 661 assemblies for this study. Details and results for each genome are available in S2 Data.

In the transcriptomics phase, we used additional lower-quality genomes for particularly relevant amphibians. For the cane toad, we used assembly GCA_900303285.1 (accessed 5/31/22) and for the American bullfrog, we used GCA_002284835.2 (accessed 8/30/21), both of which were reference assemblies at the time of download. For the golden poison-dart frog we used assembly [Genbank: JBBPXS000000000], which was graciously provided prior to publication by Dr. Denis Machado [87].

Statistics about each genome were recorded using the Biopython Entrez command esearch, querying the NCBI assembly database. Genome size was accessed from the Animal Genome Size Database (AGSD) whenever possible [51]. Additional genome sizes for species not available in the AGSD were obtained from Liedkte et al [52].

### TAS2R gene identification

We analyzed each genome using a custom gene identification pipeline. We started by creating a database of known TAS2R sequences from *Ambystoma mexicanum*, *Danio rerio, Homo sapiens, Microcaecilia unicolor*, *Rhinella marina,* and *Xenopus tropicalis* from Behrens et al. [27]. These species were queried against the target genome in a tBLASTn search [88]. Seven genomes led to segmentation faults by tBLASTn due to their large size, so they were separated into smaller fragments (maximum size 500 MB) and analyzed separately. For each BLAST hit, the surrounding region was pulled using the samtools faidx command. In most situations, we took 1500 bp upstream and downstream from the hit coordinates. In the case where the query sequence starts at 1 (meaning that the very beginning of the sequence is matched), we did not expand downstream, to avoid artificially elongating the sequence with in-frame AUGs before the true start codon. We then identified the largest open reading frame in this region, and discarded sequences that had original e-value scores greater than 1e-10, that had lengths less than 200 amino acids or greater than 500 amino acids, or that were exact duplicates of prior hits. Next, we performed a reciprocal blast search against the genome of a chosen well-annotated genome within the same taxonomic group (*Xenopus tropicalis* for frogs, *Gallus gallus* for birds, *Homo sapiens* for mammals, *Anolis carolinensis* for lizards, *Danio rerio* for ray-finned fishes, *Latimeria chalumnae* for non-ray-finned fishes). The coordinates of the closest hits were matched back to the coordinates of known TAS2Rs in that reference genome, and candidate genes that didn’t hit a validated TAS2R were discarded. Finally, we used the program TMbed to predict the membrane topology of the candidate gene, and discarded all results that did not have exactly 7 transmembrane regions. The output of the pipeline is a fasta file containing all validated TAS2R sequences and a GTF file describing the gene location.

Our results are largely in alignment with those from several recent publications, most notably Policarpo et al. [5] (S1 Fig). We evaluated the rare differences to better understand and contextualize our dataset. For each difference, we considered three explanations: a problem with our gene identification pipeline, differences in genome quality, and real biological variation between individuals of that species. As a potential example of a shortcoming of our pipeline, we note that we did not find any TAS2Rs among cartilaginous fish, unlike in Behrens et al. [79]. This may be caused by the differences in the threshold of evalue cutoff and query sequence dataset in initial BLAST searches as mentioned in the discussion section. As an example of genome quality impacting TAS2R count, we demonstrated that the newest version of the axolotl genome has 75% more intact TAS2Rs than the previous version. A prior publication by Behrens et al. [27] reported that axolotl has 45 intact genes and 45 pseudogenes from earlier assembly (GCA_002915635.2), which is substantially different from our result of 70 intact genes from the latest assembly (GCA_002915635.3). When we redid our analysis using the older assembly, we only found 40 intact genes, suggesting that our result is attributable to recent improvements in the axolotl assembly. The differences in gene counts for the earlier assembly between our pipeline and Behrens et al. [27] may be mainly caused by differences in the criteria of ORF (open reading frame) and transmembrane prediction (S5 Table). Finally, we showed that high-quality human genomes have between 22 and 26 TAS2Rs, but there was no significant correlation between TAS2R count and coverage, N50, or sequence length (p = 0.64,5, 0.053, 0.092, respectively). Twenty-six TAS2Rs are known to be present in modern humans [7], but we do not know of any prior examples of one individual containing all 26 as intact genes. Furthermore, we checked Ensembl’s Structural Variant viewer for each of the 25 canonical human TAS2Rs within the most current reference genome (GRCh38.p14), and found that every gene has both deletions and duplications, with the sole exception of hTAS2R1, which has only duplications [89,90]. Accordingly, we propose that this range may represent real biological variation, consistent with Hayakawa et al.’s report of varying TAS2R counts among humans [7] and chimpanzees [9].

### Comparative phylogenetic analyses

To investigate the macroevolutionary dynamics of TAS2R gene content, and to assess its correlation with associated metrics in a phylogenetically-aware way, we generated a time-calibrated phylogeny of our study species using the TimeTree database [91]. Species not available on TimeTree we replaced with a close relative whenever possible, as detailed in S6 Table. The final tree contained 645 of the 661 analyzed species. For compatibility with downstream analyses, branches of length 0 were assigned a length of 1e-5 MY if they were internal or 0.01 MY if they were tips.

We reconstructed ancestral TAS2R numbers using DupliPHY [46] across the entire phylogeny. To estimate gene duplication and loss rates for individual clades we fit birth-death models with separate birth and death rate parameters in CAFE v. 4.2.1 [54] (*lambdamu* function). Species trees for each clade were pruned from our vertebrate-wide phylogeny using the R package *ape* [92]. To ensure convergence, we performed 50 independent CAFE runs on each dataset, and selected the run with the highest final likelihood. Optimizations that resulted in “infinite” likelihood scores were considered failed and re-run. Attempts to fit this model to mammals or all vertebrates were unsuccessful, likely due to the depth of these clade’s phylogenies and the breadth of TAS2R variation across them.

We identified evolutionary regime shifts using R package *l1ou* [60], which implements a lasso regression approach to fit and rank large numbers of single and multi-optimum OU models [58,59]. Models with 0–50 shifts were considered, and rated based on the phylogenetically-corrected Bayesian information criterion [60]. To compare the fit of different models we calculated pBIC weights as


$$w_{i}=\frac{exp\left(-0.5\Delta_{pBIC_{i}}\right)}{\sum_{k=1}^{K}\;exp\left(-0.5\Delta_{pBIC_{k}}\right)}$$

where *w*_*i*_ is the pBIC weight for model *i*, Δ_*pBICi*_ is the difference in pBIC between model *i* and the best model (i.e., the one with the smallest pBIC), and *K* is the total number of models. To compare the fit of the best two OU models to their BM homologs we used the brownie.lite() and fitContinuous() functions in *phytools* [93] and *geiger* [94], respectively. In this case models were compared based on the AIC because the pBIC has not been implemented for Brownian Motion models. For all model fitting exercises gene family size was log-transformed as log(x+1).

Correlations between the number of TAS2R genes and other traits were assessed using phylogenetic generalized least squares (PGLS), as implemented in the phylolm R package [95], based on an OU covariance structure. All traits were log-transformed prior to analyses. If zeros were present, the transformation log(*x*+1) was applied. Given the large differences in the number of species with genome sizes available (n = 233), and those with data on TAS2R gene clustering (n = 543), genome size and clustering metrics were analyzed separately. However, a model with all predictors considered simultaneously produced qualitatively similar results (S7 Table).

### Phylogenetic tree inference

In order to make a phylogenetic tree with all 9,291 TAS2R sequences identified by the pipeline, we first used mafft (v7.520; Katoh & Standley, 2013) to create an alignment [96]. We first created a skeleton alignment using a high-accuracy method (genafpair with maxiterate 1000) and the TAS2R reference sequences used as queries (based on Behrens et al., 2021 and Li and Zhang, 2014; [27,72]), but with all pseudogenes removed. We also added six vomeronasal type 1 receptors from zebrafish as outgroups. Our pipeline-derived sequences were added to this skeleton using the mafft—add command (maxiterate 1000). Next, we inferred a maximum-likelihood gene tree using iqtree [97,98]. For computational efficiency we performed sequence evolution model selection using the skeleton alignment described above, and used the best-fitting model (JTT+F+R8) to infer a tree with all of our aligned sequences. Node support was evaluated using approximate Bayes (aBayes) scores [99].

To better visualize the relationships between genes in our five core amphibian species, we also made several versions of a tree which contains all sequences from the five amphibians of our transcriptomics study, in addition to several reference TAS2Rs and outgroups. In addition to the amphibian receptors identified by our pipeline, we included TAS2R sequences from *Homo sapiens, Mus musculus, Danio rerio, Microcaecilia unicolor, Anolis carolinensis, and Gallus gallus* from our gene identification pipeline and *D. rerio* ORA outgroup sequences from the NCBI gene portal [100]. After alignment in mafft and tree generation in iqtree (as described previously), tree files were manipulated in R with the packages ape [92], phytools [101], and TreeTools [102]. In S15, S17 and S18 Figs, phylograms are displayed next to heatmaps which summarize expression across tissues. In Fig 5, the tree underlying S15 Fig is subsetted, and icons are included to highlight patterns of expression, using thresholds as indicated in the legend. We also have a nucleic acid alignment with the same annotation in S17 Fig, as well as an amino acid alignment created using expression data with the -M flag in S18 Fig, which allows for multi-mapping.

### Gene position and cluster identification

In order to determine the location of each gene within its chromosome, we first applied a script that analyzes each contig in the genome to determine whether it is a full chromosome (contains “Chromosome”, “chromosome”, “chr”, “linkage group” or “LG” in the header row) and how long it is. For each gene located on a chromosome (and not on an unplaced contig), we then calculated the distance from the nearest end with (min ((chromosome length − gene start position)/chromosome length, 1 − (chromosome length − gene start position)/chromosome length)). The quality parameters of the chromosome-scale genome assembly (i.e., contig N50 and BUSCO gene completeness) are not likely to globally have large effects on the relative gene positions, although several gene positions were slightly correlated with contig N50 and/or BUSCO gene completeness in human or zebrafish genomes (S19 and S20 Figs). In contrast, the relative positions of specific genes can be affected by local structural variation (sometimes by incorrect assembling) and local assembly contiguity in each chromosome (S21 Fig). We combined data from all unique validated genomes and plotted the results as a histogram in R. We also did analyses focusing on several taxonomic categories, including amphibians, non-amphibians, and non-snake lizards.

We considered several different methods for defining clusters. In the simplest model, two consecutive TAS2Rs are considered to be clustered if their start codons are within a certain distance. Singleton genes are not considered to be clusters. We considered variants of this method with maximum gap sizes of 100 kb, 200 kb, 500 kb, 1 mb, 2 mb, and 5 mb. In a slightly more complex analysis, called the “median method,” we started with a fixed gap distance and then calculated the median gap distance for a given cluster. If any individual gaps were greater than a certain number of median gap sizes, we divided the cluster at this point into two (or more) subclusters. We explored various initial gap sizes and median methods, and selected 1 mb and 20 medians. Ultimately, we compared these 7 methods (6 different fixed gaps and the median method with 1 mb and 20 median) and found that all of the key conclusions were unchanged. These results are available in S4 Fig. In the body of our paper, we use a fixed gap of 1 mb, which had intermediate clustering values that closely matched human intuition of appropriate clustering. Once clusters were defined, we calculated the following parameters for each genome: number of clusters, average genes per cluster, fraction of TAS2Rs located in a cluster, fraction of genes located in biggest cluster, size of the biggest cluster in kb, total length of all clusters in kb, and average kb/receptor. Next, we used mafft to create an alignment of all TAS2R sequences from the genome, created a distance matrix using distmat, and we determined whether each gene was in the same cluster as its most similar other gene. The percent for which this was true was reported as the parameter “nearest in cluster.”

Clusters were diagrammed using a custom script written by Matthew Hill (see our GitHub repository, https://github.com/kwhiggins27/amphibian-TAS2R). From user input, the script identifies the start and stop genes for the cluster illustration. It then scans the genome.gff for these gene features and all gene features between them, identifying their start/stop coordinates and strandedness (i.e., direction). It then produces a python script that, when run, constructs the diagram using DnaFeaturesViewer [103]. In the output, the genome is represented as a horizontal line, and genes are reproduced from the direction and start/stop information contained in the gff. The drawing is also padded out with blank space in both directions by 1% of the distance between the two genes.

### Identification of repeat elements

We wanted to determine if repeat elements are enriched in the proximity of TAS2Rs using a similar method to Syed and Korsching [66,104]. We started with a list of all amphibian genomes in our species list, and added a random assortment of 24 vertebrates belonging to other classes. For each species, we used RepeatModeler to generate a species-specific repeat library using default settings. Next, we used Repeat Masker to identify repeat elements within the 100 KB upstream and downstream of a TAS2R cluster, or in 10 randomly selected 100 KB intervals. Our analysis includes LINEs, SINEs, LTRs, DNA-elements, and all masked bases. After excluding species that do not have clusters, we performed paired t-tests for each of each repeat element type. Out of the five tests, only LTRs rejected the null hypothesis of no difference with p = 0.046 (mean of 7.3% for cluster vs 5.6% for random). We also performed separate tests for amphibian and non-amphibian populations. Among amphibians, nothing was statistically significant. Among non-amphibians, total repeat elements and LINES were significantly elevated and SINES were significantly depressed.

### Identifying syntenic blocks surrounding TAS2Rs

In order to identify orthologous TAS2R clusters between species, we relied on neighboring marker genes. We ran BUSCO to identify conserved single copy orthologs in about 39% of our species (261 out of 661) and identified which genes fall within 1 MB of a TAS2R cluster or singleton gene (“TAS2R loci”). Next, we filtered for loci that contained at least two BUSCO marker genes, and then used a hypergeometric test to determine the probability that any two TAS2R loci were related by chance [105]. The resulting p-values were adjusted for multiple hypothesis testing with a Bonferroni hypothesis. A networking analysis was performed with networkx to convert the list of linked pairs into a list of conserved clusters [106]. For each cluster, we determined the minimum age using our species tree from Fig 1A and the paleotree functions getMRCA and dateNodes.

Of note, this methodology for calling clusters is extremely conservative, and excludes potentially homologous clusters that are not near two or more BUSCO genes, or for which the BUSCO genes have been rearranged over time. Thus, we cannot use this method to conclusively say that a TAS2R cluster is not present in a certain lineage, and we can only state the minimum age of a conserved cluster (and not the true age).

### Scoring gene evolution between trios of vertebrates

In order to visualize the frequency of major evolutionary events, we performed a methodical manual analysis of phylogenetic trees containing specifically-chosen trios of three species. Each trio was chosen either to highlight specific species of interest (ex: humans) or to explore an intriguing TAS2R difference, like the fact that the saltmarsh sparrow (*Ammodramus caudacutus*) has 14 TAS2Rs while the closely related dark-eyed junco (*Junco hyemalis*) has 3. A third outgroup species was chosen to be slightly more distantly related, and roughly representative in TAS2R count of similarly-related species. Each tree is a subset of the massive amino acid alignment-derived maximum likelihood tree in Fig 2, with all tips dropped except those corresponding to the desired three species. To visualize node confidence, we put a dot on each node corresponding to the aBayes value (red: aBayes > 0.95, orange: 0.90 < aBayes < 0.95, yellow: aBayes < 0.90). Based on this tree, we examined each TAS2R in our two test lineages. If one gene from each species branches together, we considered this a “one-to-one” pairing (traditional orthologs). If one gene from Species A is sister to a group of 2 or more genes from Species B (or vice-versa), we scored all of these genes as being in a “one-to-many” relationship. If multiple genes from Species A branch next to multiple genes from Species B, we considered this to be a “many-to-many” relationship. If a node contained one gene from Species A and no genes from Species B, this was a “one-to-zero” relationship. Finally, “many-to-zero” was defined as multiple genes in one species with no corresponding genes in the other.

### Illustrating cluster evolution

We used two different methodologies to illustrate cluster comparisons between species. In Fig 3B, we color a two-species tree with different shades of blue or orange for the saltmarsh sparrow and the dark-eyed junco, respectively, to show different cluster/singleton loci. We also include a chromosomal diagram which shows where each locus falls along its chromosome. Chromosomal locations are defined as the position of the start of the locus divided by the length of the chromosome. Locus names were assigned as shown in S1 Data. When two clusters were located too close together to resolve, the distance between them was slightly increased for clarity of illustration.

In S11 Fig, we use a slightly different format to illustrate the same underlying data for two pairs of frog species. Chromosomal diagrams were created in the same way, but they were placed vertically in species pairs in between mirrored trees. Since frogs have a larger number of clusters than we could reliably distinguish color shades, we needed an alternative way to illustrate locus identity. Instead, we drew a line between a gene’s location on the tree to its approximate location on the chromosomal diagram. In the case of extremely close clusters, the line terminus was placed slightly above or below to accentuate the difference.

### Identifying copy number constrained orthologs

In order to identify CNCOs, we had to divide TAS2Rs into gene families. First, we subsetted the large phylogenetic tree in Fig 1D into amphibians, mammals, birds, reptiles, and ray-finned fish. Moving through each tree, we split genes into gene families such that there was minimal repetition of each species (ignoring very recently duplicated genes) and no crossing of deep branch points. Whenever we encountered a very young gene family (less than 50 million years old) or a family containing only one species, we tried to expand it while still following our rules. If the situation couldn’t be resolved, we noted it as a gene family but did not include it in further analysis. For each qualifying gene family, we identified the list of expected species by taking the two most distant species represented and listing all species that occur between them on the species tree in Fig 1A. For each expected species, we determined whether there were 0, 1, or 2 or more copies of the gene. If less than 5% of species had two or more genes and more than 50% had exactly one, we considered the gene family to be a CNCO. Once gene families were assigned, we created a final table containing each gene, the assignment of its gene family, and whether or not it is in a gene cluster (as previously identified).

We were curious whether CNCOs were enriched in certain clusters. In order to determine whether this was true, we took the distribution of cluster sizes for each lineage (amphibian, mammal, etc) and the proportion of genes that are CNCO in each lineage, and used a binomial distribution to calculate the expected number of clusters containing exactly one vs more than one CNCOs. From these theoretical results, we calculated the expected probability that a cluster has a second CNCO TAS2R, given that it already has one. We compared these proportions to the true proportions using a two-proportion z-test. For amphibians and mammals, the true proportion was significantly greater than the theoretical proportion, demonstrating that CNCOs are enriched in certain clusters. For birds, reptiles, and fish, the number of CNCO-containing clusters was extremely low and the results were not significant.

### Tissue harvest, RNA isolation, and sequencing

Experimental animals were obtained from either laboratory colonies (*P. terribilis, A. mexicanum*) or commercial vendors (*X. tropicalis*, *Rh. marina*, *Ra. catesbaiana*). Specifically, axolotls were obtained from the Ambystoma Genetic Stock center (leucistic strain, adults), bullfrogs from 168 (Dearborn Heights, MI; adults), cane toads from Carolina Biological (adults), golden dart frogs from the colony one of the authors’ (RM) captive lab colony (juvenile), and clawed frogs from Xenopus 1 (Nigerian strain, juvenile). Frogs were euthanized through an overdose of topical benzocaine applied ventrally on the femoral patches and lower venter, while axolotl were euthanized by immersion in 0.1–0.2% tricaine for one hour, followed by decapitation. All animals were immediately dissected for tissue harvest. Tongue, brain, liver, stomach, intestine, and dorsal and ventral skin samples were taken from each animal and immediately placed in Qiagen buffer RLT with added beta-mercaptoethanol over ice for RNA extraction. For the tongueless *X. tropicalis*, we dissected the epithelium from the floor of the oral cavity instead of the tongue. The oral epithelium is known to contain taste buds in Xenopus frogs [107,108], and vestigial tongue musculature is present in the floor of the mouth [109], so we consider this comparison adequate. For simplicity, we refer to this tissue as the “tongue” across species. A set of backup samples was stored in RNAlater at 4 °C for two days and transferred to −80 °C for permanent storage.

Immediately following tissue harvest, we extracted total RNA using the Qiagen RNeasy Fibrous Tissue Mini Kit following the manufacturer’s protocol. For the tissue homogenization step, we ground the sample for 2 minutes at 4 °C using a hand-held mechanical homogenizer (Omni TH for axolotl, Tissue Tearor, for frogs). After extraction, samples were kept on dry ice. Subsequently, quality control analysis and quantification were performed using a Fragment Analyzer. If the RNA integrity number (RIN) for a sample was below 7, the backup sample was defrosted and processed by the same protocol, and the sample with the superior RIN score was used for library preparation. Backup samples were substituted for primary samples for 9 bullfrog tissues (all three stomach and liver replicates, intestines replicate 2 and 3, ventral skin replicate 2). Of note, we were not able to obtain desirable RIN scores for 4 samples despite 2–4 attempts (bullfrog stomach replicates 1 and 2, bullfrog liver replicate 3, and axolotl liver replicate 2). In these cases, the highest quality sample was used. We performed principal component analysis (PCA) to determine whether there was any difference between fresh or frozen samples, or between low RIN scores and desirable RIN scores (S22 Fig). Bullfrog liver replicate 3 does separate substantially from the other clusters, but in a way that is comparable to the way in which tongue samples separate in cane, dart, and clawed frog samples (all of which had excellent RIN scores). We conclude that all of our sequencing reads are of sufficient quality to include in analysis. In later downstream analysis, we did not note any problems that we believe could be attributed to poor sample quality.

Libraries were prepared for RNA-Seq using IDT’s xGen RNA Library Prep Kit, according to manufacturer’s directions. First, total RNA (100 ng–1 ug) is enriched for polyadenylated sequences using NEBNext poly(A) mRNA Magnetic Isolation Module (NEB). The enriched mRNA fraction is then fragmented, and first-strand cDNA is generated using random primers with a “stubby adapter” overhang. An Exonuclease step removes excess RT primers and prevents them from becoming template downstream. Via an “Adaptase” step, simultaneous tailing and ligation add a second stubby adapter. A final PCR amplification step adds indexes and sequences needed for flow cell binding. Strand specificity is achieved by bypassing the traditional second strand cDNA synthesis step prior to adapter ligation. After sample preparation, samples were sequenced on an Illumina HISEQ with 200 bp–600 bp fragments. The raw sequencing reads have been submitted to NCBI SRA (accession PRJNA1033547).

### Transcriptome alignment and quantification

We quality-trimmed and removed adapter sequences from the raw reads with Trimmomatic (v. 0.39; Bolger et al., 2014) [110] using an extensive list of Illumina adapters distributed with BBMap v. 38 [111]. Next, we created a custom STAR index using the genomes described above and the GTF files produced by our gene-identification pipeline for the annotation (v 2.7.1a) [112]. Given that several of our genomes contained over a million contigs, we reduced the parameter --genomeChrBinNbits to 12 and set --genomeSAsparseD to 2, as per the creator’s recommendation. We also raised --limitGenomeGenerateRAM to 200GB to allow for increased memory. Once an index had been created for each genome, we aligned each filtered raw reads file to the genome and index using STAR 2.7.1a with the parameters --outSAMtype BAM SortedByCoordinate, --quantMode TranscriptomeSAM, and --genomeSAsparseD 2. Given that we were using a custom GTF that doesn’t have genes on every chromosome, many of the standard quantification programs were inappropriate or not applicable. We chose to use featureCounts with default parameters, which ignores all multi-mapping reads (v 2.0.1) [113]. We did repeat the initial steps of our downstream analysis using the -M flag which splits multi-mapped reads evenly between all matched targets, but this did not affect our conclusions (see S15 Fig vs S18 Fig). Next, we calculated fragments per kilobase of transcript per million mapped reads (FPKM) as shown in the equation below.


$${\text{FPKM = (RM}}_{\text{g}}{\text{x 10}}^{\text{9}}{\text{)/(RM}}_{\text{t}}\text{x L)}$$

Where RM_g_ is the number of reads mapped to the gene, RM_t_ is the number of reads mapped to the genome, and L is the length of the gene in base pairs [114].

### Analysis of transcriptomics and selection of genes for follow-up

Results were compared and analyzed in python using the packages pandas, numpy, seaborn, matplotlib, os, and itertools. We calculated the number of genes expressed in each tissue with FPKM greater than 0.01, and the number of genes with FPKM greater than 0.01 in at least one tissue. We also calculated the number of genes present in exactly one tissue. Finally, we constructed colocalization matrices using the scipy function spearmanr, which calculates Spearman coefficients.

### In vitro functional assay

The responses of amphibian bitter taste receptors were measured using the luminescence based functional assay system, as previously described [28,115]. Eighteen amphibian receptors tagged with the first 45 amino acid sequences of rat somatostatin receptor 3 in N-terminus were synthesized by Twist Bioscience and subcloned into mammalian expression vector pEAK10 (Edge Biosystems) using a Gibson assembly. HEK293T cells were a generous gift of Dr. Hiroaki Matsunami (Duke University, USA). Cells were transiently transfected with the expression vector of a bitter taste receptor, human Gα16gust44, and mt-apoclytin-II using Lipofectamine 2000 (Thermo Fisher Scientific). Transfected cells were seeded into 96-well CellBIND surface plates (Corning) and incubated overnight at 37 °C with 5% CO_2_. Then, the culture media were removed and cells were loaded with the assay buffer and the luminescent substrate, coelenterazine (Promega) (10 mM coelenterazine, 130 mM NaCl, 10 mM glucose, 5 mM KCl, 2 mM CaCl_2_, 1.2 mM MgCl_2_, 10 mM HEPES, 0.1% BSA, pH 7.4), and incubated for 4 hours at 27 °C in the dark. Luminescence intensity was monitored with automatic application of substances using Flexstation III microplate reader (Molecular Devices, San Jose, CA). The response from each well was calculated based on the area under the curve (AUC) and expressed as relative light units (RLU) using SoftMax Pro 7.1 (Molecular Devices). We tested 22 substances with various chemical structures known as agonists of vertebrate bitter taste receptors and 6 substances potentially relevant to amphibian ecology (S4 Table). These substances were dissolved in the assay buffer or in dimethylsulfoxide (DMSO) followed by dilution in the assay buffer not exceeding a final DMSO-concentration of 0.5% (v/v). Data were obtained from at least 6 replicates, with no replicates excluded. The agonists were defined as substances to which receptors showed significantly higher responses than that to the assay buffer in each receptor (*p* < 0.05, two-sided Welch’s t-test with Benjamini-Hochberg correction) because the basal responses (responses to the assay buffer) are generally different among receptors and between receptors and no-receptor control. For substances to which at least one receptor responded, we confirmed that no-receptor control didn’t show a higher response than to the assay buffer (S23 Fig). For a subset of substances which were determined as agonists, dose-response relationships were examined. Significance of responses was tested between the responses in the lowest concentration and those in the other higher concentrations (*p* < 0.05, Dunnett’s test). To make a heatmap, the receptor activity was converted into the relative activity using the formula (response_chemical − response_buffer)/(response_max − response_buffer), where the response_chemical, response_buffer, and response_max indicate response to each tested substances, response to assay buffer, and response to the substance eliciting the strongest receptor activation in each receptor, respectively.

### Programming techniques

Scripting was performed in Unix, Python, R, and perl, based on the availability of bioinformatics tools. On several occasions, first drafts of code were written with aid from ChatGPT [116] with heavy editing afterwards.

## Supporting information

S1 Fig The comparison of intact TAS2R gene counts between this study and recent literature Policarpo et al. 2024 [5]. The number of TAS2Rs with complete 7TM topology was obtained from the S1 Data of Policarpo et al. [5]. 425 assemblies were commonly analyzed in both studies. The number of TAS2R genes was almost same between the two studies.
(PDF)

S2 Fig Correlation between genome size (c-value) and number of TAS2Rs identified in genomes for different taxa. Data shown separately for amphibians alone in the right panel.
(PDF)

S3 Fig Four best-fitting regime shift configurations inferred by l1 ou, accounting for roughly 97% of pBIC weight. Branches are colored by evolutionary regime, and numbers on branches represent the magnitude of the optimum shift (fold-change) for each regime. pBIC weights are labeled above each plot.
(PDF)

S4 Fig Comparison of different ways of defining TAS2R clusters. On the right is the underlying species tree, as described in Fig 1A. Heatmap shows the number of clusters identified in each species. For columns 100 K, 200 K, 500 K, 1 M, and 2 M, and 5 M, the name reflects the maximum allowed distance between two neighboring genes within the same cluster. “Median” refers to a more complex analysis described in the Methods section.
(PDF)

S5 Fig Additional features of TAS2R gene loci in genomes. (A) Boxplot showing fraction of all TAS2Rs that are clustered, showing only species that contain TAS2Rs. (B) Boxplot showing average kb per TAS2R within TAS2R clusters, showing only species that contain clusters.
(PDF)

S6 Fig Distance of TAS2R gene loci to either end of chromosome separately analyzed by qualities of genome assembly. (A-B) Distance to the nearest chromosome end of TAS2Rs in amphibians (left) and nonamphibians (right), corresponding to Fig 2E, colored by (A) BUSCO completeness score, and (B) contig N50. (C-F) Distance to the nearest chromosome end of TAS2Rs in amphibians (left) and non-amphibians (right) from the genome assemblies with (C) over 80% BUSCO completeness (BUSCO80)(D) over 90% BUSCO completeness (BUSCO90)(E) over 1 Mb contig N50, and (F) over 80% BUSCO completeness and over 1 Mb contig N50. Along the x-axis, 0 represents either end and 0.5 is the numerical center of the chromosome. The distance to the nearest chromosome end of TAS2Rs is significantly smaller in amphibians than non-amphibians in all conditions (Welch’s two-sample t-test(C): t = −11.88, df = 2378.9, p = 5.98e-32; (D): t = −3.04, df = 1150.5, p = 0.0012; (E): t = −13.59, df = 3202.9, p = 2.98e-41; (F): t = −10.73, df = 2109.0, p = 1.73e-26).
(PDF)

S7 Fig Barplots showing the percent of various regions identified as being repeat elements using RepeatMasker and RepeatModeler. “Cluster” refers to the 100 KB immediately upstream and downstream from each TAS2R cluster in each lineage. “Random” refers to randomly selected 100 KB regions of the genome. Data shown for 15 amphibian species in comparison to 12 non-amphibian species. Comparisons marked with an asterisk (*) are significantly different with p<0.05 in a one-sided paired t-test.
(PDF)

S8 Fig Comparison between TAS2R repertoires of closely related species, based on the tree in Fig 2, as described for Fig 3A. (PDF)

S9 Fig Proportion of cluster and singleton TAS2Rs that are “copy-number-constrained orthologs” (CNCOs). As a brief recap of the text, these genes are present in exactly one copy in greater than 50% of species and are duplicated in fewer than 5% of species.
(PDF)

S10 Fig Conserved singleton TAS2R locus from two distantly related fish, the sablefish (*Anoplopoma fimbria*, left) and the mangrove rivulus (*Kryptolebias marmoratus*, right). Conserved BUSCO genes are shown in bright colors, matching between the two panels.
(PDF)

S11 Fig Diagram relating the location of TAS2Rs in a phylogenetic tree to their location along the chromosome. Genes from the common frog (*Rana temporaria*) are shown in amber on the left and genes from the wood frog (*Lithobates sylvaticus*) are shown in forest green on the right. Gene location along the chromosome is sometimes accentuated to distinguish very close clusters but is accurate within about 5% of the chromosomal length.
(PDF)

S12 Fig Number of TAS2Rs detected with FPKM > 0.01 per tissue per animal. (PDF)

S13 Fig Percent of expressed receptors that are unique to exactly one tissue, by tissue and by species. (PDF)

S14 Fig Heatmap of tissue expression across amphibian tissues with hierarchical clustering applied to the tissue samples. Dendrogram colored according to standard evolutionary biology colors for ectoderm (blue) and endoderm (yellow), with tissues with mixed lineages in black.
(PDF)

S15 Fig Relationships between TAS2R phylogeny (based on the amino acid alignment) and mRNA expression. A phylogenetic tree showing the relationship between amphibian sequences based on an amino acid alignment, next to a heatmap showing the expression of each receptor in seven different tissues. Alternate versions of this diagram are available in S17 and S18 Figs.
(PDF)

S16 Fig Agonist screenings of eighteen amphibian TAS2Rs. Eighteen amphibian TAS2Rs were assayed using luminescence-based functional assays with 28 substances (see S4 Table). The responses to 6 substances potentially relevant to amphibian ecology, 22 classic bitterants, and assay buffer were represented in pink, light blue, and white, respectively (n = 6–9 for chemicals, n = 21–25 for assay buffer). Asterisks indicate significantly higher responses compared to the response to the assay buffer (two-tailed Welch’s t-test with Benjamini-Hochberg correction, a = 0.05).
(PDF)

S17 Fig Relationships between TAS2R phylogeny (based on the nucleic acid alignment) and mRNA expression. Phylogenetic tree showing relationship between amphibian sequences based on a nucleic acid alignment, next to a heatmap showing the expression of each receptor in seven different tissues. After this diagram was found to be qualitatively similar to S15 Fig, S15 Fig was used in all subsequent analyses. Alternate versions of this diagram are available in S15 and S18 Figs.
(PDF)

S18 Fig Relationships between TAS2R phylogeny (based on the amino acid alignment) and mRNA expression including multi-mapped reads. Phylogenetic tree showing relationship between amphibian sequences based on an amino acid alignment, next to a heatmap showing the expression of each receptor in seven different tissues with multi-mapped reads. After this diagram was found to be qualitatively similar to S15 Fig, S15 Fig was used in all subsequent analyses. Alternate versions of this diagram are available in S15 and S17 Figs.
(PDF)

S19 Fig Correlations between the human TAS2R positions and quality scores of genome assemblies. The scatter plots show relationships between TAS2R positions in human genome assemblies and (A) BUSCO completeness and (B) contig N50. Inset numbers indicate R-squared and coefficients of linear regression models (y = ax+b), where x and y indicate BUSCO_score/contig_N50 and gene_position, respectively. P-values represented as * (p < 0.05), ** (p < 0.01), and *** (p < 0.001).
(PDF)

S20 Fig Correlations between the zebrafish TAS2R positions and quality scores of genome assemblies. The scatter plots show relationships between TAS2R positions in zebrafish genome assemblies and (A) BUSCO completeness and (B) contig N50. Inset numbers indicate R-squared and coefficients of linear regression models (y = ax + b), where x and y indicate BUSCO_score/contig_N50 and gene_position, respectively. P-values represented as * (p < 0.05), ** (p < 0.01), and *** (p <0.001).
(PDF)

S21 Fig Comparisons between TAS2R gene loci and qualities of genome assembly in humans and zebrafish. TAS2R loci were compared among chromosome-scale assembly of human (top left) and zebrafish (bottom left) genomes with various qualities (T2T assembly in red). The chromosome 12 in the assembly GCA_00002115.2 had a structural variation between a chromosomal end and TAS2R loci, which affected relative positions of TAS2R loci in the chromosome 12 (top right). In the chromosome 8 of GCA_008692375.1, there were many inversions possibly due to the incorrect assembling, which may have influenced positions of some genes (bottom right). Dot plots were generated by Unipro UGENE software.
(PDF)

S22 Fig Principal component analysis comparing TAS2R expression levels across tissues. For axolotl and bullfrog, note that certain samples were of poor quality (based on RIN scores) or were briefly frozen, as indicated with different shapes. For cane, dart, and clawed samples, all passed the RIN score threshold and only fresh samples were used.
(PDF)

S23 Fig Cellular responses of no-receptor control in the functional assay. No-receptor control was assayed using luminescence-based functional assays with the substances to which at least one receptor responded (20). The responses to substances and assay buffer were represented in gray and white, respectively (n = 6 for chemicals, n = 14 for assay buffer). There were no significantly higher responses compared to the response to the assay buffer (two-tailed Welch’s t-test with Benjamini-Hochberg correction, α = 0.05).
(PDF)

S1 Table Percent of genomic regions identified as repeat elements of various kinds in amphibians. (PDF)

S2 Table Percent of genomic regions identified as repeat elements of various kinds in nonamphibians. (PDF)

S3 Table Orthologous loci (TAS2R clusters and singletons) identified using conserved neighboring BUSCO genes. (PDF)

S4 Table Compounds used in functional assay. Alphabetical by compound, with classic bitterants shown first and project-specific natural products at end.
(PDF)

S5 Table The comparison of intact TAS2Rs in an axolotl genome assembly between gene mining methods. The comparison of intact TAS2Rs in an axolotl genome assembly GCA_002915635.2 between this study and Behrens et al. 2021 [27].
(PDF)

S6 Table The list of species whose names were replaced by close relatives in our analysis. These species were present in our TAS2R database but an exact match was not available on TimeTree. We substituted a closely related species as indicated in this table.
(PDF)

S7 Table Fit parameters for various phylogenetic generalized least squares models, assuming covariance between phylogeny and traits under an OU model. Model formula was: log(Number.of.Genes.x+1)~log(clusters+1)+log(genes_per_cluster+1)+log(genome_size)
(PDF)

S1 Data The list of TAS2R genes discovered in our pipeline. This spreadsheet contains information about the TAS2Rs discovered in this pipeline. The first tab (“pipeline”) contains information on the 9,291 genes discovered through systematic analysis of 661 chromosome-level assemblies of vertebrate genomes. The column entitled “big” tallies whether or not a genome was analyzed by the “big genome” method or the standard analysis. For these species, both internal and original coordinates are given. For all other genomes, these coordinates match. The tab “human” contains information on genes discovered in several additional human chromosome-level assemblies, chosen as described in the methods. The tab “5 amphibian” contains information on the five amphibians studied in additional detail in this project. For the species with chromosome-level assemblies (axolotl, western clawed frog), gene names match those in the pipeline tab. The tab “followup?” contains notes on why specific receptors were selected for functional follow-up, when applicable.
(XLSX)

S2 Data Summary data for 661 species analyzed in this study. This spreadsheet includes summary data for each of the 661 species with chromosome-level assemblies processed in the first phase of this project. Columns class, order, latin, common, taxid, contig_count, contig_I50, contig_n50, total_length, and coverage were pulled from the NCBI assembly database for each accession number. PlottingClade refers to the taxonomic group that was used for plotting in Figs 1 and 2. All other columns were derived as described in the Methods.
(CSV)

S3 Data Gene tree file underlying Fig 2. Newick tree file underlying Fig 2, with receptors named as shown in S1 Data (“pipeline” tab, “tree_name” column).
(TXT)

S4 Data Gene tree file underlying S13 Fig. Newick tree file underlying S13 Fig, with receptors named as shown in S1 Data (“5amphibian” tab, “short_name” column).
(TXT)
