# Supplementary figures and images for: Rapid expansion and specialization of the TAS2R bitter taste receptor family in amphibians
Source: PLoS Genet. 2025 Jan 31;21(1):e1011533. doi: 10.1371/journal.pgen.1011533 (PMC11798467; doi:10.1371/journal.pgen.1011533)

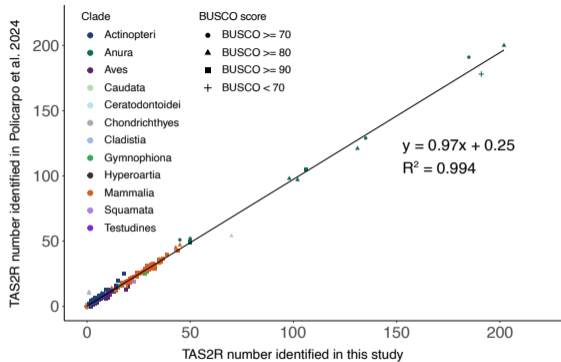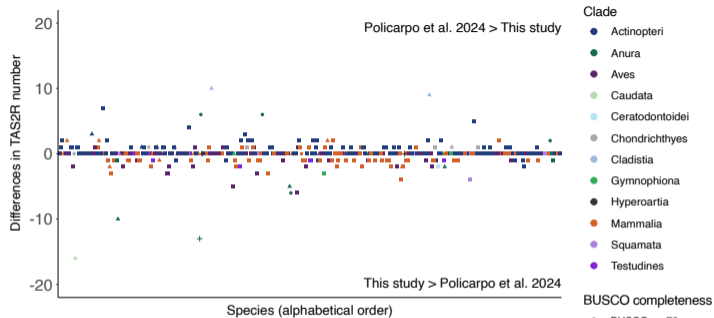

|           | Policarpo et al. > This study | Policarpo et al. = This study | Policarpo et al. < This study |
|-----------|-------------------------------|-------------------------------|-------------------------------|
| Assembly  | 89                            | 256                           | 80                            |
| Ratio (%) | 20.94                         | 60.24                         | 18.82                         |

Supplement: S1 Fig — 2024 [5]. The number of TAS2Rs with complete 7TM topology was obtained from the S1 Data of Policarpo et al. [5]. 425 assemblies were commonly analyzed in both studies. The number of TAS2R genes was almost same between the two studies. (PDF) [file pgen.1011533.s001.pdf]

**Model 1: 7 shifts**

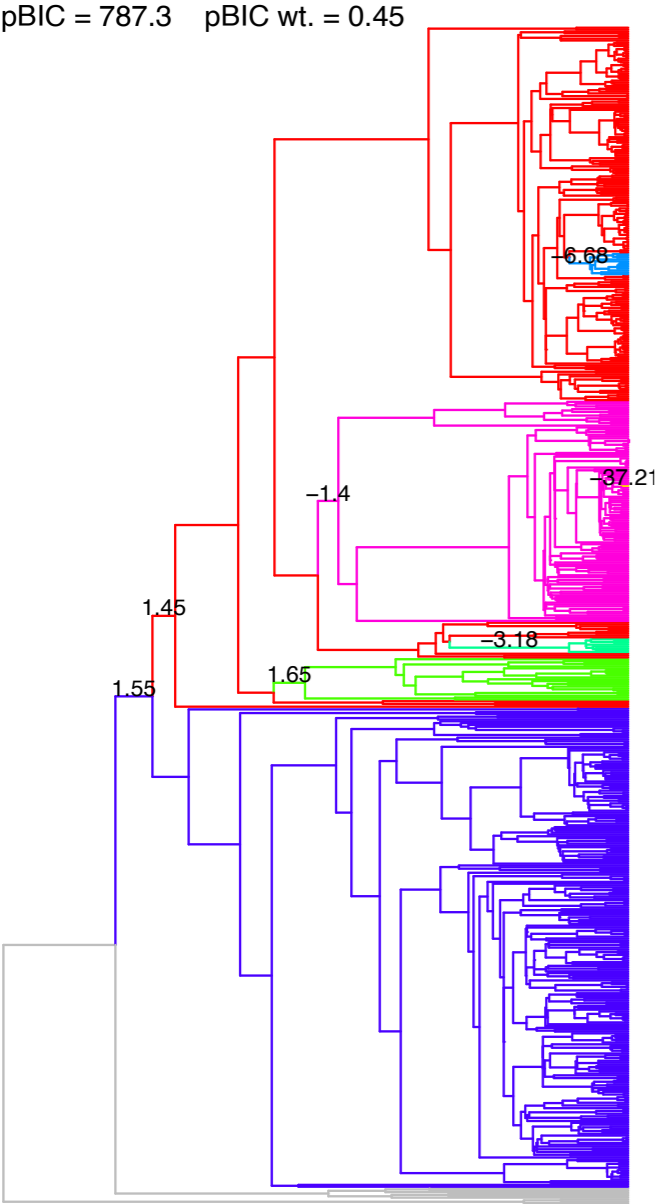

**Model 2: 6 shifts**

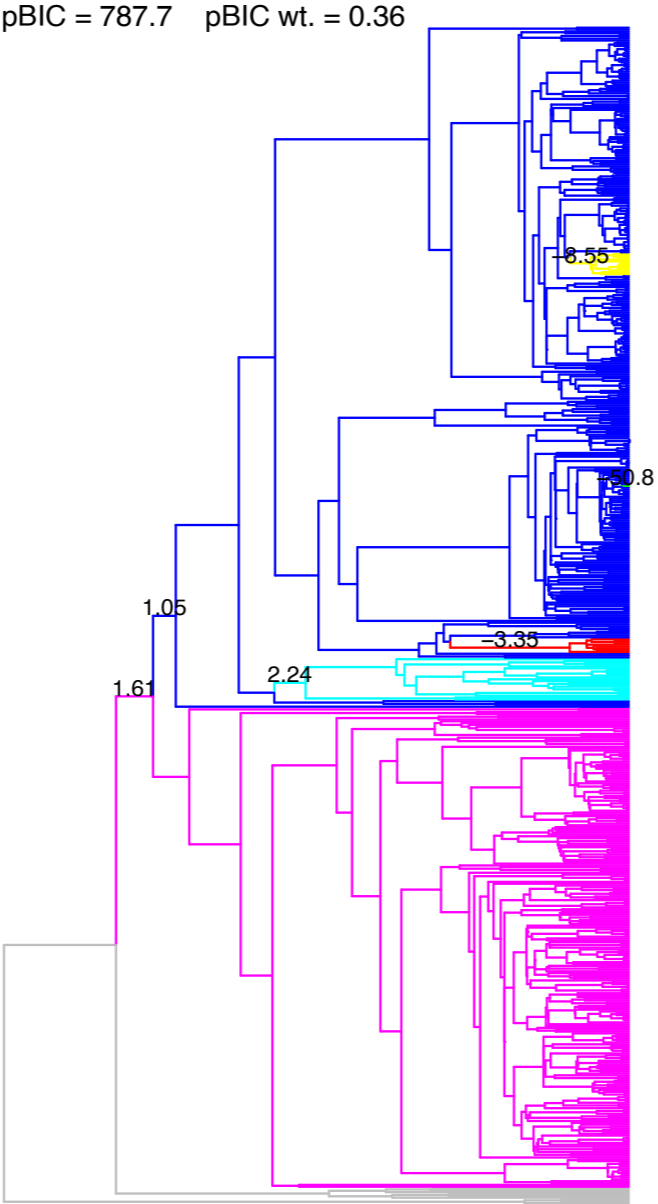

**Model 3: 8 shifts**

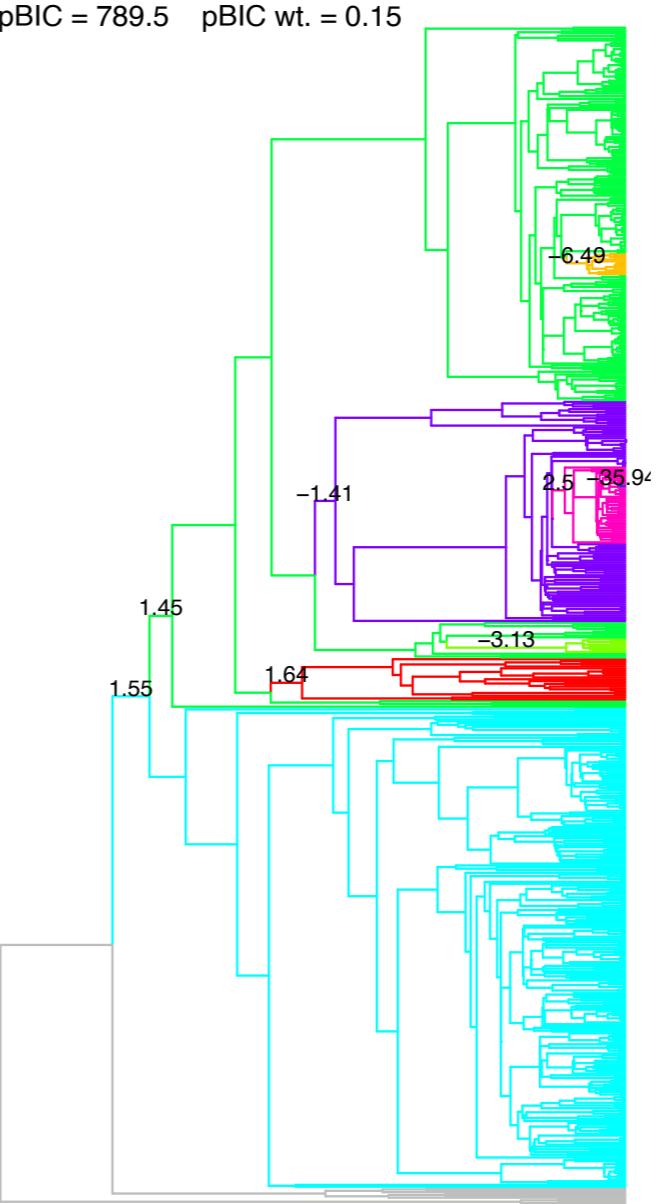

**Model 4: 8 shifts**

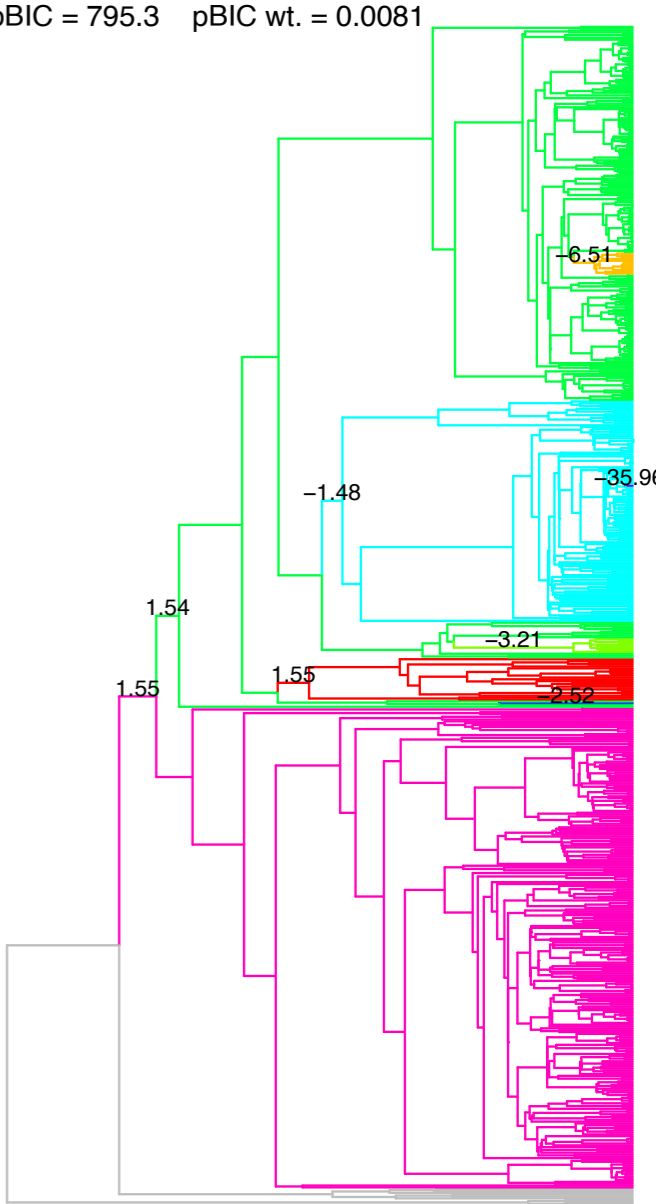

Supplement: S3 Fig — Branches are colored by evolutionary regime, and numbers on branches represent the magnitude of the optimum shift (fold-change) for each regime. pBIC weights are labeled above each plot. (PDF) [file pgen.1011533.s003.pdf]

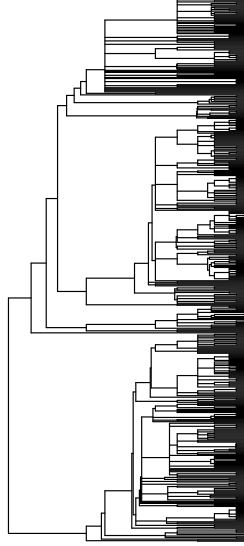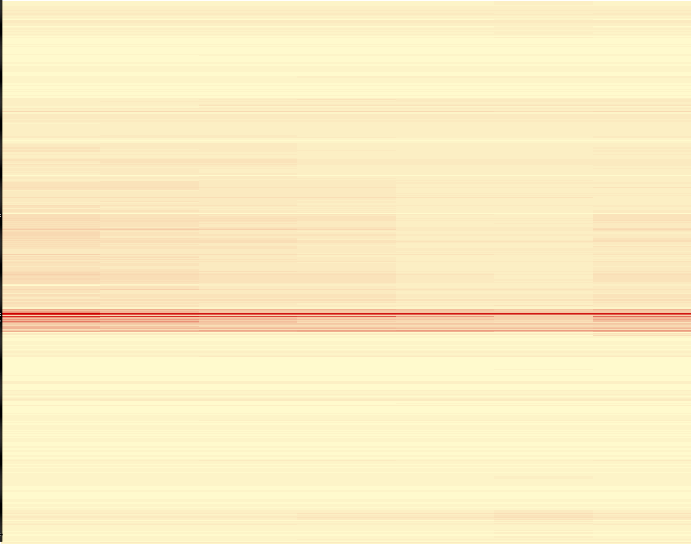

100K

200K

500K

1M

2M

5M

Median

Supplement: S4 Fig — On the right is the underlying species tree, as described in Fig 1A. Heatmap shows the number of clusters identified in each species. For columns 100 K, 200 K, 500 K, 1 M, and 2 M, and 5 M, the name reflects the maximum allowed distance between two neighboring genes within the same cluster. “Median” refers to a more complex analysis described in the Methods section. (PDF) [file pgen.1011533.s004.pdf]

A

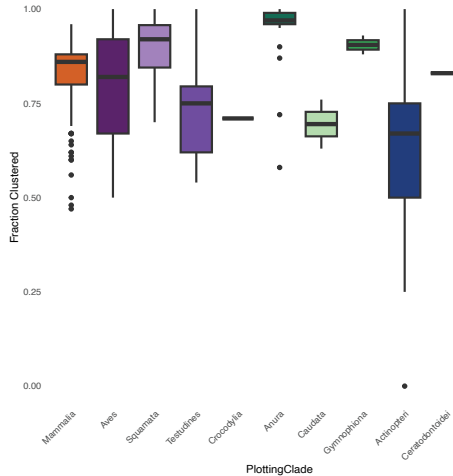

B

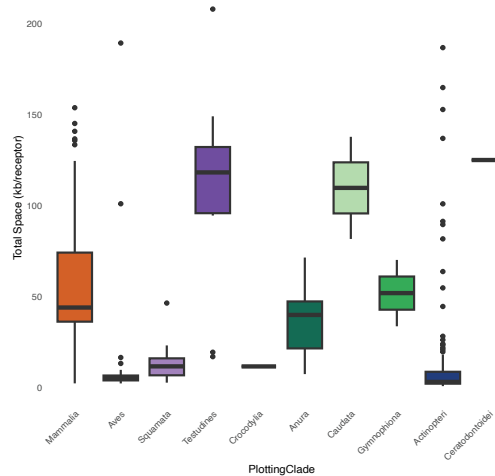

Supplement: S5 Fig — (A) Boxplot showing fraction of all TAS2Rs that are clustered, showing only species that contain TAS2Rs. (B) Boxplot showing average kb per TAS2R within TAS2R clusters, showing only species that contain clusters. (PDF) [file pgen.1011533.s005.pdf]

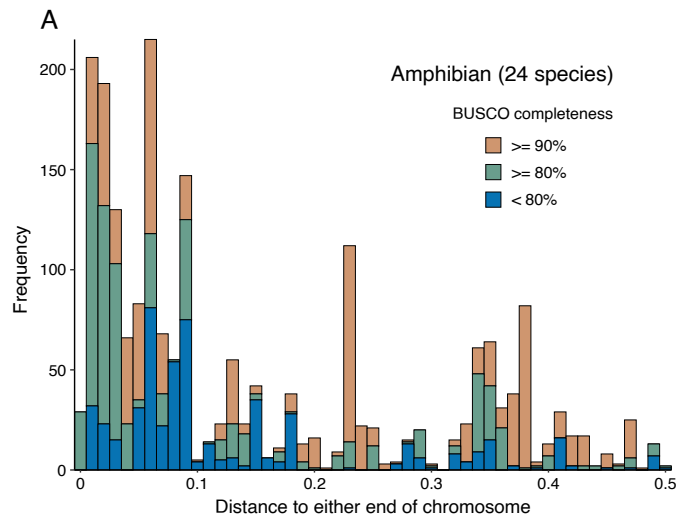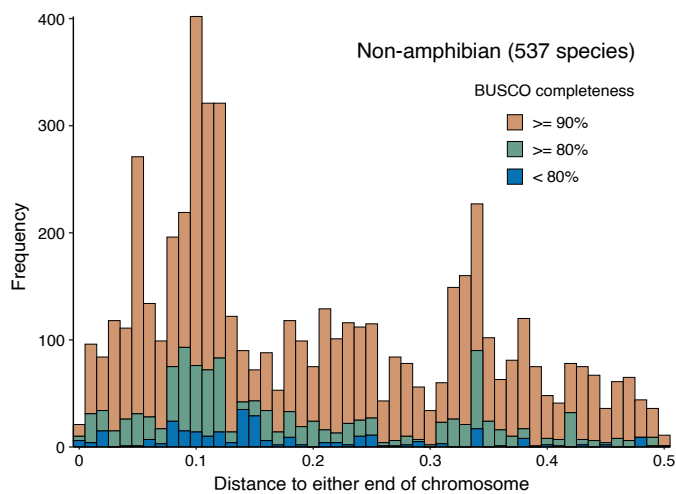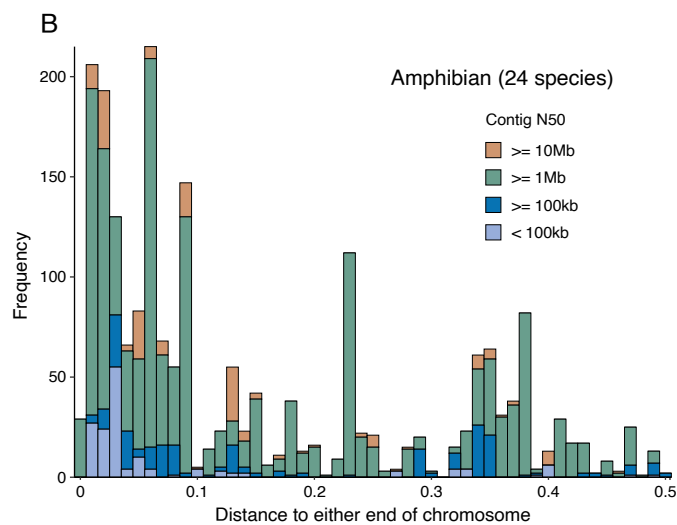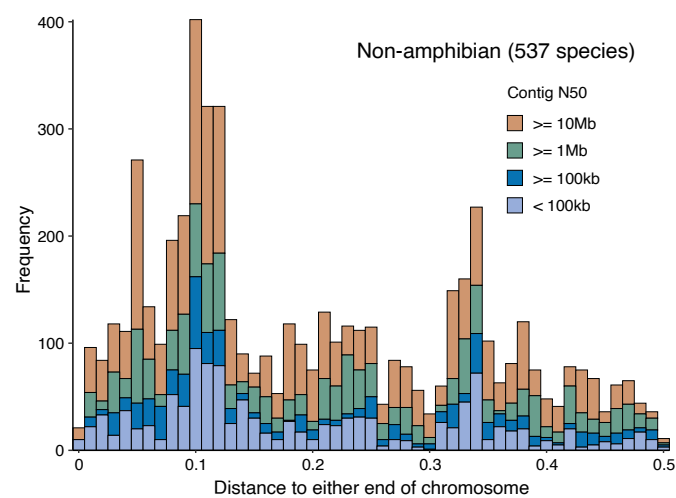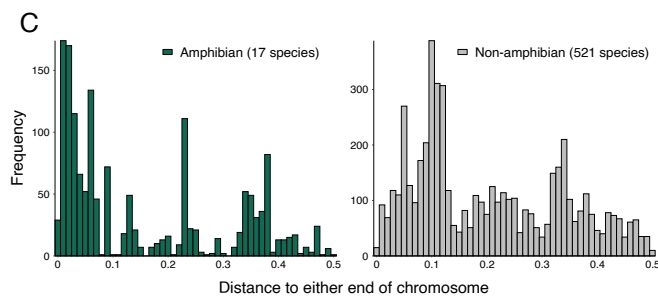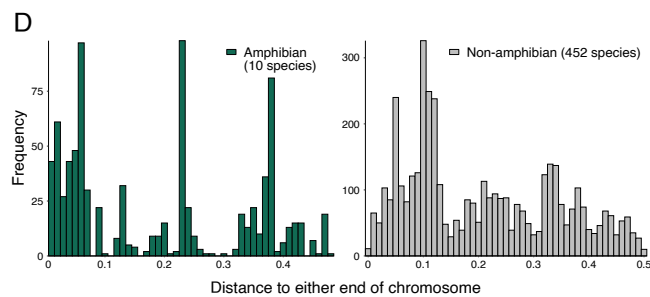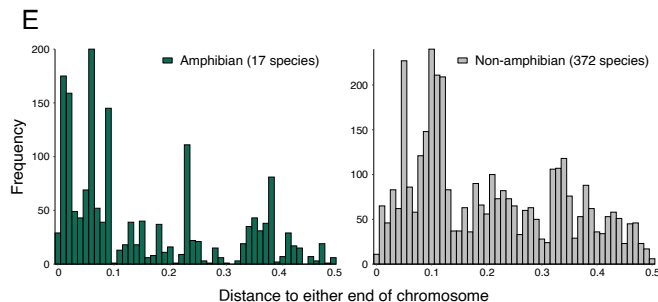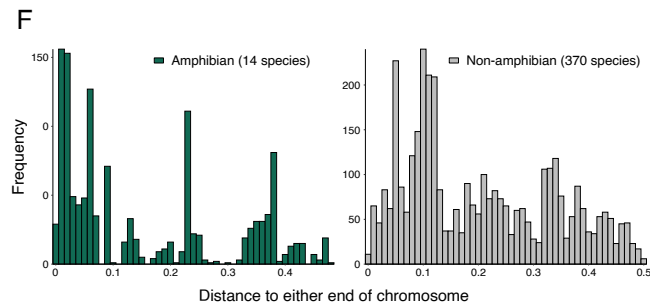

Supplement: S6 Fig — (A-B) Distance to the nearest chromosome end of TAS2Rs in amphibians (left) and nonamphibians (right), corresponding to Fig 2E, colored by (A) BUSCO completeness score, and (B) contig N50. (C-F) Distance to the nearest chromosome end of TAS2Rs in amphibians (left) and non-amphibians (right) from the genome assemblies with (C) over 80% BUSCO completeness (BUSCO80)(D) over 90% BUSCO completeness (BUSCO90)(E) over 1 Mb contig N50, and (F) over 80% BUSCO completeness and over 1 Mb contig N50. Along the x-axis, 0 represents either end and 0.5 is the numerical center of the chromosome. The distance to the nearest chromosome end of TAS2Rs is significantly smaller in amphibians than non-amphibians in all conditions (Welch’s two-sample t-test(C): t = −11.88, df = 2378.9, p = 5.98e-32; (D): t = −3.04, df = 1150.5, p = 0.0012; (E): t = −13.59, df = 3202.9, p = 2.98e-41; (F): t = −10.73, df = 2109.0, p = 1.73e-26). (PDF) [file pgen.1011533.s006.pdf]

### Repeat elements in amphibians

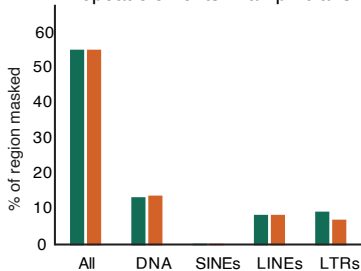

### Repeat elements in non-amphibians

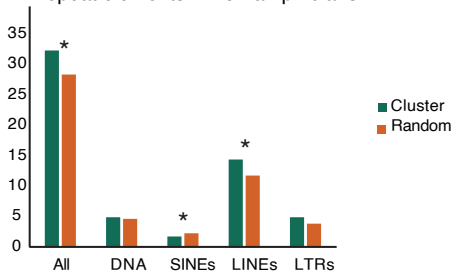

Supplement: S7 Fig — “Cluster” refers to the 100 KB immediately upstream and downstream from each TAS2R cluster in each lineage. “Random” refers to randomly selected 100 KB regions of the genome. Data shown for 15 amphibian species in comparison to 12 non-amphibian species. Comparisons marked with an asterisk (*) are significantly different with p<0.05 in a one-sided paired t-test. (PDF) [file pgen.1011533.s007.pdf]

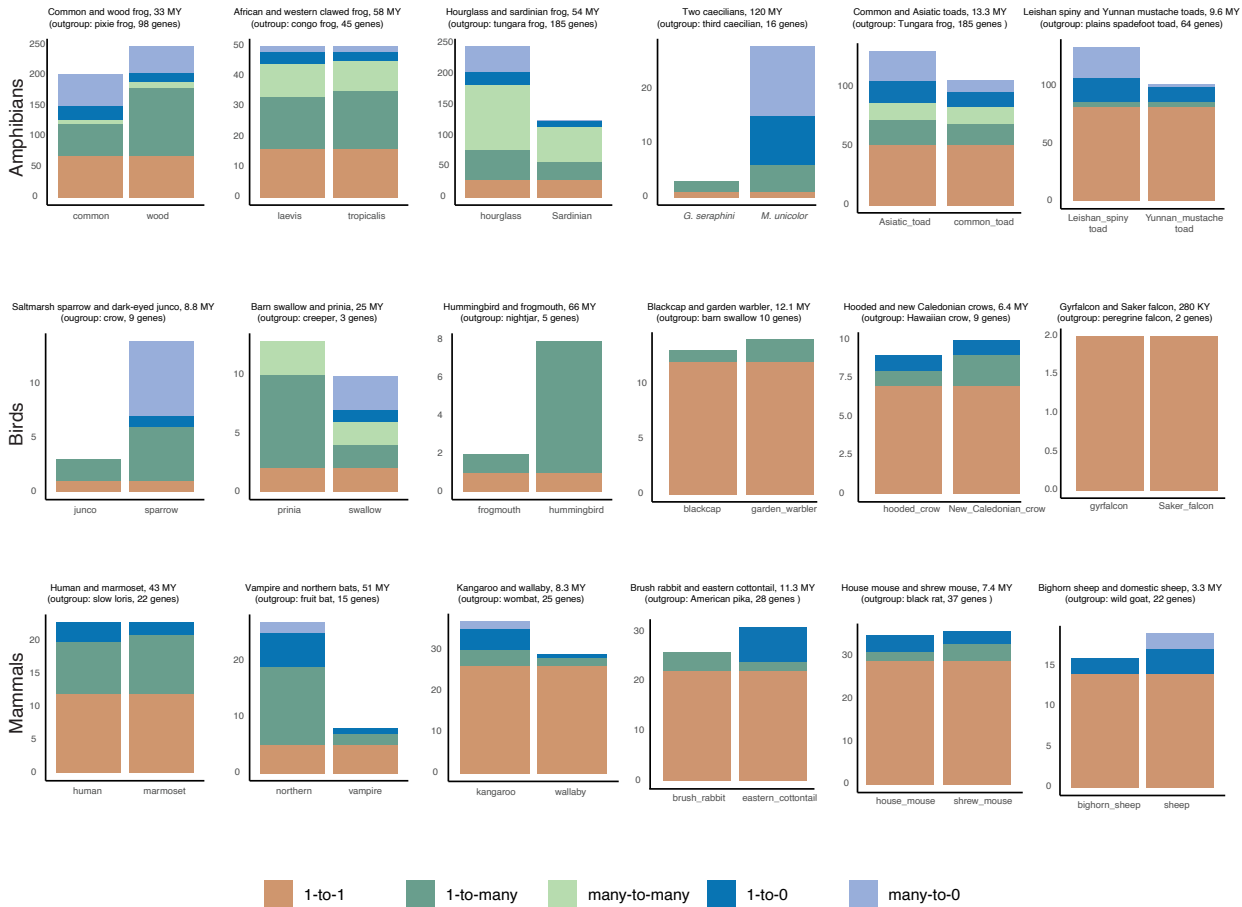

Supplement: S8 Fig — (PDF) [file pgen.1011533.s008.pdf]

Proportion of copy number constrained orthologs by taxa

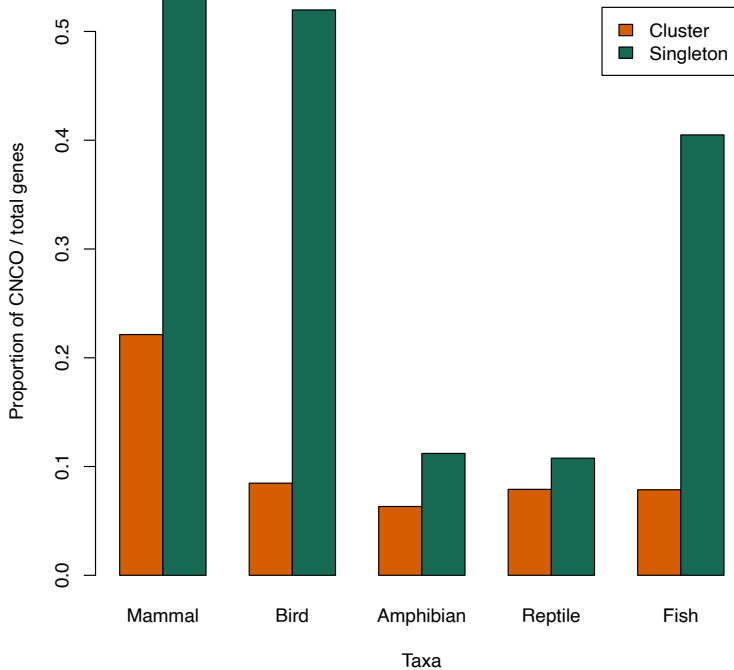

Supplement: S9 Fig — As a brief recap of the text, these genes are present in exactly one copy in greater than 50% of species and are duplicated in fewer than 5% of species. (PDF) [file pgen.1011533.s009.pdf]

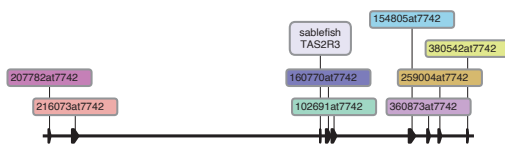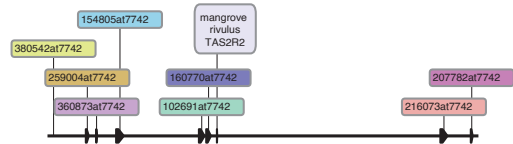

Supplement: S10 Fig — Conserved BUSCO genes are shown in bright colors, matching between the two panels. (PDF) [file pgen.1011533.s010.pdf]

Common Frog  
(*Rana temporaria*)

Wood Frog  
(*Lithobates sylvaticus*)

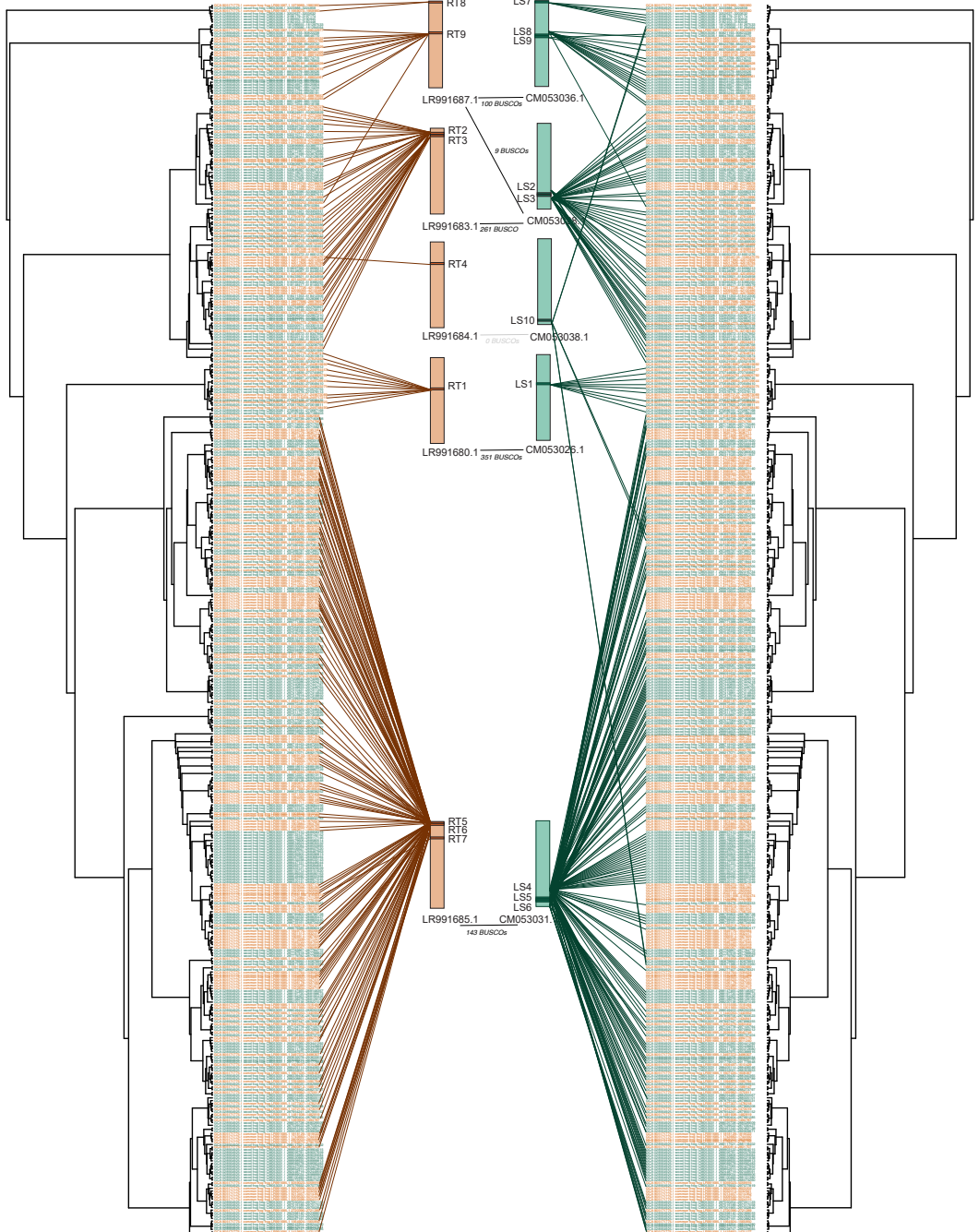

Supplement: S11 Fig — Genes from the common frog (Rana temporaria) are shown in amber on the left and genes from the wood frog (Lithobates sylvaticus) are shown in forest green on the right. Gene location along the chromosome is sometimes accentuated to distinguish very close clusters but is accurate within about 5% of the chromosomal length. (PDF) [file pgen.1011533.s011.pdf]

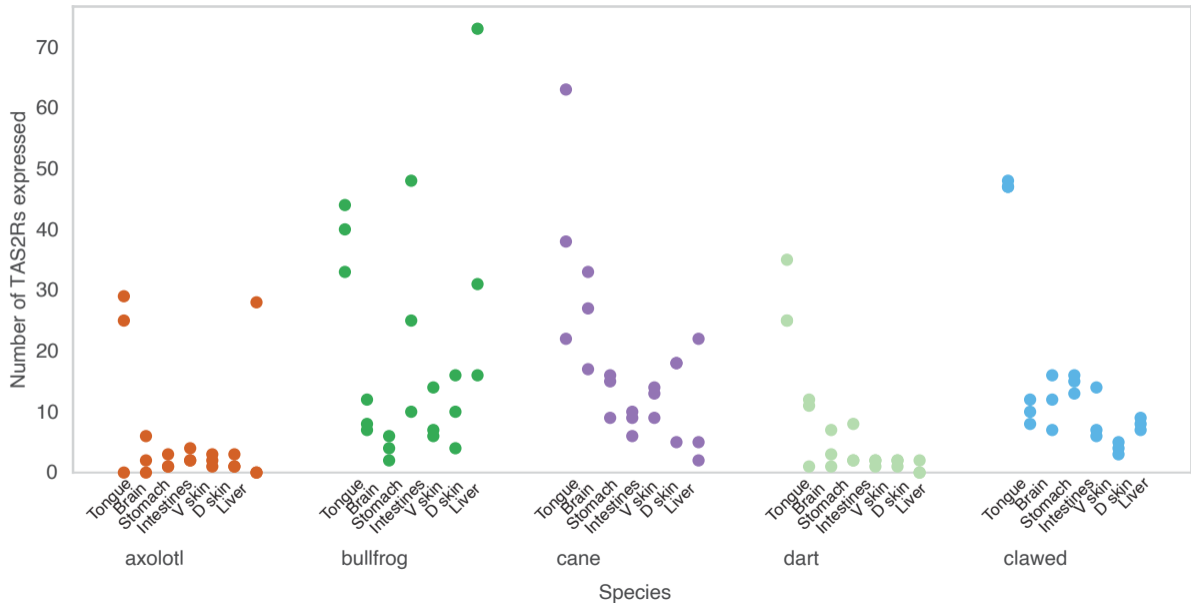

Supplement: S12 Fig — (PDF) [file pgen.1011533.s012.pdf]

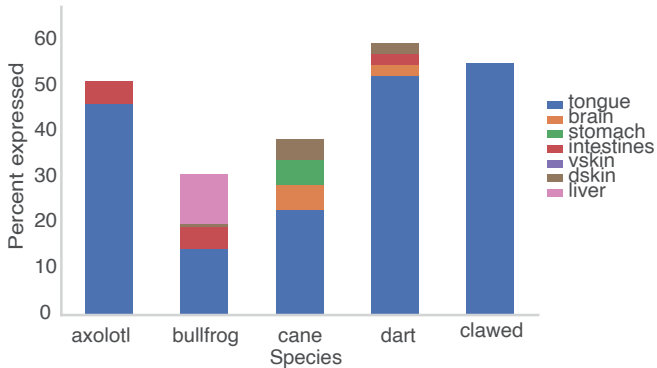

Supplement: S13 Fig — (PDF) [file pgen.1011533.s013.pdf]

Ectodermal: brain, skin

Endodermal: intestines, liver, stomach

Mixed: tongue (taste buds endo)

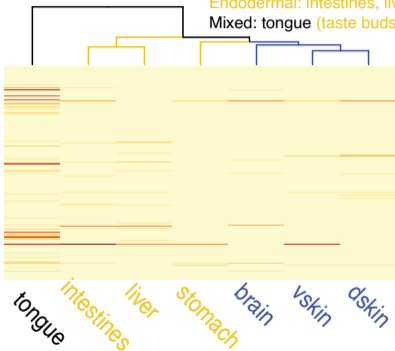

Supplement: S14 Fig — Dendrogram colored according to standard evolutionary biology colors for ectoderm (blue) and endoderm (yellow), with tissues with mixed lineages in black. (PDF) [file pgen.1011533.s014.pdf]

-2.3 value 1.05

Tongue Brain Stomach Intestines Ventral skin Dorsal skin Liver

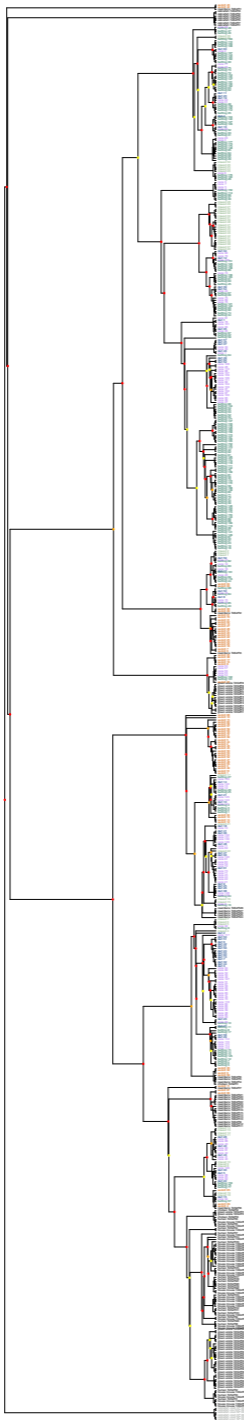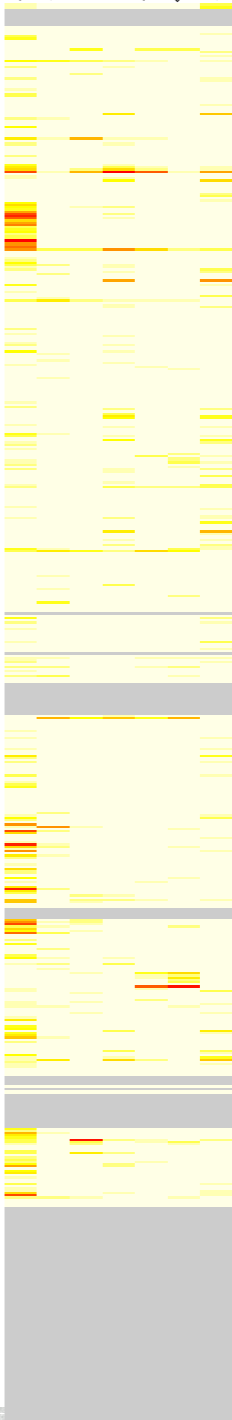

Supplement: S15 Fig — A phylogenetic tree showing the relationship between amphibian sequences based on an amino acid alignment, next to a heatmap showing the expression of each receptor in seven different tissues. Alternate versions of this diagram are available in S17 and S18 Figs. (PDF) [file pgen.1011533.s015.pdf]

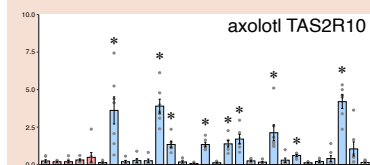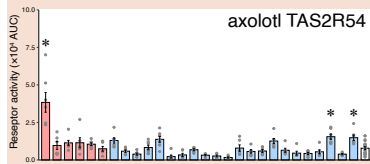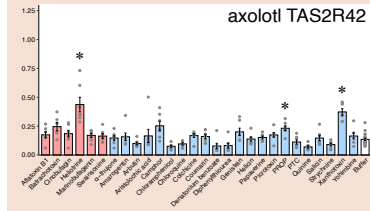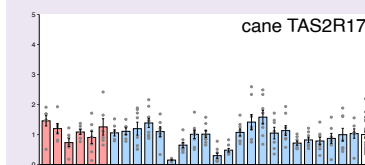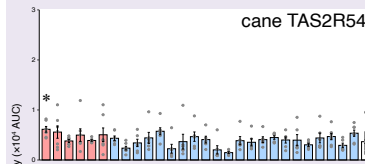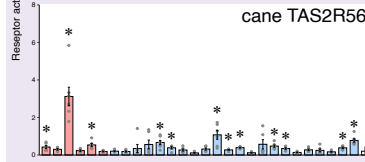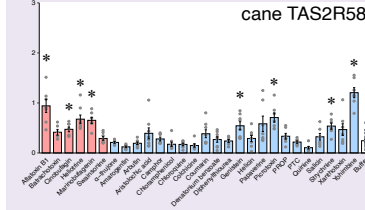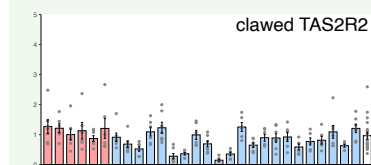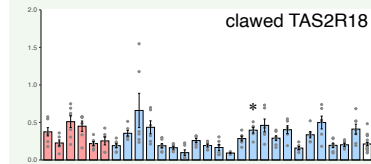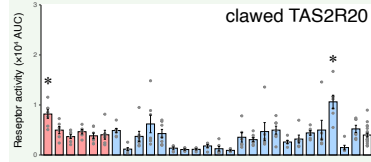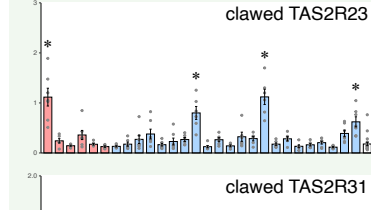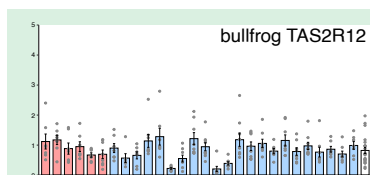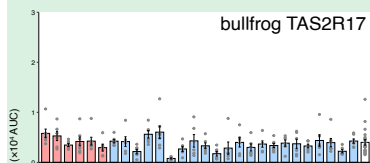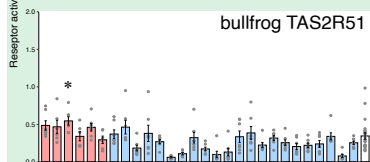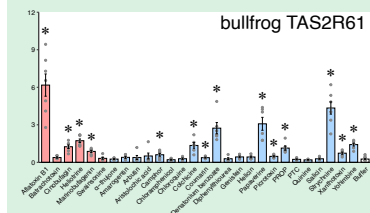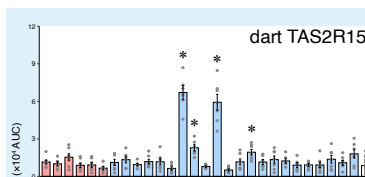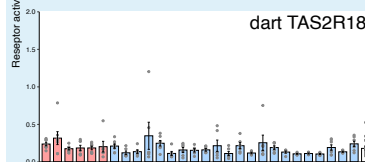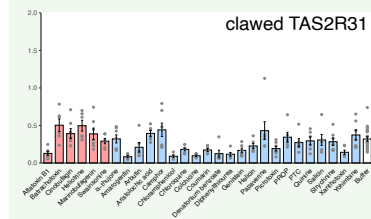

Supplement: S16 Fig — The responses to 6 substances potentially relevant to amphibian ecology, 22 classic bitterants, and assay buffer were represented in pink, light blue, and white, respectively (n = 6–9 for chemicals, n = 21–25 for assay buffer). Asterisks indicate significantly higher responses compared to the response to the assay buffer (two-tailed Welch’s t-test with Benjamini-Hochberg correction, a = 0.05). (PDF) [file pgen.1011533.s016.pdf]

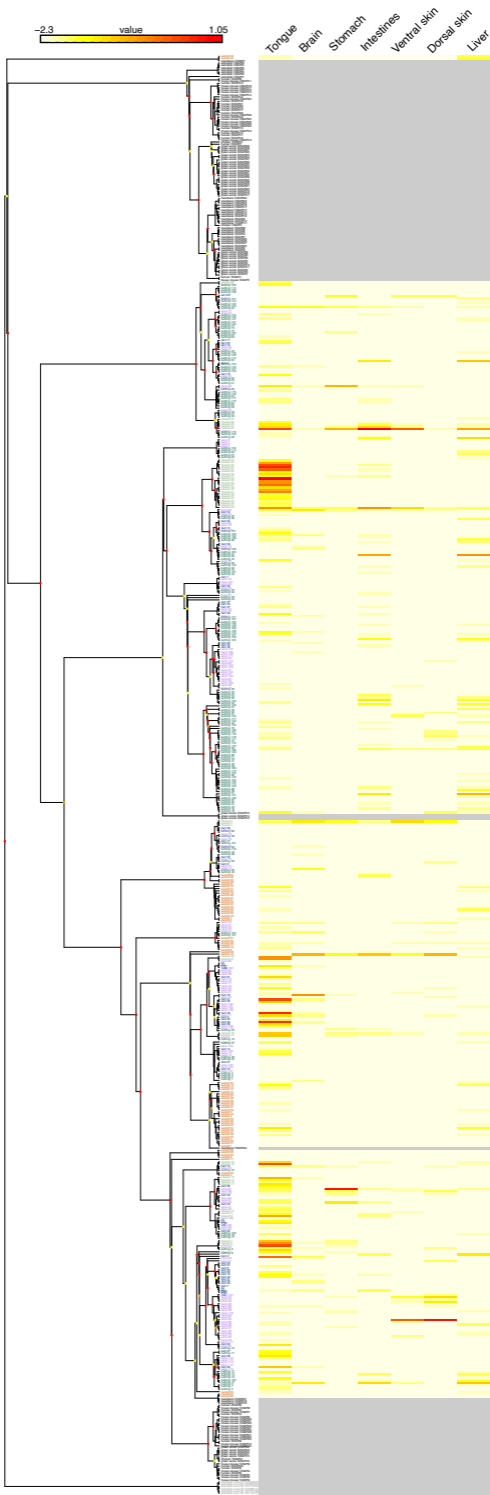

Supplement: S17 Fig — Phylogenetic tree showing relationship between amphibian sequences based on a nucleic acid alignment, next to a heatmap showing the expression of each receptor in seven different tissues. After this diagram was found to be qualitatively similar to S15 Fig, S15 Fig was used in all subsequent analyses. Alternate versions of this diagram are available in S15 and S18 Figs. (PDF) [file pgen.1011533.s017.pdf]

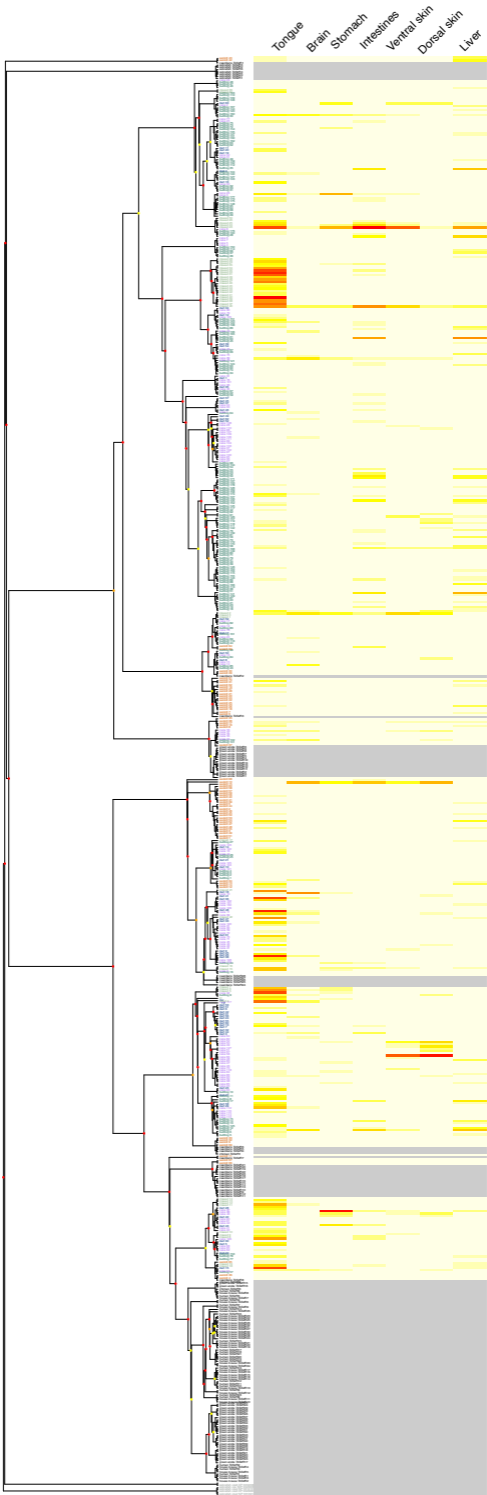

Supplement: S18 Fig — Phylogenetic tree showing relationship between amphibian sequences based on an amino acid alignment, next to a heatmap showing the expression of each receptor in seven different tissues with multi-mapped reads. After this diagram was found to be qualitatively similar to S15 Fig, S15 Fig was used in all subsequent analyses. Alternate versions of this diagram are available in S15 and S17 Figs. (PDF) [file pgen.1011533.s018.pdf]

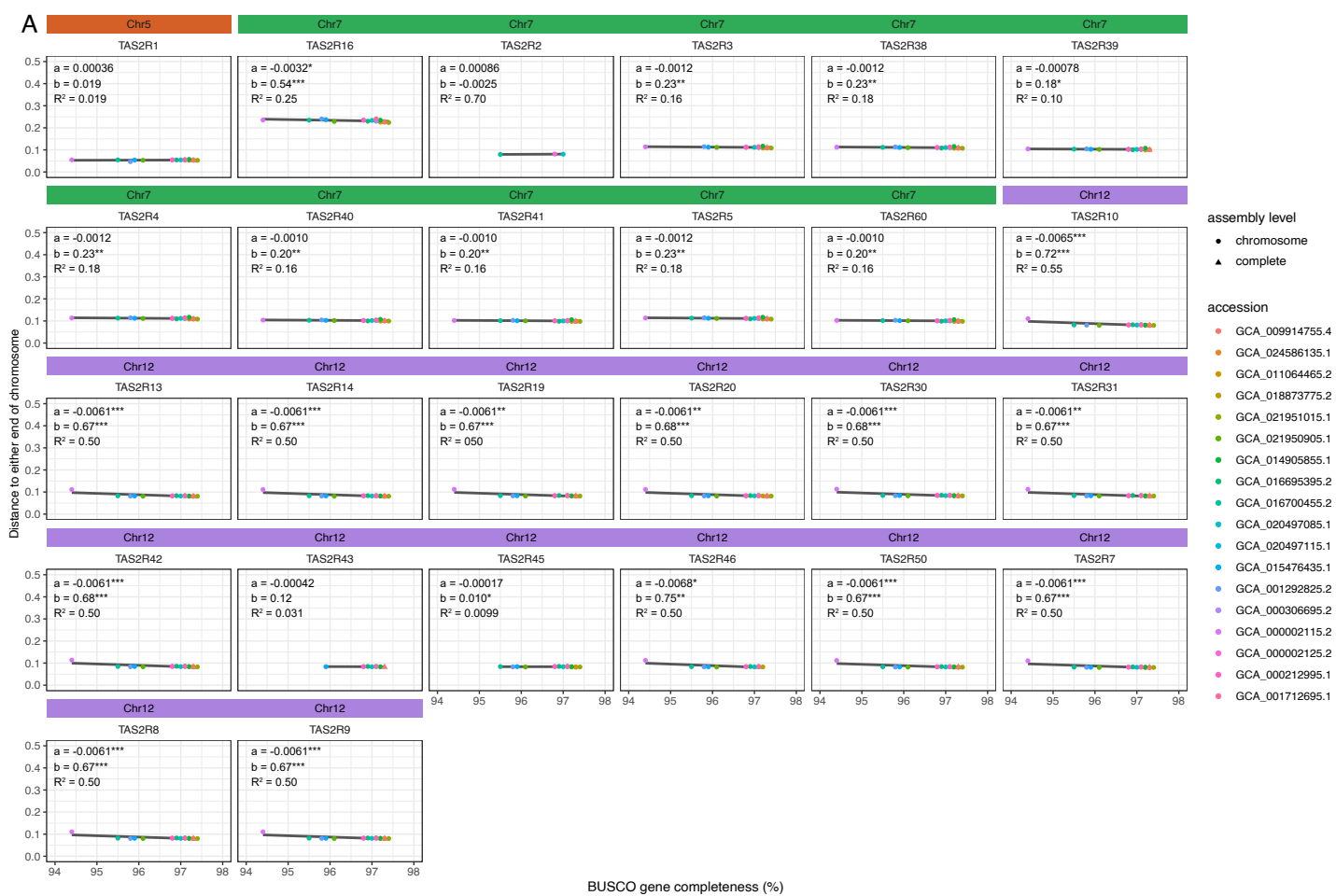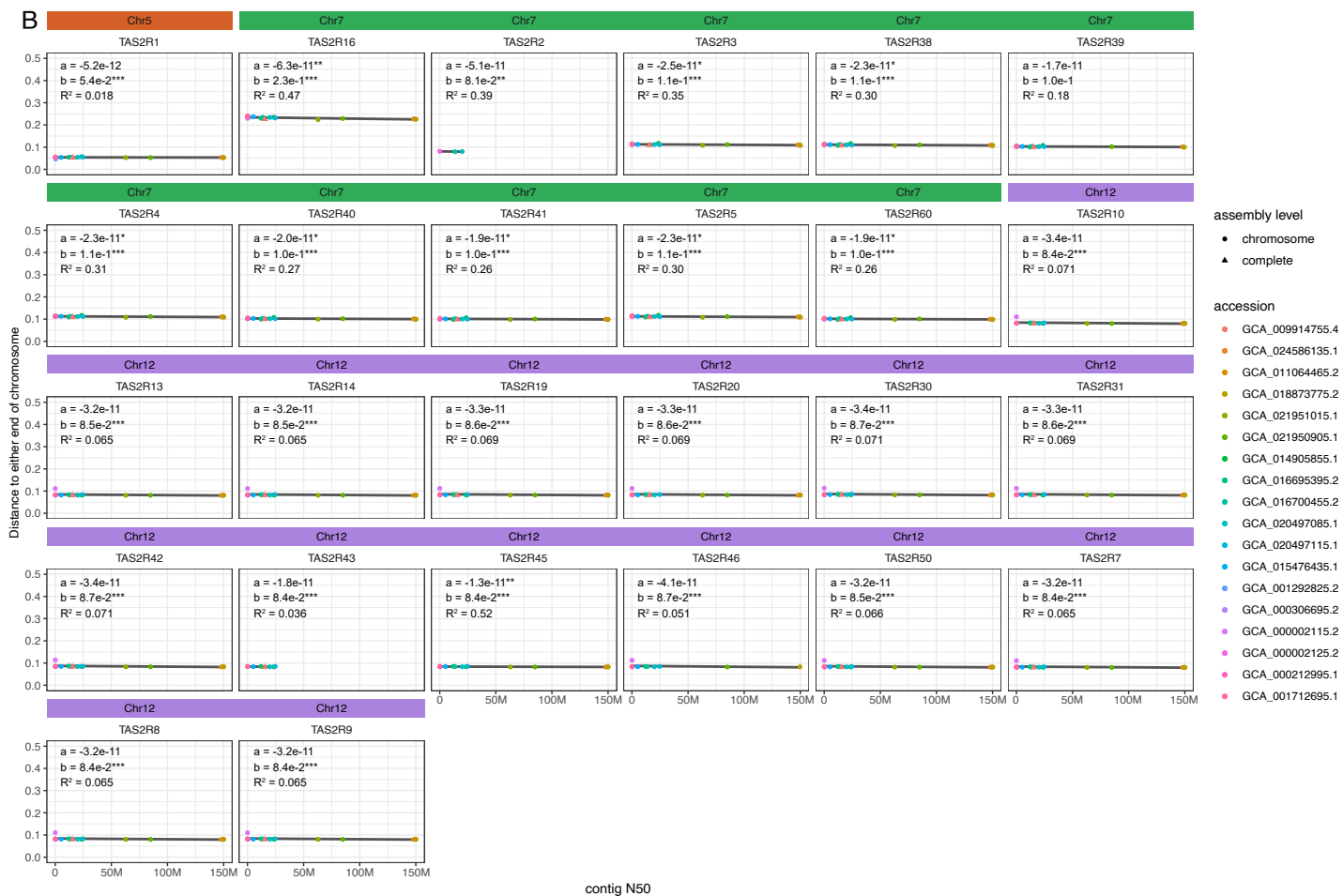

Supplement: S19 Fig — The scatter plots show relationships between TAS2R positions in human genome assemblies and (A) BUSCO completeness and (B) contig N50. Inset numbers indicate R-squared and coefficients of linear regression models (y = ax+b), where x and y indicate BUSCO_score/contig_N50 and gene_position, respectively. P-values represented as * (p < 0.05), ** (p < 0.01), and *** (p < 0.001). (PDF) [file pgen.1011533.s019.pdf]

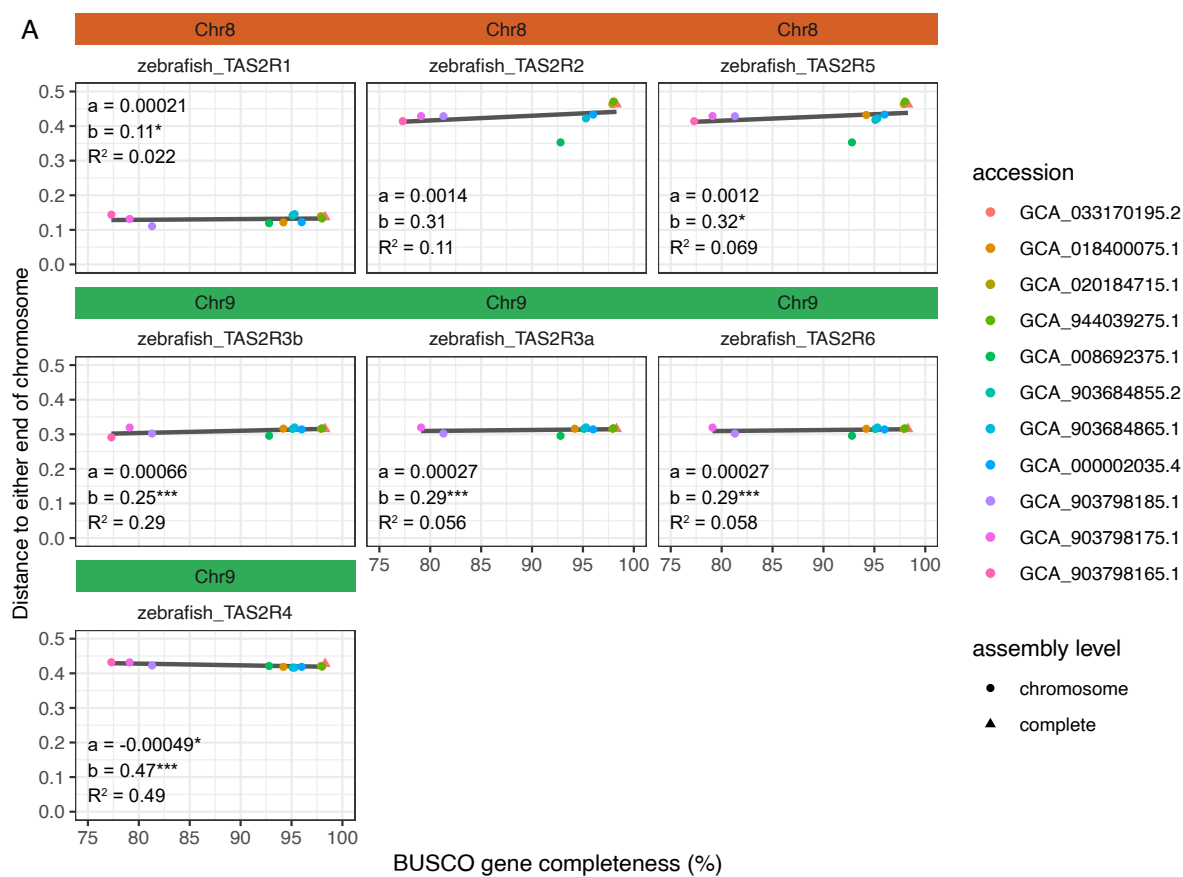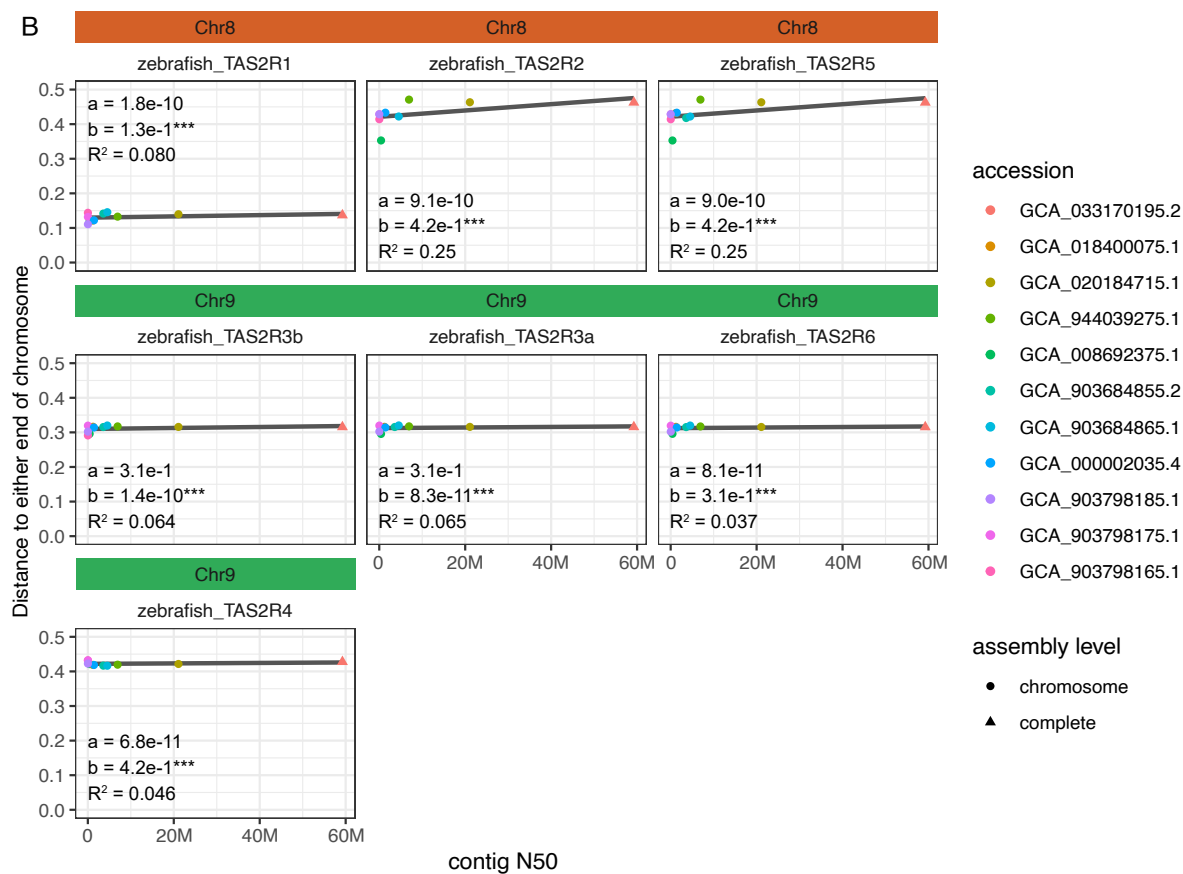

Supplement: S20 Fig — The scatter plots show relationships between TAS2R positions in zebrafish genome assemblies and (A) BUSCO completeness and (B) contig N50. Inset numbers indicate R-squared and coefficients of linear regression models (y = ax + b), where x and y indicate BUSCO_score/contig_N50 and gene_position, respectively. P-values represented as * (p < 0.05), ** (p < 0.01), and *** (p <0.001). (PDF) [file pgen.1011533.s020.pdf]

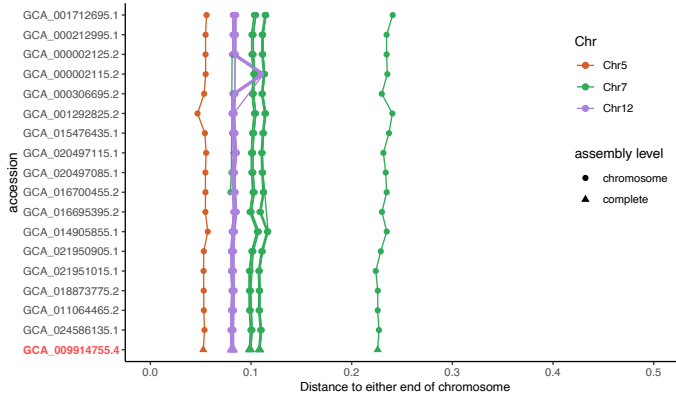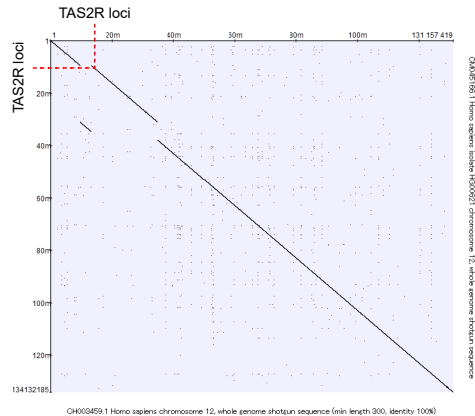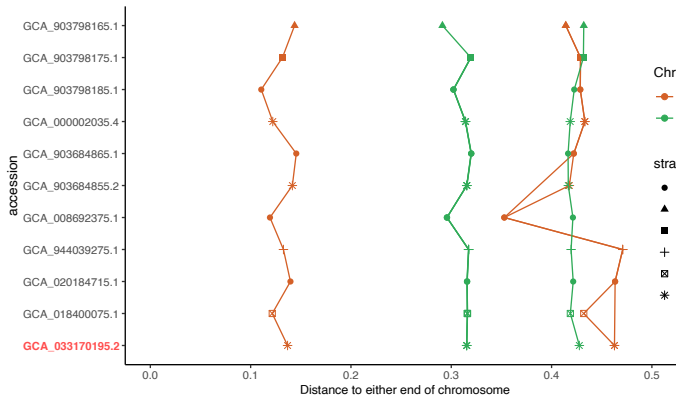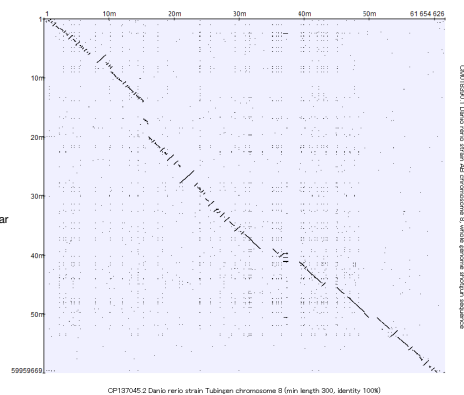

Supplement: S21 Fig — TAS2R loci were compared among chromosome-scale assembly of human (top left) and zebrafish (bottom left) genomes with various qualities (T2T assembly in red). The chromosome 12 in the assembly GCA_00002115.2 had a structural variation between a chromosomal end and TAS2R loci, which affected relative positions of TAS2R loci in the chromosome 12 (top right). In the chromosome 8 of GCA_008692375.1, there were many inversions possibly due to the incorrect assembling, which may have influenced positions of some genes (bottom right). Dot plots were generated by Unipro UGENE software. (PDF) [file pgen.1011533.s021.pdf]

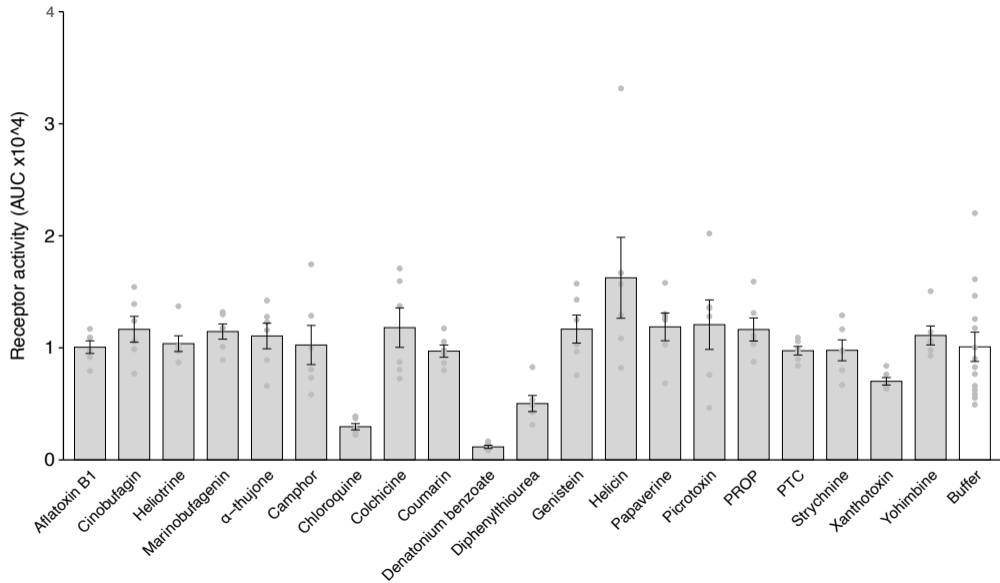

Supplement: S23 Fig — No-receptor control was assayed using luminescence-based functional assays with the substances to which at least one receptor responded (20). The responses to substances and assay buffer were represented in gray and white, respectively (n = 6 for chemicals, n = 14 for assay buffer). There were no significantly higher responses compared to the response to the assay buffer (two-tailed Welch’s t-test with Benjamini-Hochberg correction, α = 0.05). (PDF) [file pgen.1011533.s023.pdf]
