# Supplementary material for: Rapid expansion and specialization of the TAS2R bitter taste receptor family in amphibians
Source: PLoS Genet. 2025 Jan 31;21(1):e1011533. doi: 10.1371/journal.pgen.1011533 (PMC11798467; doi:10.1371/journal.pgen.1011533)
Supplement: S2 Fig — Data shown separately for amphibians alone in the right panel. (PDF) [file pgen.1011533.s002.pdf]

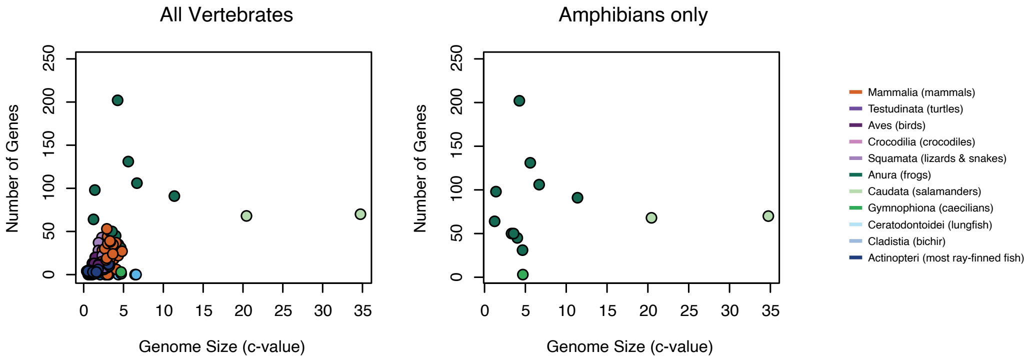

All Vertebrates
Amphibians only
Number of Genes
Genome Size (c-value)
0
50
100
150
200
250
0
5
10
15
20
25
30
35
Mammalia (mammals)
Testudinata (turtles)
Aves (birds)
Crocodilia (crocodiles)
Squamata (lizards & snakes)
Anura (frogs)
Caudata (salamanders)
Gymnophiona (caecilians)
Ceratodontoidei (lungfish)
Cladistia (bichir)
Actinopteri (most ray-finned fish)
