# Supplementary material for: Rapid expansion and specialization of the TAS2R bitter taste receptor family in amphibians
Source: PLoS Genet. 2025 Jan 31;21(1):e1011533. doi: 10.1371/journal.pgen.1011533 (PMC11798467; doi:10.1371/journal.pgen.1011533)
Supplement: S22 Fig — For axolotl and bullfrog, note that certain samples were of poor quality (based on RIN scores) or were briefly frozen, as indicated with different shapes. For cane, dart, and clawed samples, all passed the RIN score threshold and only fresh samples were used. (PDF) [file pgen.1011533.s022.pdf]

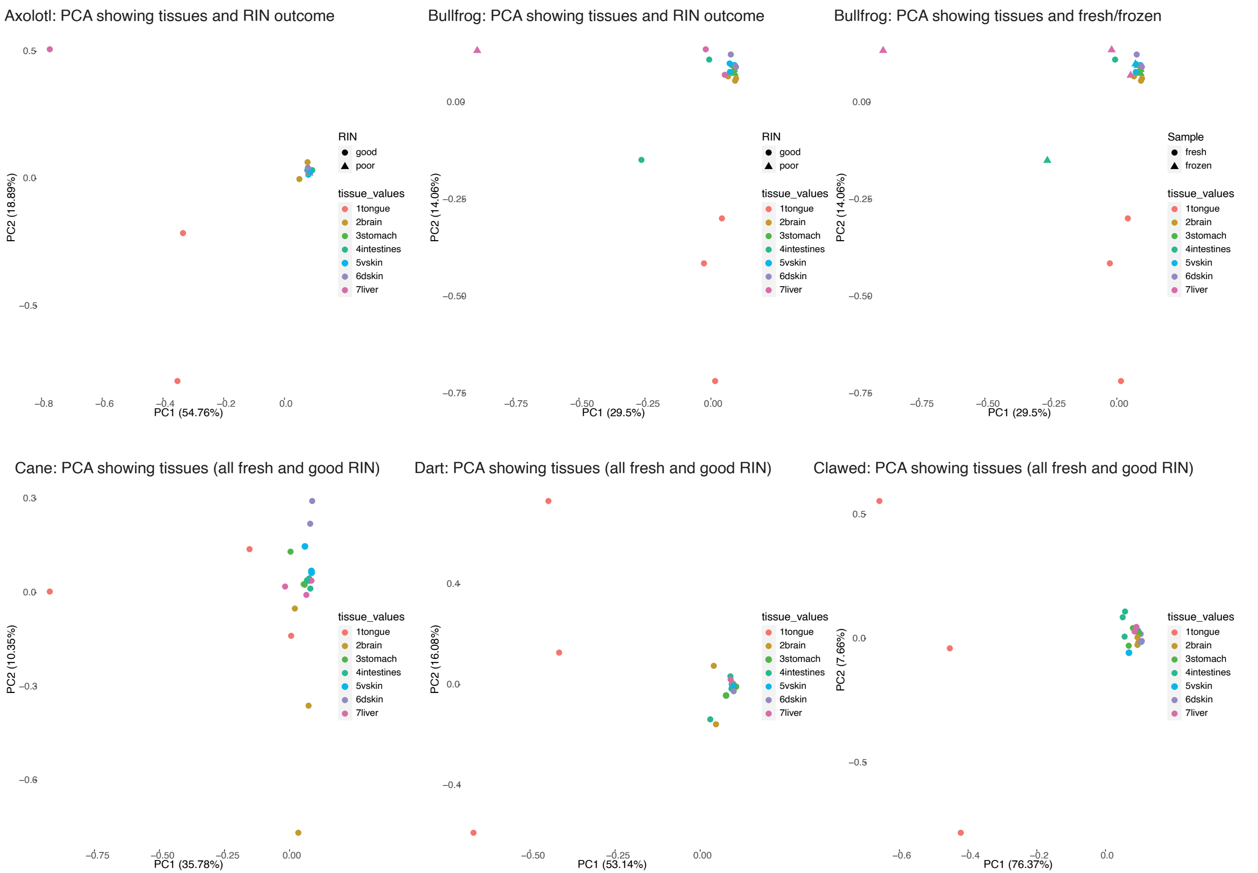

Axolotl: PCA showing tissues and RIN outcome
Bullfrog: PCA showing tissues and RIN outcome
Bullfrog: PCA showing tissues and fresh/frozen
PC1 (54.76%)
PC2 (18.89%)
PC1 (29.5%)
PC2 (14.06%)
RIN
good
poor
Sample
fresh
frozen
tissue_values
1tongue
2brain
3stomach
4intestines
5vskin
6dskin
7liver
Cane: PCA showing tissues (all fresh and good RIN)
Dart: PCA showing tissues (all fresh and good RIN)
Clawed: PCA showing tissues (all fresh and good RIN)
PC1 (35.78%)
PC2 (10.35%)
PC1 (53.14%)
PC2 (16.08%)
PC1 (76.37%)
PC2 (7.66%)
