# Supplementary material for: Rapid expansion and specialization of the TAS2R bitter taste receptor family in amphibians
Source: PLoS Genet. 2025 Jan 31;21(1):e1011533. doi: 10.1371/journal.pgen.1011533 (PMC11798467; doi:10.1371/journal.pgen.1011533)
Supplement: S1 Table — (PDF) [file pgen.1011533.s024.pdf]

		all		DNA		SINEs		LINEs		LTRs		all minus LINES	
Common name	*Taxa*	*cluster*	*random*	*cluster*	*random*	*cluster*	*random*	*cluster*	*random*	*cluster*	*random*	*cluster*	*random*
Asiatic_toad	Amphibian	68.36	70.68	18.5	20.19	0.17	0.08	6.97	7.01	11.09	7.66	61.39	63.67
Kenyan_clawed_ frog	Amphibian	43.98	35.9	19.01	19.82	0.02	0.09	7.64	4.06	8.09	3.89	36.34	31.84
Leishan_spiny _toad	Amphibian	60.09	70.32	14.8	18.06	0.13	0.14	8.14	15.78	13.77	7.06	51.95	54.54
Puerto_Rican _coqui	Amphibian	54.77	48.16	11.05	7.08	0.12	0.27	10.29	4.17	4.52	4.16	44.48	43.99
Sardinian_treefrog	Amphibian	49.38	63.55	9.53	19.64	0	0.01	4.17	7.24	27.27	11.68	45.21	56.31
Tungara_frog	Amphibian	60.31	49.02	13.33	8.16	0	0.02	6.44	2.49	6.42	6.21	53.87	46.53
Yunnan_mustache _toad	Amphibian	60.85	63.36	8.94	11.14	0.06	0.05	13.37	11.36	13.21	9.49	47.48	52
caecilians (*Microcaecilia unicolor*)	Amphibian	36.41	38.42	3.1	3.22	0.53	1.03	18.05	22.42	8.17	2.6	18.36	16
common_frog	Amphibian	61.83	65.27	19.28	15.94	1.28	0.17	6.78	7.82	3.08	9.14	55.05	57.45
common_toad	Amphibian	60.12	75.96	14.48	26.09	0.01	0.04	2.06	7.36	9.73	9.94	58.06	68.6
frogs_and_toads (*Pyxicephalus adspersus*)	Amphibian	31.68	31.54	7.46	7.51	0.15	0	3.69	4.16	3.11	1.99	27.99	27.38
hourglass_treefrog	Amphibian	63.43	51.4	13.39	10.06	0	0.02	10.43	7.05	1.2	4.04	53	44.35
plateau_brown _frog	Amphibian	59.75	66.98	15.57	16.56	0.62	0.43	7.2	6.43	4.71	8.17	52.55	60.55
tropical_clawed_ frog	Amphibian	41.63	28.85	25.38	18.84	0.28	0	3.6	3.05	0.5	0.67	38.03	25.8
two-lined_caecilian	Amphibian	71.56	64.06	7.53	6	0.45	0.56	16.2	18.59	24.08	19.03	55.36	45.47
