# Supplementary material for: Rapid expansion and specialization of the TAS2R bitter taste receptor family in amphibians
Source: PLoS Genet. 2025 Jan 31;21(1):e1011533. doi: 10.1371/journal.pgen.1011533 (PMC11798467; doi:10.1371/journal.pgen.1011533)
Supplement: S2 Table — (PDF) [file pgen.1011533.s025.pdf]

		all		DNA		SINEs		LINEs		LTRs		all minus LINES	
Common name	***Taxa***	*cluster*	*random*	*cluster*	*random*	*cluster*	*random*	*cluster*	*random*	*cluster*	*random*	*cluster*	*random*
Aeolian_wall_lizard	Reptile	49.72	49.11	6.1	7.65	3.72	6.93	22.3	19.66	3.31	1.87	27.42	29.45
Common_starling	Bird	17	4.72	0.05	0.04	0	0.01	4.94	2.88	7.77	0.63	12.06	1.84
Malagasy_flying _fox	Mammal	25.82	20.98	2.75	1.6	0.7	1.89	16.51	11.92	4.4	3.72	9.31	9.06
North_American_ porcupine	Mammal	35.01	29.78	1.82	1.64	1.53	3.95	21	14.14	8.7	7.49	14.01	15.64
blackcap	Bird	8.67	1.88	0.1	0	0	0	4.75	0.15	2.26	0.57	3.92	1.73
Diamondback _terrapin	Reptile	51.58	39.29	14.05	11.82	2.63	1.68	15.11	12.42	12.35	7.27	36.47	26.87
greater_Indian_ rhinoceros	Mammal	32.33	31.55	2.59	2.77	2.36	2.93	18.32	19.47	7.9	5.12	14.01	12.08
lion	Mammal	27.91	28.26	1.76	4.75	2.88	4.55	17.97	17.38	3.18	0.16	9.94	10.88
prairie_rattlesnake	Reptile	47.51	59.38	9.7	7.78	2.88	2.02	22.7	21.52	0.33	11.24	24.81	37.86
reedfish	Cladistia	64.11	58.47	19.77	15.98	2.62	3.37	12.19	13.78	3.3	4.38	51.92	44.69
rock_pigeon	Bird	16.77	6.97	0.52	0.09	0.06	0.06	12.31	3.67	1.57	0.82	4.46	3.3
yellow-throated _sandgrouse	Bird	9.11	9.7	0.33	0.09	0.09	0.03	4.73	4.53	1.98	3.27	4.38	5.17
