# Supplementary material for: Rapid expansion and specialization of the TAS2R bitter taste receptor family in amphibians
Source: PLoS Genet. 2025 Jan 31;21(1):e1011533. doi: 10.1371/journal.pgen.1011533 (PMC11798467; doi:10.1371/journal.pgen.1011533)
Supplement: S3 Table — (PDF) [file pgen.1011533.s026.pdf]

| | Full_Names | Age | Num_ occurrences |
|---|---|---|---|
| Cluster1 | ['GCA_022829085.1_CM040772.1:18672273_20673211', 'GCA_947034865.1_OX344776.1:11634334_13635242', 'GCA_936440315.1_OW387177.1:17649116_19650039', 'GCA_936440315.1_OW387178.1:16925439_18926506', 'GCA_027475355.1_CM050213.1:20422635_22423708', 'GCA_027475355.1_CM050213.1:11796741_13797643', 'GCA_003368295.1_CM010465.1:14821299_16822195', 'GCA_023724105.1_CM043232.1:15347402_17348322', 'GCA_029620275.1_CM056144.1:15659799_17660722', 'GCA_017589495.2_CM030048.1:14593197_16594123', 'GCA_018492685.1_CM031717.1:26451189_28452115', 'GCA_024489055.1_CM044606.1:24529375_26530301', 'GCA_018340385.1_CM031269.1:12675919_14676827', 'GCA_018340385.1_CM031294.1:17027184_19028107', 'GCA_023376895.1_CM041799.1:20267647_22268570', 'GCA_903798145.1_LR812510.1:23929525_25930442', 'GCA_903798195.1_LR812527.1:27972413_29973330', 'GCA_020184715.1_CM035063.1:23813294_25814211', 'GCA_900700415.2_LR535858.1:13774406_15775353', 'GCA_900700415.2_LR535858.1:14909313_16910251'] | 208.94656 | 20 |
| Cluster2 | ['GCA_022829085.1_CM040780.1:16198224_18199261', 'GCA_003368295.1_QPKE01000946.1:369697_2370866', 'GCA_018340385.1_CM031268.1:14038037_16039002', 'GCA_023724105.1_CM043229.1:12982718_14983695', 'GCA_023724105.1_CM043230.1:15700573_17701538', 'GCA_947034865.1_OX344778.1:12537251_14538228', 'GCA_018340385.1_CM031293.1:14228591_16229556', 'GCA_936440315.1_OW387195.1:13993176_15994285', 'GCA_936440315.1_OW387196.1:13169342_15170304', 'GCA_903798145.1_LR812495.1:22020188_24024241', 'GCA_023376895.1_CM041804.1:18540364_20541326', 'GCA_019155185.1_CM032806.1:8077550_10078536', 'GCA_903798195.1_LR812526.1:37888866_39893018', 'GCA_029620275.1_CM056148.1:13576275_15577240', 'GCA_024489055.1_CM044601.1:17014211_19015248', 'GCA_020184715.1_CM035062.1:32754007_34755002', 'GCA_020184715.1_CM035062.1:32756936_34758003', 'GCA_003368295.1_CM010439.1:17690010_19690975'] | 96.87146 | 18 |
| Cluster3 | ['GCA_001649575.2_CM027009.1:10111468_12112457', 'GCA_022829085.1_CM040780.1:3230761_5231738', 'GCA_026122265.1_CM047507.1:8460960_10461931', 'GCA_947034865.1_OX344778.1:21300598_23305411', 'GCA_949987685.1_OX465146.1:9226381_11227361', 'GCA_904848185.1_LR884464.1:28738130_30739107', 'GCA_013435755.1_CM024202.1:15594399_17595379', 'GCA_936440315.1_OW387196.1:25728352_27729335', 'GCA_019155185.1_CM032806.1:453571_2454542', 'GCA_027123335.1_CM049572.1:15459450_17460430', 'GCA_902713425.2_OV754680.1:6127917_8132772', 'GCA_902713425.2_OV754629.1:5968826_7973609', 'GCA_022539595.1_CM040066.1:15355058_17356038', 'GCA_900634795.4_LR132023.3:15095277_17096443', 'GCA_900700375.2_LR535823.1:9834401_11835375', 'GCA_003047355.2_CM009712.1:15946963_17947943', 'GCA_900324465.3_LR132038.1:4930367_6931350', 'GCA_014839685.1_CM026066.1:9647136_11648122', 'GCA_001904815.2_CM007779.1:20428353_22429327', 'GCA_000972845.2_CM011617.1:219043_2220071', 'GCA_013347855.1_CM023574.1:15060331_17061260', 'GCA_013347855.1_CM023574.1:11805285_13806259', 'GCA_025169545.1_CM045892.1:12215537_14216511', 'GCA_025169545.1_CM045892.1:15499817_17500740', 'GCA_001640805.2_CM044665.1:16306573_18307550', 'GCA_018555375.2_CM031790.1:12113349_14114272', 'GCA_001858045.3_CM007493.2:9095348_11096301', 'GCA_029692045.1_CM056184.1:24801530_26802504', 'GCA_023724105.1_CM043229.1:23739279_25740271', 'GCA_001465895.2_LN609386.1:8197010_10197987', 'GCA_023724105.1_CM043230.1:3793980_5794963', 'GCA_902810595.1_LR778268.1:21283034_23283999', 'GCA_003999625.1_CM012445.1:22140943_24141917', 'GCA_011952105.1_CM022476.1:22758592_24759569', 'GCA_027596085.2_CM053351.1:17253318_19254295', 'GCA_002234675.1_CP020673.1:19848938_21849915', 'GCA_002922805.2_CM022587.1:25771103_27772077', 'GCA_001444195.3_CM010737.1:24768742_26769719', 'GCA_003331165.2_CM010386.2:24864894_26865871', 'GCA_002775205.2_CM008949.1:25727823_27728800', 'GCA_029620275.1_CM056148.1:25992257_27993240', 'GCA_000242695.1_CM001423.1:8783074_10784207', 'GCA_027744805.2_CM050981.1:20705701_22706684', 'GCA_900963305.2_LR584050.1:5054849_7055832', 'GCA_023566465.1_CM042218.1:9884570_11885544', 'GCA_013358685.1_CM023608.1:25789835_27790812', 'GCA_023856365.1_CM043636.1:3522064_5523020', 'GCA_020698575.1_CM036249.1:25340832_27341818', 'GCA_023856365.1_CM043636.1:8878681_10879655', 'GCA_022829145.1_CM040744.1:27658136_29661041', 'GCA_022829145.1_CM040744.1:33581268_35582242', 'GCA_021346845.1_CM037929.1:10920410_12921381', 'GCA_017639675.1_CM030179.1:10007217_12014260', 'GCA_017639675.1_CM030185.1:12916042_14917043', 'GCA_017589495.2_CM030050.1:26883039_28884025', 'GCA_018492685.1_CM031723.1:28612962_30613948', 'GCA_023974075.1_CM043793.1:30252202_32253221', 'GCA_003711565.2_CM011073.1:9073220_11074185', 'GCA_024489055.1_CM044601.1:4483150_6484127', 'GCA_000633615.2_CM002714.1:6837127_8838104', 'GCA_902362185.1_LR697106.1:52198809_54377926', 'GCA_009769545.1_CM020122.1:9437176_11438159', 'GCA_007364275.2_CM017280.1:22009258_24010238', 'GCA_018320785.1_CM031343.1:5597392_7598372', 'GCA_029853015.1_CM056831.1:25645158_27646102', 'GCA_946959425.1_OX336890.1:578322_2579302', 'GCA_017976325.1_CM030617.1:26279613_28280611', 'GCA_000238955.5_CM009191.2:8273080_10274018', 'GCA_018340385.1_CM031268.1:3003497_5004453', 'GCA_002021735.2_CM007720.2:21781858_23782823', 'GCA_002021735.2_CM007720.2:25937792_27938769', 'GCA_018340385.1_CM031293.1:3422159_5423142', 'GCA_903798145.1_LR812495.1:46040431_48041405', 'GCA_900246225.5_LS420030.1:25650271_27651209', 'GCA_903798195.1_LR812526.1:10202677_12203651', 'GCA_024666655.1_CM045449.1:8430776_10431756'] | 349.9535 | 76 |
| Cluster4 | ['GCA_900700415.2_LR535858.1:27314793_29315818', 'GCA_001649575.2_CM027014.1:7465976_9466929', 'GCA_026122265.1_CM047516.1:12419848_14437165', 'GCA_949987685.1_OX465156.1:12991611_15011264', 'GCA_904848185.1_LR884468.1:18050855_20056391', 'GCA_936440315.1_OW387178.1:23717332_25718279', 'GCA_027123335.1_CM049576.1:22556436_24566658', 'GCA_022539595.1_CM040071.1:12376962_14387374', 'GCA_003047355.2_CM009719.1:23271657_25278268', 'GCA_900700375.2_LR535821.1:13066681_15080800', 'GCA_014839685.1_CM026075.1:25077690_27078643', 'GCA_949987555.1_OX465220.1:20178075_22179022', 'GCA_000972845.2_CM011632.1:8817665_10821556', 'GCA_003368295.1_CM010440.1:11425434_13426378', 'GCA_023724105.1_CM043232.1:21100067_23101011', 'GCA_027596085.2_CM053354.1:15229903_17230880', 'GCA_029620275.1_CM056144.1:9089360_11090451', 'GCA_027744805.2_CM050986.1:9392202_11398082', 'GCA_023566465.1_CM042227.1:8177789_10178751', 'GCA_020698575.1_CM036240.1:14496713_16499152', 'GCA_013358685.1_CM023618.1:9973100_11974038', 'GCA_021346845.1_CM037932.1:11813748_13820091', 'GCA_000721915.3_CM002841.3:18747318_20748247', 'GCA_004026145.2_CP034975.1:1925630_3937047', 'GCA_000633615.2_CM002707.1:34291117_36292031', 'GCA_003711565.2_CM011069.1:6272971_8285777', 'GCA_009769545.1_CM020134.1:13025475_15026452', 'GCA_902362185.1_LR697115.1:34644494_36658626', 'GCA_001660625.3_CM004419.2:27398276_29434048', 'GCA_017976325.1_CM030625.1:15548398_17550864', 'GCA_946959425.1_OX336895.1:10052285_12056411', 'GCA_018340385.1_CM031294.1:23012133_25013077', 'GCA_023376895.1_CM041799.1:12873976_14874923', 'GCA_903798145.1_LR812510.1:38804134_40826783', 'GCA_903798195.1_LR812527.1:46216419_48238789', 'GCA_023375975.1_CM041919.1:36718743_38806589', 'GCA_020184715.1_CM035063.1:39269278_41285313', 'GCA_012411365.1_CM022758.1:25302476_27303438', 'GCA_024256425.2_CM044236.1:27949026_29949979', 'GCA_024666655.1_CM045456.1:21839298_23848385'] | 224.02766 | 40 |
| Cluster5 | ['GCA_027887145.1_CM051184.1:35941611_37942576', 'GCA_929443795.2_OV839380.1:21671345_23672292', 'GCA_027579445.1_CM050582.1:34576836_36577801', 'GCA_907165065.1_OU015523.1:118985809_120986756', 'GCA_015476345.1_CM027243.1:200997073_202998008', 'GCA_013407035.1_CM023912.1:32147601_34148566', 'GCA_001522545.3_CM003709.1:81800249_83801211', 'GCA_019023105.1_CM032352.1:83266466_85267470', 'GCA_016128335.2_CM027945.2:5068011_7068946', 'GCA_002078875.2_CM007814.2:188079230_190080474', 'GCA_027559615.1_CM050532.1:33830333_35831295', 'GCA_009819825.1_CM020273.1:4670023_6670967', 'GCA_000738735.6_CM022185.2:31765801_33766766', 'GCA_910950805.1_OU383774.1:31729926_33730891', 'GCA_020740725.1_CM036347.1:35071881_37072846', 'GCA_009650955.1_CM018808.1:32549215_34550180', 'GCA_025583725.1_CM046733.1:53495715_55496650', 'GCA_001577835.2_CM003781.1:169951951_171952886', 'GCA_008658365.1_CM018076.1:210915179_212916126', 'GCA_009769605.1_CM020024.1:97380618_99381565', 'GCA_020740795.1_CM036503.1:4530760_6531707', 'GCA_900496995.4_LR606199.1:21105529_23106476', | 107.59182 | 33 |

| | | | |
|---|---|---|---|
| | 'GCA_013377495.2_CM044797.1:202325245_204326180', 'GCA_028858755.1_CM054378.1:127442017_129442940', 'GCA_023079485.1_CM041083.1:193592393_195593319', 'GCA_009769625.2_CM020065.2:202863094_204864029', 'GCA_009829145.1_CM020534.1:86189576_88190541', 'GCA_001746935.2_CM027507.1:29561999_31562916', 'GCA_018104995.1_CM030749.1:2734141_4735073', 'GCA_000146605.4_CM000962.2:184213563_186214498', 'GCA_017639555.1_CM030193.1:5074265_7075212', 'GCA_009819795.1_CM020300.1:200731755_202732690', 'GCA_029042245.1_CM055152.1:201554467_203555402'] | | |
| Cluster6 | ['GCA_029042245.1_CM055152.1:81123748_83124692', 'GCA_027358695.2_CM049780.1:32700181_34701089', 'GCA_901765095.2_LR594645.1:31902235_35360374', 'GCA_027887145.1_CM051184.1:31893796_33907971', 'GCA_027579445.1_CM050582.1:30519587_32536766', 'GCA_027789765.1_CM051063.1:14642971_16695835', 'GCA_929443795.2_OV839382.1:0_1457801', 'GCA_907165065.1_OU015523.1:19401857_21402780', 'GCA_015476345.1_CM027243.1:80564208_82565152', 'GCA_013407035.1_CM023912.1:28139544_30156822', 'GCA_001522545.3_CM003709.1:85715366_87724007', 'GCA_019023105.1_CM032352.1:87328707_89339071', 'GCA_016128335.2_CM027945.2:129798482_131799417', 'GCA_000090745.2_GL343381.1:0_1419372', 'GCA_027559615.1_CM050532.1:29644591_31645526', 'GCA_000738735.6_CM022185.2:27753712_29765036', 'GCA_910950805.1_OU383774.1:27640719_29659673', 'GCA_025388735.1_CM046214.1:112159682_114160626', 'GCA_020740725.1_CM036347.1:30977269_32990565', 'GCA_905171775.1_LR991687.1:959073_2980960', 'GCA_009650955.1_CM018808.1:28527515_30535356', 'GCA_025583725.1_CM046733.1:104474014_106474958', 'GCA_020745825.3_CM036715.2:88940128_90986874', 'GCA_018697195.1_CM031857.1:27073907_29074842', 'GCA_011800845.1_CM022354.1:23825477_26012197', 'GCA_003704135.2_CM010905.1:150542699_153437313', 'GCA_009667805.1_CM019068.1:28098136_30099107', 'GCA_003957565.4_CM012081.2:26015051_28025707', 'GCA_900496995.4_LR606187.1:0_1314354', 'GCA_013377495.2_CM044797.1:100128703_102129647', 'GCA_009769625.2_CM020065.2:100458229_102459173', 'GCA_009829145.1_CM020534.1:90213009_92213944', 'GCA_028564925.1_CM053036.1:2182453_4224859', 'GCA_018104995.1_CM030749.1:112526277_114527221', 'GCA_003829775.2_CM042573.1:26551755_28552714', 'GCA_901933205.1_LR595696.1:86784398_88789606', 'GCA_009819795.1_CM020300.1:81127903_83128847'] | 351.69654 | 37 |
| Cluster7 | ['GCA_028533215.1_CM052696.1:4975217_8177184', 'GCA_009834535.1_CM020774.1:16854143_20036768', 'GCA_000165445.3_CM007671.1:9238366_12425559', 'GCA_905319855.2_HG994398.1:53413133_56690107', 'GCA_027475565.2_CM050184.1:11073255_14052379', 'GCA_002201575.2_CM035908.1:92298611_94299561', 'GCA_029890205.1_CM057028.1:83840821_85922511', 'GCA_022682495.1_CM040293.1:83799306_86687820', 'GCA_020826845.1_CM036948.1:88001675_90142918', 'GCA_907164435.1_OU015376.1:78807008_80811024', 'GCA_002863925.1_CM009151.1:93678830_95685176', 'GCA_903995435.1_LR877221.1:1029903_3114521', 'GCA_021442165.1_CM038152.1:13468197_15625654', 'GCA_028627145.1_CM053514.1:67189490_69839742', 'GCA_002007445.3_CM022819.1:199121859_202290460', 'GCA_019320065.1_CM033193.1:98446258_100616521', 'GCA_910594005.1_OU343095.1:109771735_111772685', 'GCA_947179515.1_OX359298.1:63817199_66001031', 'GCA_028646485.1_CM053559.1:87781115_90898485', 'GCA_010411085.1_CM021209.1:97173612_100574332', 'GCA_011762505.1_CM022265.1:21965824_24066653', 'GCA_030015415.1_CM057393.1:4088893_6798018', 'GCA_028533335.1_CM052477.1:46514071_49596085', 'GCA_001883655.1_CM007428.1:5230089_8435335', 'GCA_000803125.3_CM016633.2:73516544_75625140'] | 94.01 | 25 |
| Cluster8 | ['GCA_902713425.2_OV754670.1:103704208_105813088', 'GCA_000242695.1_CM001407.1:35789853_37790839'] | 349.9535 | 2 |
| Cluster9 | ['GCA_902713425.2_OV754630.1:1968848_3969795'] | NA | 1 |
| Cluster1<br>0 | ['GCA_902713425.2_OV754682.1:8455925_10456893'] | NA | 1 |
| Cluster1<br>1 | ['GCA_902713425.2_OV754684.1:24225446_26226375'] | NA | 1 |
| Cluster1<br>2 | ['GCA_020745825.3_CM036716.1:69836759_71844280', 'GCA_027887145.1_CM051185.1:72551655_74558706', 'GCA_027579445.1_CM050583.1:69773350_71780419', 'GCA_019023105.1_CM032355.1:70464731_72465684'] | 24.20891 | 4 |
| Cluster1<br>3 | ['GCA_027887145.1_CM051185.1:111106238_113154336', 'GCA_027579445.1_CM050583.1:108274434_110317175', 'GCA_020745825.3_CM036716.1:108913806_110932045', 'GCA_003829775.2_CM042576.1:106233396_108234367', 'GCA_901933205.1_LR595697.1:106003242_108007709'] | 24.20891 | 5 |
| Cluster1<br>4 | ['GCA_018697195.1_CM031859.1:3257559_5258509', 'GCA_008658365.1_CM018078.1:117676055_119677020', 'GCA_015476345.1_CM027245.1:113480032_115496294', 'GCA_004027225.2_CM013767.2:56151595_58152545', 'GCA_009769605.1_CM020024.1:50790451_52791404', 'GCA_003957555.2_CM012116.1:108234763_110265544', 'GCA_015227895.2_CM037641.1:18711171_20717940', 'GCA_002078875.2_CM007816.2:110002027_112003514', 'GCA_018104995.1_CM030751.1:113716966_115918926', 'GCA_003957565.4_CM012084.2:5031228_7043593', 'GCA_020740795.1_CM036505.1:108476514_110542816', 'GCA_028858755.1_CM054379.1:18963552_20964502', 'GCA_907165065.1_OU015524.1:117091703_119107369', 'GCA_009819795.1_CM020302.1:113027714_115033910', 'GCA_029042245.1_CM055154.1:113912618_115918821'] | 90.84966 | 15 |
| Cluster1<br>5 | ['GCA_017589495.2_CM030048.1:33659688_35660620'] | NA | 1 |
| Cluster1<br>6 | ['GCA_018492685.1_CM031717.1:2773418_4774350'] | NA | 1 |
| Cluster1<br>7 | ['GCA_001660625.3_CM004419.2:11510333_13511271', 'GCA_024256425.2_CM044236.1:7241144_9242085', 'GCA_012411365.1_CM022758.1:8951798_10952736'] | 61.2658 | 3 |
| Cluster1<br>8 | ['GCA_017591415.1_CM030127.1:31825655_33826629', 'GCA_000242695.1_CM001404.1:12234574_14235548'] | 297.35 | 2 |
| Cluster1<br>9 | ['GCA_001640805.2_CM044665.1:24861748_26862722', 'GCA_027596085.2_CM053351.1:26145508_28146641', 'GCA_027123335.1_CM049572.1:2250996_4251964', 'GCA_022539595.1_CM040066.1:2041646_4042608', 'GCA_004026145.2_CP034991.1:12235604_14236524', 'GCA_003711565.2_CM011073.1:11133171_13134091', 'GCA_018320785.1_CM031343.1:52347400_54348518', 'GCA_024666655.1_CM045449.1:2262815_4476872'] | 112.24405 | 8 |
| Cluster2<br>0 | ['GCA_018555375.2_CM031788.1:27634352_29635326'] | NA | 1 |
| Cluster2<br>1 | ['GCA_018555375.2_CM031795.1:19728189_21729145'] | NA | 1 |
| Cluster2<br>2 | ['GCA_011800845.1_CM022340.1:66025750_68049047', 'GCA_000090745.2_CM000938.1:148645374_150759441'] | 166.90373 | 2 |
| Cluster2<br>3 | ['GCA_000090745.2_CM000938.1:151373450_153415190'] | NA | 1 |
| Cluster2<br>4 | ['GCA_000090745.2_CM000938.1:146312068_148312976'] | NA | 1 |

| | | | |
|---|---|---|---|
| Cluster25 | ['GCA_027563665.1_CM050521.1:31077549_34049962'] | NA | 1 |
| Cluster26 | ['GCA_017639675.1_CM030177.1:1336768_4245784'] | NA | 1 |
| Cluster27 | ['GCA_003704035.3_CM010881.2:23328954_25329985', 'GCA_020826845.1_CM036948.1:71957044_73957949', 'GCA_027409185.1_CM050032.1:31862799_33863698', 'GCA_028533215.1_CM052696.1:21844274_23845176', 'GCA_019923935.1_CM034278.1:85924768_87925757', 'GCA_011762505.1_CM022265.1:37544322_39545221', 'GCA_009873245.3_CM020949.2:27601372_29602274', 'GCA_019320065.1_CM033193.1:81791582_83792571', 'GCA_030015415.1_CM057393.1:16591713_18592618', 'GCA_003704135.2_CM010905.1:16334164_18335195', 'GCA_903992535.2_LR862381.2:170323568_172324449', 'GCA_947179515.1_OX359298.1:46753414_48754397', 'GCA_010411085.1_CM021209.1:80588168_82589157', 'GCA_002263795.3_CM008171.2:86407411_88408400', 'GCA_021498455.1_CM038419.1:61844372_63845247', 'GCA_000003025.6_CM000829.5:23286430_25287332'] | 94.01 | 16 |
| Cluster28 | ['GCA_004786255.1_CM016419.1:75222_2373688', 'GCA_027789765.1_CM051061.1:150740971_152768384', 'GCA_023079485.1_CM041085.1:115770084_117771043', 'GCA_004786255.1_CM016419.1:0_1975936'] | 351.69654 | 4 |
| Cluster29 | ['GCA_023375975.1_CM041920.1:12227842_14228816'] | NA | 1 |
| Cluster30 | ['GCA_023375975.1_CM041926.1:11720862_13721878'] | NA | 1 |
| Cluster31 | ['GCA_014182915.2_CM024438.1:71660183_73661073'] | NA | 1 |
| Cluster32 | ['GCA_005887515.3_CM016693.1:15179986_17194204'] | NA | 1 |
| Cluster33 | ['GCA_011100555.2_CM021923.1:9072827_12256149'] | NA | 1 |
| Cluster34 | ['GCA_901933205.1_LR595697.1:44950823_46951776'] | NA | 1 |
| Cluster35 | ['GCA_022682495.1_CM040290.1:176913157_178946638', 'GCA_009834535.1_CM020801.1:13584999_15623518'] | 81.03605 | 2 |
| Cluster36 | ['GCA_000978405.1_CM003217.1:99238004_101396705'] | NA | 1 |
| Cluster37 | ['GCA_003368295.1_CM010439.1:22119494_24120459'] | NA | 1 |
| Cluster38 | ['GCA_003368295.1_CM010439.1:3870888_5871871'] | NA | 1 |
| Cluster39 | ['GCA_003368295.1_CM010440.1:18974051_20974971'] | NA | 1 |
| Cluster40 | ['GCA_009819825.1_CM020257.1:1125214_3126149'] | NA | 1 |
| Cluster41 | ['GCA_028627265.1_CM053391.1:34420736_36459954'] | NA | 1 |
| Cluster42 | ['GCA_902362185.1_LR697115.1:3701776_5706554'] | NA | 1 |
| Cluster43 | ['GCA_018320785.1_CM031353.1:19878243_21879175'] | NA | 1 |
| Cluster44 | ['GCA_024256425.2_CM044240.1:21888442_23889416'] | NA | 1 |
| Cluster45 | ['GCA_900700415.2_LR535863.1:24838071_26839057'] | NA | 1 |
| Cluster46 | ['GCA_027475355.1_CM050217.1:2621085_4622095'] | NA | 1 |
| Cluster47 | ['GCA_902810595.1_LR778256.1:64878457_66879404'] | NA | 1 |
| Cluster48 | ['GCA_001577835.2_CM003783.1:99150140_101151114'] | NA | 1 |
| Cluster49 | ['GCA_017976375.1_CM030637.1:18473441_20474388'] | NA | 1 |
| Cluster50 | ['GCA_009769625.2_CM020067.2:5369771_7370724'] | NA | 1 |
| Cluster51 | ['GCA_027789765.1_CM051060.1:128668651_130704100'] | NA | 1 |
| Cluster52 | ['GCA_027789765.1_CM051061.1:116229846_119839599'] | NA | 1 |
| Cluster53 | ['GCA_900700375.2_LR535832.1:15307026_17336174'] | NA | 1 |
| Cluster54 | ['GCA_016128335.2_CM027947.2:6426861_8427913'] | NA | 1 |
| Cluster55 | ['GCA_900747795.4_LR536447.2:80234298_82235230'] | NA | 1 |
| Cluster56 | ['GCA_000721915.3_CM002845.3:36349018_38354993'] | NA | 1 |
| Cluster57 | ['GCA_000721915.3_CM002842.3:31208516_33209439'] | NA | 1 |
| Cluster58 | ['GCA_027917425.1_CM051235.1:400858718_402934124'] | NA | 1 |
| Cluster59 | ['GCA_017654675.1_CM030348.1:167685602_169770821', 'GCA_027917425.1_CM051237.1:55520477_57561766', 'GCA_027358695.2_CM049772.1:117909553_120025216', 'GCA_000004195.4_CM004447.2:158983992_161049499'] | 202.33808 | 4 |
| Cluster60 | ['GCA_001660625.3_CM004418.2:13099251_15100225'] | NA | 1 |

| | | | |
|---|---|---|---|
| Cluster61 | ['GCA_000972845.2_CM011638.1:17923207_19924154'] | NA | 1 |
| Cluster62 | ['GCA_009667805.1_CM019063.1:197376344_199555333'] | NA | 1 |
| Cluster63 | ['GCA_009667805.1_CM019075.1:15113975_17515951'] | NA | 1 |
| Cluster64 | ['GCA_002021735.2_CM007719.2:53677541_55761513'] | NA | 1 |
| Cluster65 | ['GCA_004115215.4_CM014212.1:36537979_38672944'] | NA | 1 |
| Cluster66 | ['GCA_001700915.1_CM004530.1:106416840_108417793'] | NA | 1 |
| Cluster67 | ['GCA_016835505.1_CM029072.1:23158242_25159174'] | NA | 1 |
| Cluster68 | ['GCA_004786255.1_CM016419.1:3590084_6891944'] | NA | 1 |
| Cluster69 | ['GCA_004786255.1_CM016422.1:95302_4146469'] | NA | 1 |
| Cluster70 | ['GCA_004786255.1_CM016425.1:8147610_10151526'] | NA | 1 |
| Cluster71 | ['GCA_000004195.4_CM004449.2:17672003_19862864', 'GCA_017654675.1_CM030352.1:12200406_14418027', 'GCA_004786255.1_CM016425.1:25762845_27938909', 'GCA_024363595.1_CM044443.1:12518767_14782659'] | 202.33808 | 4 |
| Cluster72 | ['GCA_027358695.2_CM049776.1:45816763_47922844'] | NA | 1 |
| Cluster73 | ['GCA_027358695.2_CM049778.1:19071057_21155182'] | NA | 1 |
| Cluster74 | ['GCA_027358695.2_CM049780.1:20046836_22179757'] | NA | 1 |
| Cluster75 | ['GCA_024363595.1_CM044444.1:75719962_77742789', 'GCA_000004195.4_CM004449.2:95759279_97760190'] | 58.2299 | 2 |
| Cluster76 | ['GCA_017654675.1_CM030349.1:140798082_142855104'] | NA | 1 |
| Cluster77 | ['GCA_017654675.1_CM030353.1:83427968_85505040'] | NA | 1 |
| Cluster78 | ['GCA_000004195.4_CM004451.2:13045211_15046158'] | NA | 1 |
| Cluster79 | ['GCA_011800845.1_CM022341.1:111505559_113506707'] | NA | 1 |
| Cluster80 | ['GCA_011800845.1_CM022354.1:19394926_21395915'] | NA | 1 |
