# Supplementary material for: Rapid expansion and specialization of the TAS2R bitter taste receptor family in amphibians
Source: PLoS Genet. 2025 Jan 31;21(1):e1011533. doi: 10.1371/journal.pgen.1011533 (PMC11798467; doi:10.1371/journal.pgen.1011533)
Supplement: S4 Table — Alphabetical by compound, with classic bitterants shown first and project-specific natural products at end. (PDF) [file pgen.1011533.s027.pdf]

	CAS RN	Vendor	Concentration (µM)
(-)-α-thujone	546-80-5	Santa Cruz Biotechnologies	200
Amarogentin	21018-84-8	BLD pharmatech	1000
Arbutin	497-76-7	Tokyo Chemical Industry	30000
Aristolochic acid I sodium salt	10190-99-5	Sigma-Aldrich	10
(+)-camphor	464-49-3	FUJIFILM Wako Pure Chemical	750
Chloramphenicol	56-75-7	FUJIFILM Wako Pure Chemical	1000
Chloroquine diphosphate	50-63-5	FUJIFILM Wako Pure Chemical	10000
Colchicine	64-86-8	FUJIFILM Wako Pure Chemical	3000
Coumarin	91-64-5	Tokyo Chemical Industry	300
Denatonium benzoate	3734-33-6	FUJIFILM Wako Pure Chemical	3000
N,N',-Diphenylthiourea	0102-08-09	Tokyo Chemical Industry	100
Genistein	446-72-0	Tokyo Chemical Industry	10
Helicin	618-65-5	Tokyo Chemical Industry	10000
Papaverine hydrochloride	61-25-6	FUJIFILM Wako Pure Chemical	10
Picrotoxin	124-87-8	FUJIFILM Wako Pure Chemical	750
Phenylthiocarbamide (PTC)	103-85-5	Nacalai tesque	100
6-n-propylthiouracil (PROP)	51-52-5	Nacalai tesque	500
Quinine hydrochloride dihydrate	6119-47-7	FUJIFILM Wako Pure Chemical	10
Salicin	138-52-3	Tokyo Chemical Industry	10000
Strychnine nitrate	66-32-0	FUJIFILM Wako Pure Chemical	20
Xanthotoxin	298-81-7	Tokyo Chemical Industry	100
Yohimbine hydrochloride	65-19-0	FUJIFILM Wako Pure Chemical	200
Aflatoxin B1	1162-65-8	Fermentek	50
Batrachotoxin	23509-16-2	Latoxan	5
Cinobufagin	470-37-1	Tokyo Chemical Industry	50
Heliotrine	303-33-3	Latoxan	500
Marinobufagenin	470-42-8	Cayman chemical	50
Swainsonine	72741-87-8	Cayman chemical	100
