# Supplementary material for: Rapid expansion and specialization of the TAS2R bitter taste receptor family in amphibians
Source: PLoS Genet. 2025 Jan 31;21(1):e1011533. doi: 10.1371/journal.pgen.1011533 (PMC11798467; doi:10.1371/journal.pgen.1011533)
Supplement: S5 Table — The comparison of intact TAS2Rs in an axolotl genome assembly GCA_002915635.2 between this study and Behrens et al. 2021 [27]. (PDF) [file pgen.1011533.s028.pdf]

Behrens et al. 2021	This study	comment
Amme-T2R1	axolotl_axolotl_5_aa_144602027-1	
Amme-T2R10	axolotl_axolotl_5_aa_68660131-68	
Amme-T2R11	axolotl_PGSH01042166.1_5831-6790	
Amme-T2R12	NA	only 5 TM helices
Amme-T2R13	axolotl_axolotl_5_aa_3180807-318	
Amme-T2R16	axolotl_axolotl_5_aa_68525411-68	
Amme-T2R17	axolotl_axolotl_5_aa_68337860-68	
Amme-T2R18	axolotl_axolotl_5_aa_68319911-68	
Amme-T2R22	NA	starts with a stop
Amme-T2R23	axolotl_PGSH01103005.1_33513-344	
Amme-T2R24	axolotl_PGSH01095381.1_45471-464	
Amme-T2R25	axolotl_PGSH01072308.1_16166-171	
Amme-T2R26	axolotl_PGSH01001217.1_12154-132	
Amme-T2R27	NA	has 7TM helices but first right at the beginning
Amme-T2R28	axolotl_axolotl_5_aa_42999280-43	
Amme-T2R29	NA	only 6 TM helices
Amme-T2R3	axolotl_axolotl_5_aa_119278715-1	
Amme-T2R30	axolotl_axolotl_12_ab_486155050-	
Amme-T2R31	axolotl_axolotl_16_aa_447317813-	
Amme-T2R34	axolotl_axolotl_5_aa_121350872-1	
Amme-T2R37	axolotl_axolotl_8_aa_47594974-47	
Amme-T2R39	axolotl_axolotl_5_aa_125500661-1	
Amme-T2R4	NA	starts with an N
Amme-T2R40	axolotl_axolotl_5_ab_275914048-2	
Amme-T2R41	axolotl_axolotl_5_ab_275635533-2	
Amme-T2R43	axolotl_axolotl_19_aa_264991016-	
Amme-T2R44	axolotl_axolotl_19_aa_276667455-	
Amme-T2R47	NA	has 7TM helices but first right at the beginning
Amme-T2R52	axolotl_axolotl_10_ab_243212079-	
Amme-T2R56	NA	
Amme-T2R62	axolotl_axolotl_16_ab_216226967-	
Amme-T2R66	NA	starts with a stop
Amme-T2R68	axolotl_axolotl_16_ab_216688397-	
Amme-T2R69	axolotl_PGSH01045501.1_16498-175	
Amme-T2R7	axolotl_axolotl_15_aa_229884289-	
Amme-T2R70	axolotl_axolotl_16_ac_503405835-	
Amme-T2R71	NA	only 5 TM helices
Amme-T2R80	axolotl_axolotl_16_ab_216399719-	
Amme-T2R81	axolotl_axolotl_8_aa_157565154-1	
Amme-T2R82	axolotl_axolotl_13_ab_56774184-5	
Amme-T2R83	axolotl_axolotl_3_aa_9577404-957	
Amme-T2R84	NA	contains a stop
Amme-T2R85	axolotl_PGSH01071979.1_16726-176	
Amme-T2R89	axolotl_axolotl_7_aa_114911921-1	
Amme-T2R90	axolotl_PGSH01119064.1_43369-443	
NA	axolotl_axolotl_16_ab_216946875-	
NA	axolotl_axolotl_5_aa_35026915-35	
NA	axolotl_axolotl_7_aa_132027244-1	
NA	axolotl_PGSH01086352.1_12293-132	
NA	axolotl_PGSH01111417.1_7100-8059	

References

1. Behrens M, Di Pizio A, Redel U, Meyerhof W, Korsching SI. At the Root of *T2R* Gene Evolution: Recognition Profiles of Coelacanth and Zebrafish Bitter Receptors. Mar A, editor. Genome Biol Evol. 2021;13: evaa264. doi:10.1093/gbe/evaa264
