# Supplementary material for: Rapid expansion and specialization of the TAS2R bitter taste receptor family in amphibians
Source: PLoS Genet. 2025 Jan 31;21(1):e1011533. doi: 10.1371/journal.pgen.1011533 (PMC11798467; doi:10.1371/journal.pgen.1011533)
Supplement: S6 Table — These species were present in our TAS2R database but an exact match was not available on TimeTree. We substituted a closely related species as indicated in this table. (PDF) [file pgen.1011533.s029.pdf]

	**Surrogate taxon on the phylogeny**	**Notes**
Pristis pectinata	*Pristis clavata*	Replaced automatically by the TimeTree portal
Lampris incognitus	*Lampris guttatus*	Replaced automatically by the TimeTree portal
Pseudoliparis swirei	*Liparis tanakae*	Replaced automatically by the TimeTree portal
Cottoperca gobio	*Cottoperca*	Replaced automatically by the TimeTree portal
Anableps anableps	*Anableps*	Replaced automatically by the TimeTree portal
Paedocypris micromegethes	*Paedocypris sp. Banka*	Replaced automatically by the TimeTree portal
Albula goreensis	*Albula vulpes*	Replaced automatically by the TimeTree portal
Pseudophryne corroboree	*Pseudophryne bibronii*	Replaced automatically by the TimeTree portal
Hemiprocne comata	*Hemiprocne*	Replaced automatically by the TimeTree portal
Ciconia maguari	*Ciconia ciconia*	Replaced automatically by the TimeTree portal
Phoenicopterus ruber	*Phoenicopterus*	Replaced automatically by the TimeTree portal
Merops nubicus	*Merops apiaster*	Replaced automatically by the TimeTree portal
Indicator indicator	*Indicator*	Replaced automatically by the TimeTree portal
Pogoniulus pusillus	*Pogoniulus*	Replaced automatically by the TimeTree portal
Colius striatus	*Colius*	Replaced automatically by the TimeTree portal
Prinia subflava	*Prinia bairdii*	Replaced automatically by the TimeTree portal
Certhia americana	*Certhia brachydactyla*	Replaced automatically by the TimeTree portal
Lichenostomus cassidix	*Ptiloprora plumbea*	Replaced automatically by the TimeTree portal
Otis tarda	*Chlamydotis undulata*	Replaced automatically by the TimeTree portal
Trachemys scripta	*Trachemys dorbigni*	Replaced automatically by the TimeTree portal
Protopterus annectens	*Protopterus*	Replaced automatically by the TimeTree portal
Rattus rattus	*Rattus rattus* complex lineage III	Replaced manually. All *R. rattus* lineages were equally close to the nearest relative on our tree.
Stegostoma fasciatum	*Stegostomatidae*	Replaced manually. Only *Stegostomatidae* was available on TimeTree. This is the only extant species in Stegostomatidae.
Phalacrocorax aristotelis	*Phalacrocoracidae*	Replaced manually. Only *Phalacrocoracidae* was available on TimeTree. This species was the only Phalacrocoracid in our dataset.
Kryptolebias hermaphroditus	*Kryptolebias ocellatus*	Replaced manually. This was the closest species available to *K. hermaphroditus* on TimeTree (https://doi.org/10.1016/j.ympev.2022.107617)
Oenanthe melanoleuca	*Oenanthe finschii*	Replaced manually due to the existence of a homonymous plant genus.
Balaenoptera ricei	*Balaenoptera edeni*	Replaced manually. *B. edeni* is *B. ricei*'s closest relative on TimeTree based on https://doi.org/10.1111/mms.12776
Ammodytes marinus	*Ammodytes hexapterus*	Replaced manually. *A. hexapterus* is its closest relative on TimeTree based on https://doi.org/10.7755/FB.113.2.3
Triplophysa bombifrons	*Triplophysa stoliczkai*	Replaced manually. *T stoliczkai* is *T. bombifrons*'s closest relative on TimeTree based on https://doi.org/10.3390/genes14071356
Triplophysa tibetana	*Triplophysa stenura*	Replaced manually. *T. ribetana* is its closest relative on TimeTree based on https://doi.org/10.3390/genes14071356
Gopherus evgoodei	*Gopherus morafkai*	Replaced manually. *G. morafkai* is its closest relative on TimeTree based on http://dx.doi.org/10.3897/zookeys.562.6124
Taenioides_sp._WSHXM2023	*Taenioides limicola*	Replaced manually. *T. limicola* was the only Taenioides available on TimeTree.
Ilyophis_sp._1_JC-2022 Ilyophis_sp._2_JC-2022 Ilyophis_sp._JC_2022_1	*Ilyophis brunneus*	These three species were collapsed into a single taxon named *I. bruneus*, which is the only Ilyophis on TimeTree. We found the same number of TAS2R genes in these three species' genomes, so collapsing them into a single taxon should not have a major effect on our results.
Epinephelus moara	*Epinephelus bruneus*	Replaced manually. . *E. bruneus* is closely related and often considered synonymous with *E. morara* (https://doi.org/10.1111/jfb.12112).

Pseudocheirus occidentalis	*Pseudocheirus peregrinus*	Replaced manually, *P. peregrinus* was the only congeneric species on TimeTree.
Eidolon dupreanum	*Eidolon helvum*	Replaced manually. *E. helvum* was the only congeneric species on TimeTree, and is closely related as per https://doi.org/10.1093/sysbio/syab013
Pangasius djambal	*Pangasius bocourti*	Replaced manually with *P. bocourti*, which is closely related to *P. djambal* (https://doi.org/10.3390/hydrobiology2020028)
Lamprotornis superbus	*Lamprotornis bicolor*	Replaced manually. *L. bicolor* is closely related to *L. superbus* based on https://doi.org/10.1016/j.cub.2007.07.032
Acrossocheilus wenchowensis	*Acrossocheilus fasciatus*	Replaced manually. *A. fasciatus* is its closest relative on TimeTree based on https://doi.org/10.1080/23802359.2019.1667916
Siphamia tubifer	*Siphamia versicolor*	Replaced manually. This is the only congeneric named species available on TimeTree. There is an unidentified species assigned to the same genus that falls elsewhere in the tree but we favored S. versicolor, since it has been identified to the species level.
Seriola aureovittata	*Seriola lalandi*	Replaced manually. *S. aureovittata* was previously considered a subspecies of lalandi, and was later re-elevated to species status (http://dx.doi.org/10.1643/CI-124-224).
Chiloscyllium plagiosum	*Chiloscyllium punctatum*	Replaced manually. This was the only congeneric species on TimeTree, and is closely related to *C. plagiosum* (http://dx.doi.org/10.1155/2014/213896)
Harpia harpyja	NA	Species not included in tree. No suitable replacement was found.
Rhincodon typus	NA	Species not included in tree. No suitable replacement was found.
Nettapus auritus	NA	Species not included in tree. No suitable replacement was found.
Notolabrus celidotus	NA	Species not included in tree. No suitable replacement was found.
Heteronetta atricapilla	NA	Species not included in tree. No suitable replacement was found.
Salvelinus sp. IW2-2015	NA	Species not included in tree. No suitable replacement was found.
Gasterosteus nipponicus	NA	Species not included in tree. No suitable replacement was found.
Ammospiza nelsoni	NA	Species not included in tree. No suitable replacement was found.
Aythya baeri	NA	Species not included in tree. No suitable replacement was found.
Hemiscyllium ocellatum	NA	Species not included in tree. No suitable replacement was found.
Leucoraja erinacea	NA	Species not included in tree. No suitable replacement was found.
Stictonetta naevosa	NA	Species not included in tree. No suitable replacement was found.
Hyperoplus immaculatus	NA	Species not included in tree. No suitable replacement was found.
Triplophysa dalaica	NA	Species not included in tree. No suitable replacement was found.
Oryzias melastigma	NA	Species not included in tree. No suitable replacement was found.
