# Supplementary material for: Rapid expansion and specialization of the TAS2R bitter taste receptor family in amphibians
Source: PLoS Genet. 2025 Jan 31;21(1):e1011533. doi: 10.1371/journal.pgen.1011533 (PMC11798467; doi:10.1371/journal.pgen.1011533)
Supplement: S7 Table — Model formula was: log(Number.of.Genes.x+1)~log(clusters+1)+log(genes_per_cluster+1)+log(genome_size) (PDF) [file pgen.1011533.s030.pdf]

	Estimate (Œ≤)	StdErr	t.value	p.value
(Intercept)	-0.204614	0.06300217	-3.2477289	0.00144338
log(clusters + 1)	1.07695345	0.03859231	27.9059051	2.16E-60
log(genes_per_cluster + 1)	0.91431118	0.02871821	31.837328	1.41E-67
log(genome_size)	0.05409877	0.03111939	1.73842669	0.08424382
